# Large-magnitude (VEI ≥ 7) ‘wet’ explosive silicic eruption preserved a Lower Miocene habitat at the Ipolytarnóc Fossil Site, North Hungary

**DOI:** 10.1038/s41598-022-13586-3

**Published:** 2022-06-13

**Authors:** Dávid Karátson, Tamás Biró, Maxim Portnyagin, Balázs Kiss, Jean-Louis Paquette, Zoltán Cseri, Mátyás Hencz, Károly Németh, Pierre Lahitte, Emő Márton, László Kordos, Sándor Józsa, Lilla Hably, Samuel Müller, Imre Szarvas

**Affiliations:** 1 Department of Physical Geography, Eötvös University, Budapest, Hungary; 2 GEOMAR Helmholtz Centre for Ocean Research, Kiel, Germany; 3 Department of Petrology and Geochemistry, Eötvös University, Budapest, Hungary; 4 Laboratoire Magmas et Volcans, Université Clermont Auvergne, Clermont-Ferrand, France; 5 School of Agriculture and Environment, Massey University, Palmerston North, New Zealand; 6 Institute of Earth Physics and Space Science, Sopron, Hungary; 7 Université Paris-Saclay, CNRS, UMR GEOPS, 91405 Orsay, France; 8 Mining and Geological Survey of Hungary, Paleomagnetic Laboratory, Budapest, Hungary; 9 Eötvös University, Savaria University Centre, Szombathely, Hungary; 10 Botanical Department, Hungarian Natural History Museum, Budapest, Hungary; 11 Institute of Earth Sciences, Christian-Albrecht University of Kiel, Kiel, Germany; 12 Ipolytarnóc Fossils Nature Conservation Area, Ipolytarnóc, Hungary

**Keywords:** Natural hazards, Solid Earth sciences

## Abstract

During Earth’s history, geosphere-biosphere interactions were often determined by momentary, catastrophic changes such as large explosive volcanic eruptions. The Miocene ignimbrite flare-up in the Pannonian Basin, which is located along a complex convergent plate boundary between Europe and Africa, provides a superb example of this interaction. In North Hungary, the famous Ipolytarnóc Fossil Site, often referred to as “ancient Pompeii”, records a snapshot of rich Early Miocene life buried under thick ignimbrite cover. Here, we use a multi-technique approach to constrain the successive phases of a catastrophic silicic eruption (VEI ≥ 7) dated at 17.2 Ma. An event-scale reconstruction shows that the initial PDC phase was phreatomagmatic, affecting ≥ 1500 km^2^ and causing the destruction of an interfingering terrestrial–intertidal environment at Ipolytarnóc. This was followed by pumice fall, and finally the emplacement of up to 40 m-thick ignimbrite that completely buried the site. However, unlike the seemingly similar AD 79 Vesuvius eruption that buried Pompeii by hot pyroclastic density currents, the presence of fallen but uncharred tree trunks, branches, and intact leaves in the basal pyroclastic deposits at Ipolytarnóc as well as rock paleomagnetic properties indicate a low-temperature pyroclastic event, that superbly preserved the coastal habitat, including unique fossil tracks.

Catastrophic explosive silicic eruptions may affect the landscape in tens of thousands of km^2^ in a short time, and impact the paleoenvironment as natural catastrophes^1,2^. These large-magnitude eruptions are often associated with grabens hosting caldera clusters (e.g. Kagoshima Bay, Southern Kyushu, Japan^3^; Taupō Volcanic Zone, New Zealand^4^). The Pannonian Basin (Central Europe), representing a complex convergent plate tectonic setting that belongs to the Mediterranean region, experienced repetitive explosive silicic eruptions in Miocene times^5–8^. Producing large-volume ignimbrites and other pyroclastic sequences, these eruptions, which occurred in the Paratethys with an archipelago and rich subtropical vegetation on land^9^, certainly impacted the ecosystem. However, due to intense neotectonic movements and related erosion that resulted in a poor preservation of pyroclastic rocks, very little information is available on the contemporary habitats that were commonly buried by thick pyroclastic deposits.

A rare example of contemporary life is the famous Ipolytarnóc Fossil Site^10^, which has been considered an “ancient Pompeii”^11^ as it was disrupted and buried by an ignimbrite eruption c. 17–17.5 Ma^12,13^. Although it has been known since 1836^14^ and holds an European Diploma for Protected Areas^15^, the specific eruption that affected the area has not been identified and characterized. In general, despite recent progress to date major eruptive units in the Pannonian Basin^16,17^, a detailed event stratigraphy of the ignimbrites that occur over large areas has not yet been reconstructed. To fill these gaps, herein we present new findings of the initiation of ignimbrite flare-up in the Pannonian Basin between 18 and 17 Ma. In particular, we focus on how a highly explosive, high-intensity, large-volume, multi-phase eruption impacted the peculiar habitat of Ipolytarnóc, by reconstructing the volcanic succession in time and space through field volcanology, pumice geochemistry, pyroclast texture characterization via BSE imaging, and Ar–Ar and U–Pb dating.

## Lower Miocene ignimbrite eruptions in a diverse, densely vegetated archipelago

**Figure 1:**
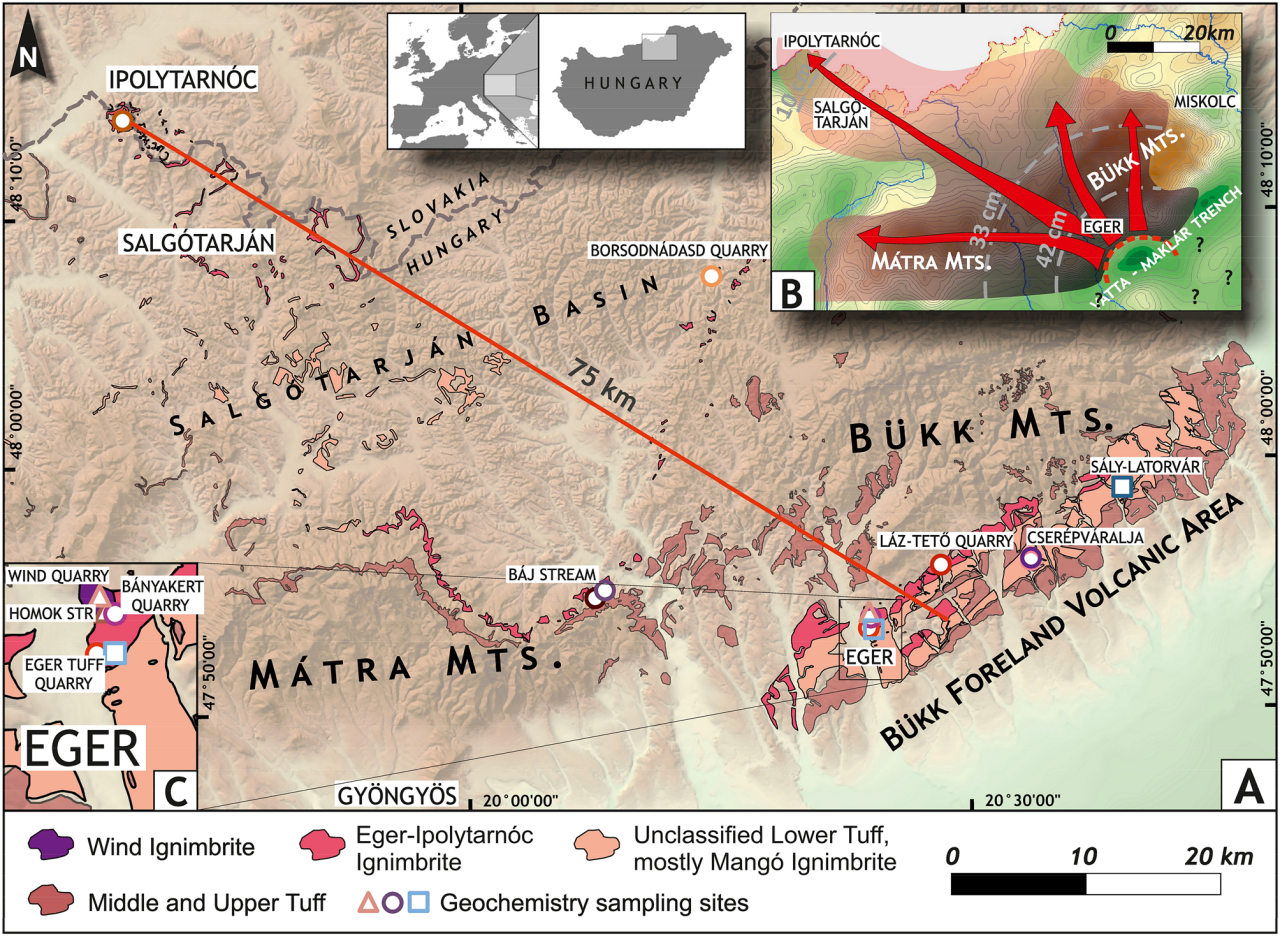
Areal distribution of the main pyroclastic formations: the Lower, Middle and Upper Tuff in the Northern Pannonian Basin (Hungary) draped over a shaded relief image derived from the 50 m DEM of Hungary (**A**). Source of surface occurrences: Mining and Geological Survey of Hungary MBFSZ 1:100,000 geological map (https://map.mbfsz.gov.hu/). (Image created with Surfer 13, version 13.0.383 Golden Software.) Initiation of the ignimbrite flare-up is represented by the Wind Ignimbrite (confined to Eger Northeast) and the subsequent, widespread Eger-Ipolytarnóc Ignimbrite (the focus of this study). As for the latter, the average distance between the medial Eger occurrences and the distal Ipolytarnóc site is c. 75 km (red line). Inset map top right (**B**) shows the possible distribution of the first-phase pyroclastic surge and the late-stage pumiceous pyroclastic flow from the Eger-Ipolytarnóc eruption, draped over the Bouguer gravity anomaly map of Hungary (MBFSZ 1:500,000). Possible isopach lines of the third-phase pumice-fall deposits of the eruption, based on three sites (see main text), are also indicated. A marked negative gravity anomaly southeast of Eger town, as part of the Vatta-Maklár trench, is in accordance with a potential caldera as source vent^6^. Inset map bottom left (**C**) shows a close-up of the vicinity of Eger with locations mentioned in text.

The Pannonian Basin, located within the Carpathian Mountains, belongs to the Mediterranean-Alpine orogeny realm^7–9^. Subduction- and/or collision-related volcanism occurred along the Carpathian arc from the Mid-Miocene to the Late Quaternary^18–20^, whereas the basin itself was an area of intense explosive silicic volcanism throughout the Miocene (c. 20–12 Ma^5,6,16–18,21^). Basin subsidence, neotectonic faulting, block uplift, and related intense erosion^22^ resulted in the disintegration and removal of most pyroclastic rocks. The stratigraphy has been traditionally divided into Lower, Middle and Upper Tuff Formations^23^, that occur in scattered locations (Fig. 1) and have been confirmed by basin-wide (500 km across) borehole information^16^. In addition to poor surface preservation, their correlation is hindered by the texturally and lithologically similar appearance.

The Miocene tuffs are mostly up to hundreds of meters-thick ignimbrites, originally emplaced over several thousand km^2^. Ignimbrite volcanism occurred in a back-arc setting belonging to the epicontinental Central Paratethys Sea^9,24^. The evolution of the sedimentary basin was determined by the rapidly changing extent and depth of the Paratethys^24,25^, controlled mostly by the interplay of eustasy and regional tectonics^22,25^. Consequently, well-defined marker horizons, e.g. pyroclastic units with regional distribution, may yield precise information on the time–space evolution of the basin, in particular the prevailing paleogeography^26^.

Within the basin, the pyroclastic units are best exposed in North Hungary, most widespread in the Bükk Foreland Volcanic Area (Fig. 1). There, the quarry of the Wind brick factory (Eger Northeast) is the type locality of the Upper Oligocene/Lower Miocene Egerian stage (corresponding to the Chattian-Aquitanian). Its geologic units reveal a shallow submarine sandstone/clay succession that overlays a 10 m-thick non-welded rhyolitic ignimbrite, which is the oldest of the regional pyroclastic stratigraphy^6^.

Seventy km northwest of Eger, at Ipolytarnóc, sedimentary deposits of the Central Paratethys are represented by marine ‘schlier’ (silty or sandy marl) and sandstone from the Eggenburgian/Ottnangian stages (roughly corresponding to the Aquitanian/Burdigalian). The sandstone reveals a diverse fossil fauna of shark teeth, molluscs, foraminifers, and corals^27^. It is overlain by clayey sandstone and conglomerate, informally called the “Ipolytarnóc Footprint Sandstone”, which exposes thousands of animal tracks (almost 3000 footprints from four predators, four birds, two even-toed ungulates and one rhinoceros species)^28^. The deposition of this sandstone marks the minimum age of the earliest arrival of Proboscidea (ancestors of today elephants) in Central Europe^13^. Paleogeographically, the sandstone facies characteristics (e.g., ripple marks, mouth delta layers) indicate a terrestrial habitat (“Rhinoland”)—likely a fluvial environment of braided rivers—interfingered with tide pools of a shallow epicontinental sea setting (“Crocodilia”)^28^. The spectacular tracks were preserved within oscillating, repetitive fluvial sequences of cm-thick footprint-bearing sandstone layers^28^.

The track site was discovered in the early nineteenth century where an exposed petrified giant tree trunk of *Pinuxylon tarnocziense* (originally up to 100 m tall), a relative to sugar pines, was found bridging two sides of a ravine^10,14,29^. Following the initial discovery, detailed analysis of the sandstone and the tuff revealed a number of tree trunks, branches, and more than 15,000 leaves. In all, seven conifers, four deciduous and one palm species were identified^30,31^, which indicated a multi-layer subtropical forest^31^. This habitat was hit by a large-scale volcanic eruption that knocked over and transported the tree logs a short distance, and eventually buried and preserved the track site with up to 40 m of tuff.

## Volcanic stratigraphy of the Eger-Ipolytarnóc eruption constrained by BSE imaging, pumice chemistry, Ar–Ar and U–Pb dating

In order to reconstruct the catastrophic volcanic event, we performed a detailed analysis of the volcanic deposit that buried Ipolytarnóc: its units, stratigraphic relations, and precise age. It belongs to the Lower Rhyolite Tuff (official formation name: Gyulakeszi Rhyolite Tuff), which is characterized by high-SiO_2_ (75–78 wt%) and high-K_2_O (> 4 wt%) rhyolitic glass^6,16,17^, and a mineral assemblage of quartz, plagioclase and biotite similar to many other pyroclastic deposits in the Pannonian Basin. Recently, detailed field-based volcanology studies of ca. 150 outcrops in North Hungary has facilitated an improved pyroclastic stratigraphy basin-wide^32,33^, including a robust correlation of the Lower Rhyolite Tuff.

### Volcanic succession

**Figure 2:**
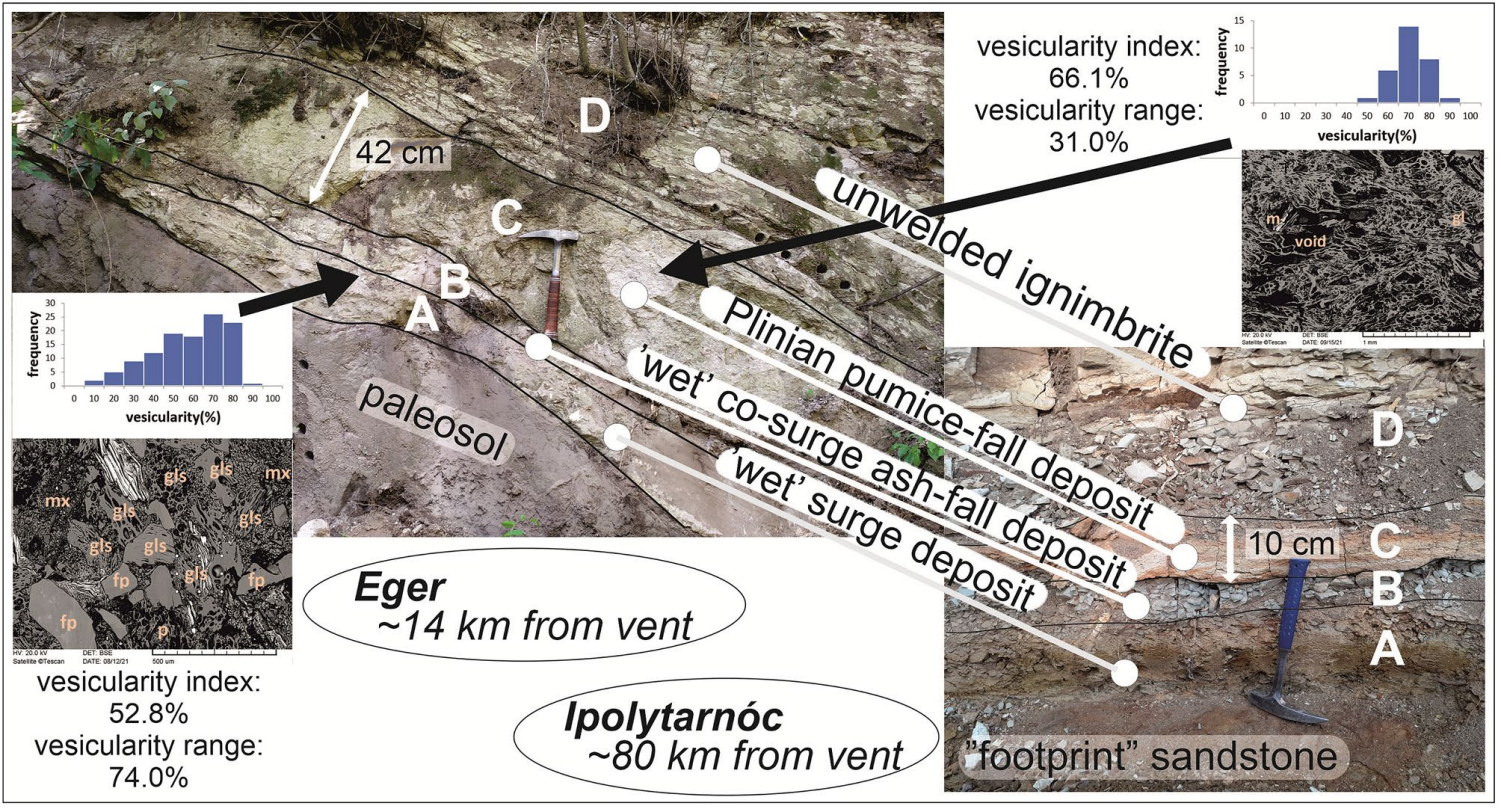
Correlation of two identical volcanic successions of the multi-phase Eger-Ipolytarnóc eruption showing macro- and microscopic features. On the left, the medial Eger Homok Street, on the right, the more distal Ipolytarnóc Fossil Site; both showing the correlated pyroclastic units (**A**-**D**). Insets display BSE images and vesicularity statistics (Supplement 1) for the first-phase (Unit A), low-temperature phreatomagmatic surge (bottom left), and the third-phase (Unit C) Plinian pumice fall (upper right). Photo credit: Dávid Karátson. Abbreviations in BSE images: mx—fine-grained matrix, fp—feldspar, gls—glass shard, m—mica, gl—glass, p—pumice. BSE (Backscattered Electron) images of selected thin sections were recorded using an AMRAY 1830 I/T6 Scanning Electron Microscope (Eötvös University).

Near the Wind Quarry, at Eger Northeast outskirts, we have found a complex outcrop in the deep cut of Homok Street exposing pyroclastic deposits (Fig. 2). Its base reveals the stratigraphically same ignimbrite (ca. 15 m thick) as the Wind Quarry top (hereafter called Wind Ignimbrite), followed by a weathering front passing upward to a paleosol. This in turn is overlain by four units (A-D) of a explosive silicic eruption which we call ‘Eger-Ipolytarnóc’ (Fig. 2). A detailed microscopic description of the four units with BSE imaging is given in Supplement 1.

The 20–25 cm-thick basal part is divided into two units: an undulating, laminated coarse tuff/lapilli tuff layer (Unit A) overlain by a fines-rich accretionary lapilli-bearing tuff (Unit B). Unit A contains sporadic but intact leaf fossils, of which the *Ulmus* genus and the *Cyclocarya cyclocarpa* species have been identified; both of them also occur in the Ipolytarnóc and the nearby Lipovany (Slovakia) flora^30^. The basal units in turn are draped by a 42 cm-thick coarse tuff (pumice fallout, Unit C) with pumice clasts up to 1 cm, passing upward to a few m-thick fine-grained lapilli tuff (ignimbrite, Unit D). Charred, mm- to cm-sized organic material (plant remains) are evenly distributed in its matrix. The same ignimbrite with ca. 20 m thickness and reverse grading of pumices is found at an abandoned quarry 0.3 km eastward (‘Eger Bányakert’), and in the lower yard of a double-level quarry 0.8 km southward (‘Eger Tuff Quarry’: Fig. 1) both with up to 20 cm-large pumice clasts toward top. The lower and upper ignimbrite of the latter quarry were denoted earlier as the Lower Lower and Upper Lower Tuff Complex^6^, or Eger and Mangó ignimbrite unit^16,32,33^, respectively. The total thickness of these two large ignimbrites cannot be seen at any individual locality, but both can reach 80 m based on the geometry of the covered relief.

**Figure 3:**
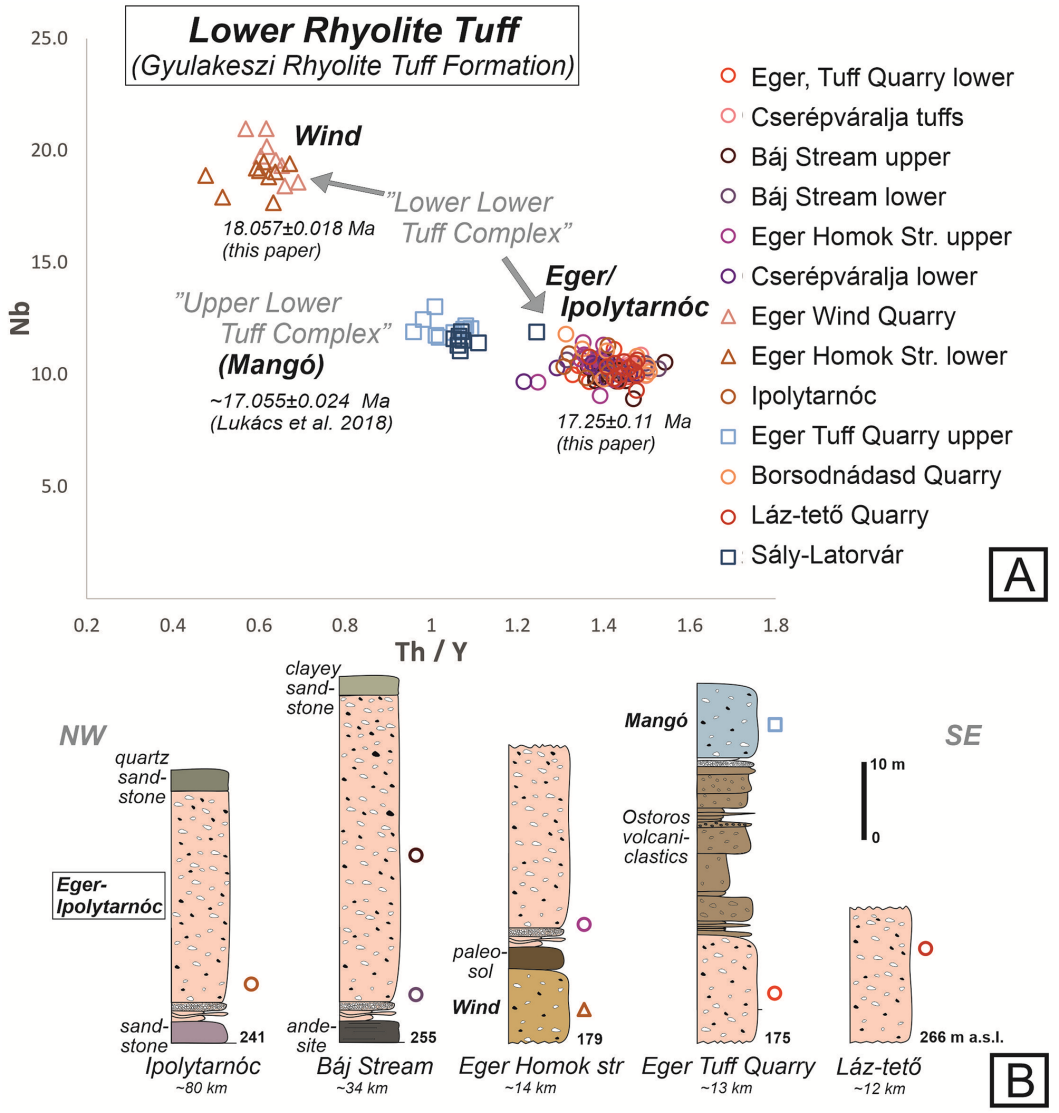
Correlation of units related to the initiation of the Miocene ignimbrite flare-up over the Northern Pannonian Basin. (**A**) Nb vs Th/Y plot of pumice glass of selected rhyolitic ignimbrite samples from North Hungary. For sample locations, see Fig. 1. Analytical data are provided in Supplement 2. (**B**) Simplified stratigraphic logs of study sites in the same area included in this study. The “Lower Lower Tuff Complex” consists of the oldest Wind Ignimbrite and the subsequent Eger-Ipolytarnóc Ignimbrite, followed by the “Upper Lower Tuff Complex” or Mangó Ignimbrite.

Twenty km west of Eger Homok Street, at the northern foot of Mátra Mountains, careful field logging of a known exposure of the Lower Tuff at Báj Stream^34^ revealed the same—although poorly preserved—basal succession as above (Fig. 3). There, the two basal units are overlain by a ~ 33 cm-thick pumice-fall deposit and then a fine-grained ignimbrite 60 m thick (pumice clast size ≤ 10 cm), intruded by a post-ignimbrite andesite sill.

Sixty-five km further to the northwest, at Ipolytarnóc, the ‘footprint’ sandstone is covered by a volcanic succession that we found again to be the same (Units A-D: Fig. 2). Unit A and B (of which the fines-rich upper layer with accretionary lapilli has a distinctive gray colour) and Unit C (with ≤ 5 mm pumice size) are divided by a sharp contact. The total thickness of Units A and B is roughly the same as in Eger. By contrast, the small (< 5 cm) pumice size and moderate thickness (10 cm) of the pumice fallout (Unit C) unambiguously indicates the systematic change of a Plinian pyroclastic-fall deposit toward distal exposures. The newly introduced name of Eger-Ipolytarnóc eruption is intended to highlight the correlation between the two furthest sites: a medial one (Eger Homok Str.) and a distal one (Ipolytarnóc).

To understand the development of successive phases of the Eger-Ipolytarnóc eruption in light of the main fragmentation processes, vesicularity (glass/void ratio of the pyroclasts) of selected tuff units (Units A and C) was determined via quantitative image analysis of BSE pictures (Supplement 1). Vesicularity index and vesicularity range were calculated following Houghton & Wilson^35^. The majority of the studied clasts are moderately to highly vesicular. Although there is some overlap, the fine-grained sample (Unit A) shows a generally broader range of vesicularity than the coarse-grained sample (Unit C). However, the fine-grained Unit A sample also contains poorly vesicular clasts, represented by small-sized ash plates and ash flakes. These finds are interpreted in the Discussion.

In order to confirm the correlation of individual eruptive units established by field mapping, geochemical analysis of the major and trace element composition of pumice glass from 13 ignimbrite outcrops were conducted by using electron microprobe analysis (EMPA) and laser ablation inductively coupled mass spectrometry (LA-ICP-MS) techniques (Supplement 2). All analysed pumice glass fragments show similar rhyolitic composition; however, the concentration of trace elements define three groups as illustrated e.g. by the Nb vs Th/Y plot (Fig. 3). Discrimination between these groups fully support the correlation of the three ignimbrites from base to top: the oldest Wind, the Eger-Ipolytarnóc, and the Mangó Ignimbrite.

### Ar–Ar and U–Pb dating

For the Eger-Ipolytarnóc Ignimbrite, U–Pb dating^16,17^ and combination of U–Pb and Ar–Ar dating were previously applied^13,36^, but the results did not clarify the precise eruption age (Table 1). In our study, both dating methods were applied to the Wind and Eger-Ipolytarnóc ignimbrites (Table 1) to compare these methods and to check the consistency of obtained ages with field relations.

**Table 1:** Ar–Ar and U–Pb ages from the Wind and the Eger-Ipolytarnóc ignimbrites.

Ignimbrite	Locality	U–Pb zircon	Ar–Ar plagioclase	Ar–Ar sanidine	Ar–Ar biotite
Eger- Ipolytarnóc	Ipolytarnóc	17.42 ± 0.04^13^	17.13 ± 0.14^13^**		
		*17.45 ± 0.19*	**17.25 ± 0.11**		
		17.2 ± 0.3, 17.3 ± 0.3, 17.6 ± 0.3^17^			
	Lipovany		17.49 ± 0.54^36^		17.28 ± 0.06^36^
	Mučin	17.4 ± 0.3^17^			
	Eger (Homok Str. upper)	*17.49 ± 0.20*			
	Eger (Tuff Quarry)	17.5 ± 0.3^16^*			
	Báj Stream (upper)	*17.67 ± 0.20*			
	Láz-tető	*17.71 ± 0.21*			
	Báj Stream (lower)	*17.78 ± 0.20*			
Wind	Cserépváralja (CSV-2 borehole)	18.2 ± 0.3^16^			
	Eger (Homok Str. lower)	*18.21 ± 0.19*	*18.21 ± 0.08*	**18.057 ± 0.018**	

Values in italics are determined in this paper, the other ones are taken from literature. Uncertainties are given in 2σ.

*Average of 2 datings.

**Corrected from 17.02 ± 0.14 Ma using the revised Fish Canyon Tuff age (^51^Kuiper et al. 2008).

Preferred eruption ages are in bold.

Zircon U–Pb dating (Supplement 3) was performed in the Laboratoire Magmas et Volcans, Clermont-Ferrand (France). The Wind Ignimbrite yielded an age of 18.21 ± 0.19 Ma (2σ), whereas obtained ages of the Eger-Ipolytarnóc Ignimbrite range between 17.45 and 17.78 Ma.

^40^Ar–^39^Ar dating (Supplement 4) was performed at New Mexico Geochronological Research Laboratory, Socorro (USA). Single-crystal sanidine from the Wind Ignimbrite yielded a high-precision weighted mean age of 18.057 ± 0.018 Ma (2σ), and a plagioclase isochron age of 18.21 ± 0.08 Ma (2σ). For the Eger-Ipolytarnóc Ignimbrite, which does not contain sanidine, single plagioclase crystals were step-heated with two steps each. About twelve of the twenty-three spectra revealed plateau ages. The other spectra showed a discordant pattern where the initial low-temperature step (A) is mostly older and poorly constrained, and the second high-temperature step (B) is younger and better constrained. Taking into account only the more precise and less contaminated high-temperature steps, an isochron age of 17.25 ± 0.11 Ma (2σ) was calculated.

## Discussion: timing, succession, and paleo-environmental effects of the Eger-Ipolytarnóc eruption

Several of the well-known Late Pleistocene high-end VEI = 7 or VEI = 8 large-magnitude eruptions (e.g. Toba^37^, Campanian^38^, Ōruanui^39^) with hundreds of km^3^ tephra volume^40,41^ and, typically, caldera formation^42^, had disrupting impact on the local ecosystem and even the global climate^40,43^. While subsequent to these young eruptions the massive tephra cover has buried the paleotopography, at ancient settings the millions of years of erosion can bring buried habitats to the surface. This is the case, for example, with the Permian Chemnitz petrified forest in Germany^44^, the Cretaceous Jihol biota in Liaoning Province, China^45^, or the Miocene Ashfall Fossil Beds, Nebraska, USA^1,46^. Our study on Ipolytarnóc adds a poorly preserved but still reconstructible large Early Miocene explosive silicic eruption that preserves an important paleohabitat to the global picture.

The first eruption in the Northern Pannonian Basin produced the Wind Ignimbrite. Its burial by successive ignimbrites have resulted in it being found on the surface only at the Wind Quarry; elsewhere it has been identified from boreholes^16^. Following this ignimbrite, by correlating medial and distal sites, we demonstrate that recurrent activity produced the geochemically different, much larger Eger-Ipolytarnóc Ignimbrite (Fig. 3).

As for the precise dating of these first two eruptions of the Northern Pannonian Basin, both the ^40^Ar–^39^Ar and U–Pb methods were applied. In general, it is well accepted that the ^40^Ar–^39^Ar method will provide precise eruption ages. Ideally, this should be conducted on a high-K phase like sanidine, owing to its generally simple argon systematics (minimal argon loss or excess argon, and high radiogenic yield)^47^. As presented above, the Wind Ignimbrite sanidine has been precisely dated here at 18.06 ± 0.02 Ma, and this can be taken as the eruption age. However, as the subsequent Eger-Ipolytarnóc Ignimbrite does not contain sanidine, it was dated using plagioclase.

### Age of the eruption

If we take into account all heating (A and B) steps of ^40^Ar–^39^Ar dating, the Eger-Ipolytarnóc eruption yields more scattered data and a less constrained isochron age of 17.17 ± 0.18 Ma (Supplement 4). So, no matter the combination of the data, it yields an age that within uncertainty matches the preferred eruption age of 17.25 ± 0.11 Ma (i.e. high-temperature steps only). The relatively high trapped initial ^40^Ar/^36^Ar ratio of the plagioclase isochron suggests that a sanidine age would be slightly younger. This way, the ~ 17.2 Ma plagioclase age may indicate an upper limit for the eruption age.

Zircon U–Pb ages obtained in this study, as well as those from previous work^13,16,17^, show a significant scatter and even the younger values are consistently ca. 0.2 My older than the ^40^Ar–^39^Ar ages (Table 1). Due to magma residence time issues^,^^48–50^, this difference may reflect the long crystallization history recorded by the zircons.

Notably, within 2σ uncertainty, the ^40^Ar–^39^Ar age obtained for the Eger-Ipolytarnóc Ignimbrite age is coeval with either the Ar–Ar plagioclase date of Pálfy et al.^13^ once corrected (17.13 ± 0.14 Ma) for the revised Fish Canyon Tuff age^51^, or the Ar–Ar biotite age (17.28 ± 0.06 Ma) of Šarinová et al.^36^ (Table 1). Thus we suggest that our plagioclase ^40^Ar–^39^Ar age records a robust eruption age of the Eger-Ipolytarnóc Ignimbrite. The ~ 17.2 Ma age is on the Ottnangian/Karpatian boundary of the Central Paratethys stratigraphy^25^ and, at the same time, on the boundary of the C5D and C5C magnetic polarity zone. The eruption was soon followed by another explosive eruption of similar magnitude, the Mangó Ignimbrite^6,33^ (17.055 ± 0.024 Ma based on ID-TIMS zircon U–Pb dating^16^).

### Magnitude of the eruption

**Figure 4:**
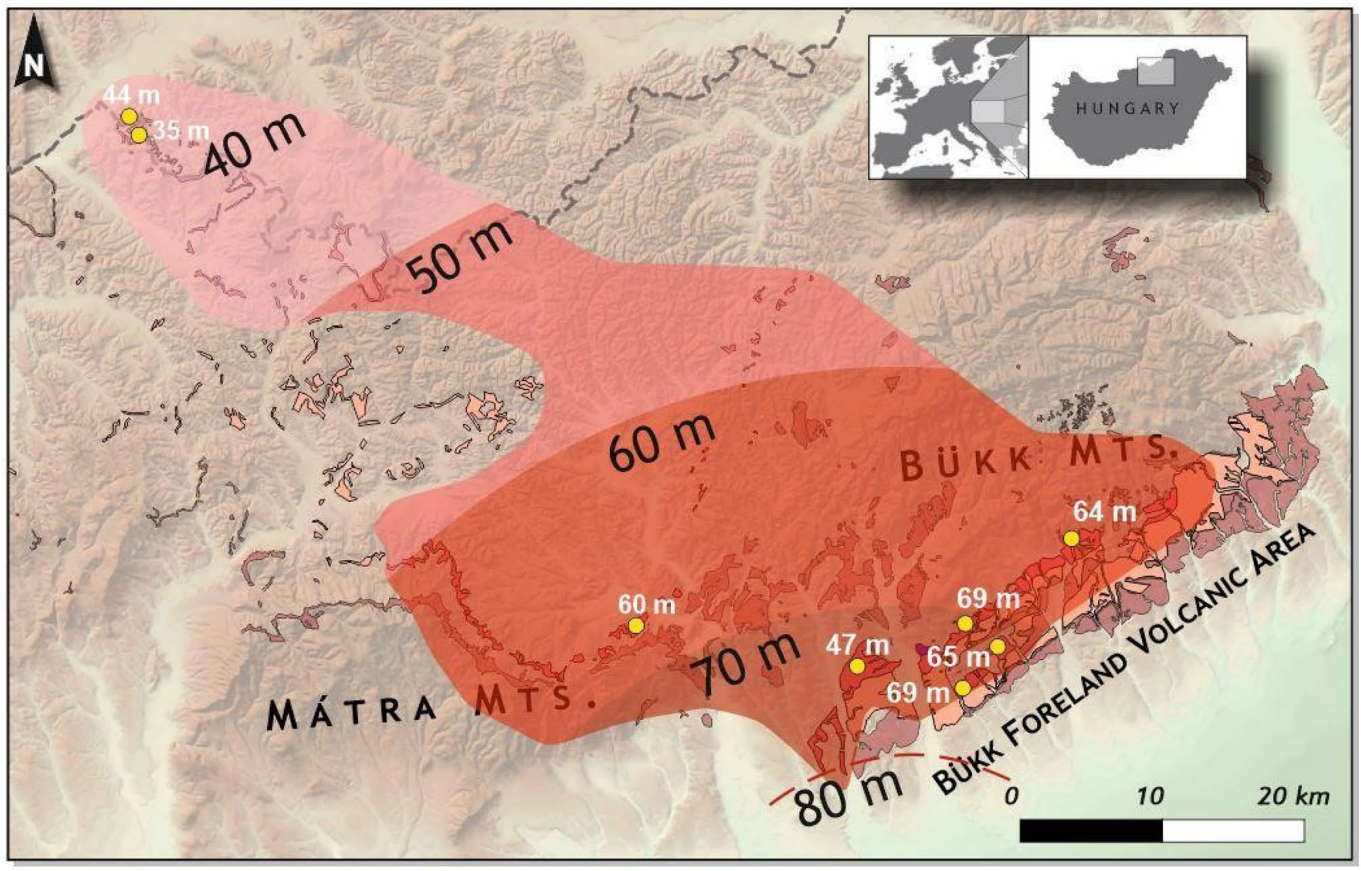
Volumetry of the Eger-Ipolytarnóc Ignimbrite. Based on the scattered surface occurrences, the extrapolated area of the pyroclastic deposits belonging to the Eger-Ipolytarnóc eruption (draped over the shaded relief image of 50 m DEM of Hungary) currently covers ~ 1650 km^2^. (Image created with Surfer 13, version 13.0.383 Golden Software). The neotectonic subsidence of the area south of the Bükk Foreland^22^ (including the probable source area: Fig. 1B, and the Great Hungarian Plain further southward) has left only a part of the pyroclastic deposits, ranging from medial to distal, on the surface. However, for some occurrences (yellow dots), total ignimbrite thickness can be observed or inferred. On this basis, assuming uniform thinning to the distal Ipolytarnóc locality (with an observed thickness of 40 m; extrapolated thickness values indicated), the outlined area defines a bulk tephra volume of c. 99 km^3^, which may correspond to c. 58 km^3^ DRE (original magma volume), and points to a high-end VEI = 7 eruption (for details of calculation, see Supplement 5). Obviously, the present-day distribution is confined within a disjointed sector; original dimensions were probably much larger. Whereas pyroclastic deposits removed by erosion cannot be taken into account reliably, those subsided in the Pannonian Basin can be possibly constrained via borehole data.

Based on the large areal distribution of the Eger-Ipolytarnóc Ignimbrite (Fig. 1), it affected the regional landscape of the whole Northern Pannonian Basin (Fig. 1). A negative gravity anomaly south-southeast of Eger town, proposed already as a potential vent area^6^ (Fig. 1), defines the distance from source: ~ 14 km for Eger and ~ 80 km for Ipolytarnóc. Distribution of the known or newly identified present-day surface occurrences, and their observed and interpolated thickness data (Fig. 4), define an area of ~ 1650 km^2^ and a bulk volume of 99 km^3^, respectively (Supplement 5). This volume, if calculated with a 1.7 bulk rock/DRE (dry rock equivalent) ratio, corresponds to 58 km^3^ magma. However, due to the very poor preservation of ignimbrites, these values should be considered as minimum/conservative estimates, and the eruption may have been several times larger. Yet, even the calculated minimum values exceed the VEI = 7 Taupō AD 232 eruption in New Zealand (105 km^3^ bulk, 30 km^3^ DRE)^52^ and are close to the high-end VEI = 7 Late Bronze Age Minoan eruption of Santorini (123 km^3^ bulk, 82 km^3^ DRE)^53^ and the 7700 ka Mt. Mazama (Crater Lake) eruption in Oregon, USA (176 km^3^ bulk, 61 km^3^ DRE)^54^.

### Evidence for a ‘wet’ eruption

Analysis of the pyroclastic sequence of this large-magnitude explosive eruption shows that its first two phases were a double event. As BSE imaging reveals (Fig. 2 and Supplement 1), vesicle texture and vesicle area fraction of the studied pumice clasts both in Unit A and C are fairly similar, and indicates a similar degassing history to silicic Plinian explosive eruptions^55^. However, since Unit A contains abundant very fine juvenile glass shards, its fragmentation was more effective than Unit C. The broad range of vesicularity in Unit A indicates an additional effect during fragmentation that is beyond vesicle bursting. We suggest that ‘wet’ fragmentation due to already vesiculated magma and external water interaction in the conduit at a specific ratio of the two components^56^ was responsible for producing this fine-grained, laminated layer, that we interpret as being deposited from a subcritical pyroclastic density current (PDC), namely a highly energetic, dilute phreatomagmatic surge^57–59^. The wet character of this phase became even more enhanced during the deposition of Unit B considering the abundance of accretionary lapilli. Units A and B are collectively interpreted as a “couplet” in which the phreatomagmatic PDC (surge) was associated with a co-PDC ash fall^60^. As no abundant lithics were found in the basal units, we suggest that shallow water was the main source of external water supply.

Highly vesicular pumice clasts in Unit C (Fig. 2) are inferred to have formed by purely magmatic, ‘dry’ fragmentation due to the decompression-driven degassing and related expansion of the non-permeable magma (Fig. 2 and Supplement 1) to produce a Plinian pumice fall. During this phase, which may have followed after a pause, access to (shallow) water might have been suspended. Thickness values of the pumice-fall deposit and their systematic decrease from southeast to northwest (Figs. 1, 2 and 3: Eger 42 cm, Báj stream 33 cm, Ipolytarnóc 10 cm) suggests that Eger Homok Street was located medially, whereas Ipolytarnóc distally relative to the source.

Finally, Unit D corresponds to a typical pumiceous pyroclastic flow emplacing a large-volume ignimbrite. During this phase, limited access to water should have re-appeared, as indicated by (a) the total absence of welding in the deposit (even at sites relatively closest to vent: Láz-tető Quarry, Eger ‘Bányakert’ Quarry, Fig. 1) and (b) the presence of charred organic remains medially (e.g. Eger Homok Street) and all places beyond.

**Figure 5:**
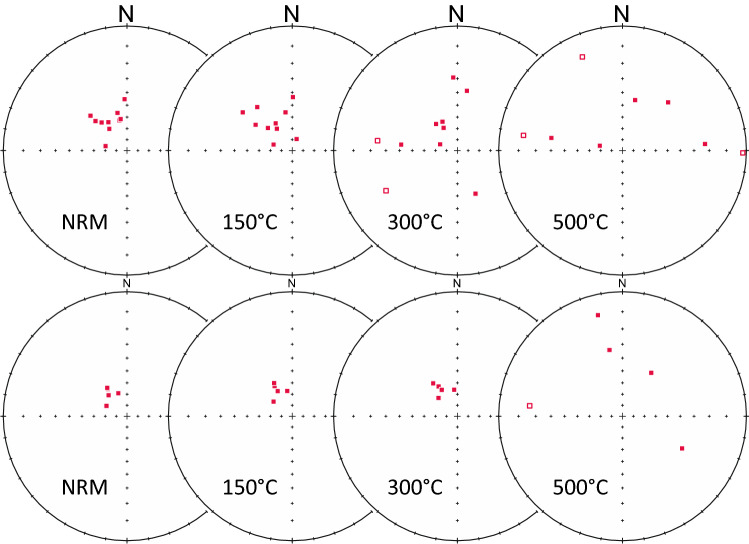
Examples of the loss of well-defined paleomagnetic signal (NRM: individual directions in the natural state) in the “cold” Eger-Ipolytarnóc ignimbrite, as documented by the increasing scatter of the directions from 150 °C in the ignimbrite immediately above the ‘footprint’ sandstone (upper row) and after 300 °C in the massive ignimbrite of the Borókás Ditch (lower row), both at Ipolytarnóc. Normal (full symbols) and reversed (empty symbols) polarity directions are plotted in stereographic projections. Analyses performed on samples from Márton et al. (2007)^12^.

Effect of water on temperature can also be assessed by analysing paleomagnetic properties. A large number of paleomagnetic measurements are available from the Miocene tuffs of the Salgótarján Basin and the Bükk Foreland Volcanic Area (Fig. 1). Several of them are related to the Ipolytarnóc area, including some outcrops beyond the Slovakian border^12^. At Ipolytarnóc, a number of ignimbrite samples drilled in different horizons provided well-grouped paleomagnetic directions in the natural state^12^. The cluster remained stable during stepwise alternating field demagnetization till the complete loss of the paleomagnetic signal. When stepwise thermal demagnetization was applied to the same horizons containing magnetite as the carrier of the remanence, the paleomagnetic signal was lost, well before the Curie point of the magnetite (Fig. 5). This finding is consistent with worldwide examples of ‘cold’ ignimbrite emplacement lacking high-temperature natural remanent magnetization (NRM)^61,62^. Furthermore, we can see a temperature drop towards distal settings as testified by the appearance of locally incorporated, uncharred tree trunks and leaves at Ipolytarnóc, which is similar to, for instance, the Peperino Albano Ignimbrite (Colli Albani volcano, Italy)^61^. At Ipolytarnóc, the presence of uncharred plant remains indicates a maximum temperature of 150 °C^63^.

### Implications for paleogeography and geosphere-biosphere interactions

As for paleogeographic implications, the pyroclastic succession was deposited in a terrestrial environment both in Eger and Ipolytarnóc, 70 km apart, which points to a period of marine regression during the Central Paratethys evolution. We argued that Unit A and Unit B which show phreatomagmatic character suggest an accessible water supply, but it must have been confined (e.g. to a caldera lake, shallow lacustrine environment, or limited groundwater).

To understand the scenario of the natural catastrophe at Ipolytarnóc, let us recall the onset of the eruption, when the area—as shown above—was characterized by a peculiar habitat of a fluvial environment interfingered with shallow-marine tide pools. Based on the richness of fossil tracks, this environment was populated by a wide variety of animals. In order to get an idea what a momentary event it may have been, the Taupō AD 232 eruption, similar in magnitude, can serve as an extreme analogy: its pyroclastic flows were inferred to have had a velocity of 150 m/s and, with a similar radius (80 km runout distance) to the Eger-Ipolytarnóc Ignimbrite (Fig. 1), covered an area of 20,000 km^2^ in less than 10 min^52^. This implies that, although the first, double eruption event at Ipolytarnóc deposited only thin layers, it was a highly energetic PDC which hit the paleoenvironment catastrophically.

However, based on the analysis of the successive pyroclastic layers, the two initial phases were ‘wet’ phreatomagmatic events. This means that, in contrast to previous views, the Eger-Ipolytarnóc eruption was not similar to ‘Pompeii’—i.e., the AD 79 Plinian eruption of Somma-Vesuvius—where the lethal events for living organisms were 200–500 °C hot PDCs^64–66^ (following several hours of pumice fall). It is also different from the Taupō AD 232 eruption, where charred tree trunks indicate 270–400 °C^67^. At Ipolytarnóc, in agreement with the inferred low temperature of the first phreatomagmatic phases, logs remained uncharred, there are plenty of leafs, whereas animal bones or remains killed by hot gas are neither expected nor found—contrary to, for instance, the above-mentioned Ashfall Fossil Beds, which reveals a mass-death assemblage of a diverse fauna under 3-m-thick ultra-distal ash-fall deposit^1^.

### Concluding remarks

**Figure 6:**
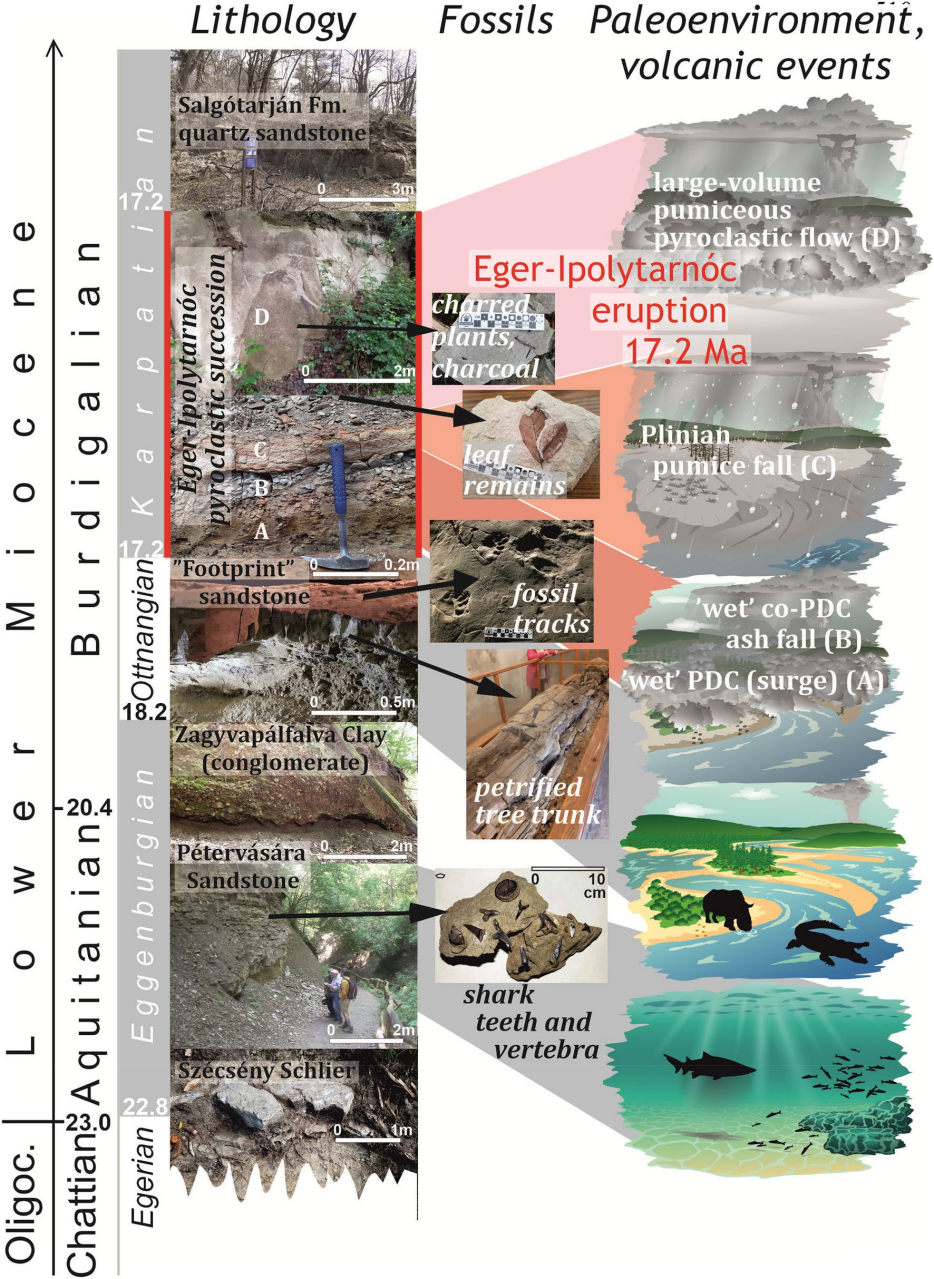
Paleoenvironments and proposed volcanic succession of Ipolytarnóc Fossil Site in the context of Central Paratethys stratigraphy. Geologic time scale in My.

In summary, the successive events of the Eger-Ipolytarnóc eruption (Fig. 6) indicate that at first the low-temperature but high-energy PDC (surge) disrupted the ecosystem over a large area in a few tens of minutes at most. At Ipolytarnóc, the surge event toppled the trees and covered the habitat with thin tuff. Based on the identified low temperature, we suggest that the cold deposition may have been a crucial factor in allowing the animals to escape. In fact, the absence of animal remains, juxtaposed with thousands of footprints, indicate a pause after the first, small-scale events. After this pause of unknown length, a Plinian pumice fall and, eventually, a low-temperature, high-volume pumiceous pyroclastic flow followed—the ignimbrite that was deposited from the latter represents the overwhelming majority of the eruptive volume –, conserving the buried floral elements and the footprints. Studying such low-temperature pyroclastic successions may be of paramount importance for paleontology worldwide, since eruptions of this kind can ensure the preservation of the biota.

## Supplementary Information

Supplementary Information 1A.

Supplementary Information 1B.

Supplementary Information 1C.

Supplementary Information 2A.

Supplementary Information 2B.

Supplementary Information 3A.

Supplementary Information 3B.

Supplementary Information 4A.

Supplementary Information 4B.

Supplementary Information 4C.

Supplementary Information 4D.

Supplementary Information 5.

Supplementary Information Summary.

## Data Availability

All data generated or analysed during this study are included in this published article (and its Supplementary Information files).
