# Supplementary material for: Large-magnitude (VEI ≥ 7) ‘wet’ explosive silicic eruption preserved a Lower Miocene habitat at the Ipolytarnóc Fossil Site, North Hungary
Source: Sci Rep. 2022 Jun 13;12:9743. doi: 10.1038/s41598-022-13586-3 (PMC9192734; doi:10.1038/s41598-022-13586-3)
Supplement: Supplementary file 3 — Supplementary Information 1C. [file 41598_2022_13586_MOESM3_ESM.pdf]

Pyroclast vesicularity of the Eger - Ipolytarnóc eruption

Methods

Bulk samples were taken from every unit of the studied outcrop at Eger Homok Street (upper part). The samples were cut into half and impregnated with synthetic resin which resulted in ca. 5-20 cm^2 surfaces. Each sample surface after the cut was polished. Petrographic thin sections were also prepared from selected samples in which the resin could not properly infiltrate.

BSE (Backscattered Electron) images of selected thin sections were recorded using an AMRAY 1830 I/T6 Scanning Electron Microscope of the Department of Petrology and Geochemistry, Eötvös Loránd University (Budapest, Hungary). For image acquisition the SEM was set to 20 kV and 5 nA.

Chemistry of the pumice glass and the glass shards (Supplement 1, Table I) were measured using the EDX (PU9800) detector of the AMRAY SEM, applying 20 kV and 1 nA. The Yellowstone rhyolite and Hawaiian basaltic glass standards of the Smithsonian Institute (Jarosewich, 2002) were used for calibration.

Obtained BSE images were used for vesicularity analyses applying the nested image technique following Klug and Cashman (1994) and Shea et al. (2010). The BSE images were processed with the FIJI- ImageJ (Schneider et al. 2012) open-source image analyses software to create binary images using the built-in auto thresholding function; when it was necessary the automatic results have been manually refined.

The 2D (two dimensional) area fraction of the glass have been measured (Suplement 1, Table II). Klug and Cashman (1994) suggested that the 2D area fraction of the vesicles equals the volume fraction in three dimension and yields clast vesicularity in case of random vesicle orientation. The vesicularity index which represents the mean value of the measured vesicularity and the vesicularity range which represents the total spread of the measured values were calculated following Houghton and Wilson (1989).

Results

Petrography and glass chemistry

Unit A shows two subfacies. Unit A_1 subfacies is a pale greyish yellow fine-grained tuff. The tuff is matrix supported with 5% crystals and rounded white micropumice clasts. The crystals are quartz, feldspar, and dark mica (Supplement 1, Fig. 1A). Unit A_2 subfacies is a whitish grey, layered, coarse-grained tuff (Supplement 1, Fig. 1B).The matrix supported tuff contains rounded, white pumice clasts and quartz, feldspar, and dark mica crystals (Supplement 1, Fig. 2A). Unit B is a dark brown fine-grained tuff containing mm sized accretionary lapilli concentrating at the base of the unit (Supplement 1, Fig. 1C). The accretionary lapilli have a well-defined core and

rim (Supplement 1, Fig. 2B). This unit has diffused transition and flame structure at the base. Unit C consists of whitish grey pumiceous lapillistone (Supplement 1, Fig.1 D, E). Quartz, feldspar, and dark mica are present as phenocrysts. The pumices are angular and oriented. Unit D is a gray lapilli tuff with high number of phytogenic clasts (Supplement 1, Fig. 1F). Quartz, feldspar, and dark mica were observed as loose crystals in the matrix (Supplement 1, Fig. 2D).

Supplement 1, Table I contains the glass chemistry results measured with the EDX detector of the AMRAY electron microscope. The measured glass composition was used only for relative comparison of Unit A and Unit C. The SiO_2/Al_2O_3 ratio of Unit A is slightly higher compared to Unit C, but this difference is negligible. SiO_2/Al_2O_3 vs Na_2O/K_2O ratios of the glass indicate homogenous major element melt geochemistry for these units.

Vesicularity

BSE image analysis was effective in characterizing Unit A and Unit C. Samples from these units contained appropriate pumice clasts for vesicularity analyses. The vesicles of the pumices from these samples were studied to understand the main conduit processes as degassing and fragmentation during the eruption.

Most of the studied clasts of Unit A and Unit C are highly and moderately vesicular (Supplement 1, Table II and Supplement 1, Fig. 4B) according to the Houghton and Wilson (1989) classification. The Unit A sample also contains poorly vesicular clasts population. The larger vesicularity range can indicate heterogenous and mature, partly degassed conduit at the time of fragmentation (e.g. Cashman 2004). However, the size dependent vesicularity analyses of Unit A (Supplement 1 Fig. 4A) indicates logarithmic correlation between clast size and vesicularity, in other words the poorly vesicular clasts are only represented by small sized platy and flaky ash while the larger pumice clasts (> 500 μm) are highly-moderately vesicular similar to the pumices of Unit C. Based on Walker (1980) and Houghton and Wilson (1989), the vesicularity of the clasts increases as the size of the clasts converges to the diameter of the vesicles. Therefore, the broad range of vesicularity in Unit A, especially the poor vesicularity, is only apparent, and the poorly vesicular clasts are interpreted as testifying the strongly fragmented material of moderately/highly vesicular magma. This also suggests that the pre-fragmentation vesicularity of Unit A and Unit C magma was similar, indicating comparable decompression history for both units, but with a more effective fragmentation in the case of Unit A. We propose that similarly to the Askja 1875 (Carey 2009) or Grímsvötn 2011 (Liu et al. 2015) eruptions, in the case of Unit A the already vesiculated expanding magma fragmented more efficiently forced by the explosive magma-water interaction.

Phreatomagmatic fragmentation occurs due to magma and water interaction in the conduit. The involvement of water during the fragmentation produces fine-grained deposit in contrast to the magmatic volatile-driven, dry fragmentation (e.g., Wolhetz 1986, Austin-Erickson et al. 2008, Németh & Kósik 2020). The lower vesicularity index and higher vesicularity range in Unit A tuff indicates magma-water interaction during the early stages of the Ipolytarnóc eruption. The involvement of water is also supported by the high amount of fine ash in Unit A and abundant presence of accretionary lapilli in Unit B (see Supplement 1 Fig. 2B), which is probably a co-

PDC plume product deposited on the top of Unit A PDC (Pyroclastic Density Current) deposit (Schumacher & Schminke, 1995). The relative abundance of highly vesicular clasts in Unit A suggests late-stage, explosive magma-water interaction of the already degassed expanding magma which was near to or probably just above its fragmentation threshold. It shall be noticed that in contrast to Unit A and B, Unit C vesicularity distribution and two-dimensional vesicle textures (Supplement 1 Fig 3. A-E) indicates dry fragmentation and falls into the range measured for large Plinian eruptions (Cashman 2004). As field observations suggest, Unit C deposited directly on the top of Unit B with sharp boundary, without any signs of intereruptive erosion indicating lack of longer quiescence (Supplement 1 Fig. 2 of main text). Thus, during the Eger-Ipolytarnóc eruption the initial phreatomagmatic phase (Unit A, B) has been followed by a dry magmatic phase represented by Unit C fallout deposit. The transition between these phases was sharp. The sharp transition between wet, phreatomagmatic, and dry magmatic fragmentation mode can be interpreted as a result of a) the depletion of available water supply (e.g., caldera lake) or b) vent position shifting similar to the eruptions of Askja in 1875 or Taupo in 232 (Carey et al. 2009, Houghton et al 2010).

References

Austin-Erickson, A., Büttner, R., Dellino, P., Ort, M. H., & Zimanowski, B. (2008). Phreatomagmatic explosions of rhyolitic magma: experimental and field evidence. *J. Geophys. Res. Solid Earth,* **113 (B11)**.

Carey, R. J., Houghton, B. F., Thordarson, T. (2009): Abrupt shifts between wet and dry phases of the 1875 eruption of Askja Volcano: Microscopic evidence for macroscopic dynamics. *J. Volcanol. Geotherm. Res.,* **184 (3-4)**, 256-270.

Cashman, K. V. (2004): Volatile controls on magma ascent and eruption. *The State of the Planet: Frontiers and Challenges in Geophysics,* **150,** 109-124.

Houghton, B. F. et al. (2010): Diverse patterns of ascent, degassing, and eruption of rhyolite magma during the 1.8 ka Taupo eruption, New Zealand: evidence from clast vesicularity. *J. Volcanol. Geotherm. Res.,* **195 (1),** 31-47.

Houghton, B. F. & Wilson, C. J. N. (1989): A vesicularity index for pyroclastic deposits. *Bull. Volcanol.* **51 (6),** 451-462.

Jarosewich, E. (2002): Smithsonian microbeam standards. *J. Res. Nat. Inst. Standards and Technology*, **107 (6),** 681.

Klug, C. & Cashman, K. V. (1994): Vesiculation of May 18, 1980, Mount St. Helens magma. *Geology,* **22 (5),** 468-472.

Liu, E. J., Cashman, K. V., Rust, A. C., & Gislason, S. R. (2015). The role of bubbles in generating fine ash during hydromagmatic eruptions. *Geology,* **43 (3),** 239-242.

Németh, K. & Kósik, Sz. (2020): Review of explosive hydrovolcanism. *Geosciences,* **10 (2),** 44.

Schumacher, R, Schmincke, H.-U. (1995): Models for the origin of accretionary lapilli. *Bull. Volcanol.,* **56 (8),** 626-639.

Shea, T., Houghton, B. F., Gurioli, L., Cashman, K. V., Hammer, J. E., & Hobden, B. J. (2010): Textural studies of vesicles in volcanic rocks: an integrated methodology. *J. Volcanol. Geotherm. Res.,* **190 (3-4),** 271-289.

Walker, G. P. L. (1980) The Taupo pumice: product of the most powerful known (ultraplinian) eruption? *J. Volcanol. Geotherm. Res.,* **8 (1),** 69-94.

Wohletz, K. H. (1986) Explosive magma-water interactions: Thermodynamics, explosion mechanisms,and field studies. *Bull. Volcanol.* **48 (5),** 245-264.

Supplement 1 figures:

- Figure 1 A-F: polished cut surface of representative samples from Unit A-D, collected at Eger Homok Street and Ipolytarnóc.
- Figure 2 A-D: BSE images showing generic microscopic view of Unit A-D from Eger Homok Street
- Figure 3 A-E: Binary images of pumice textures from Unit C sample of Eger Homok Street.
- Figure 4 A-B: Vesicularity diagrams of Unit A and Unit C.

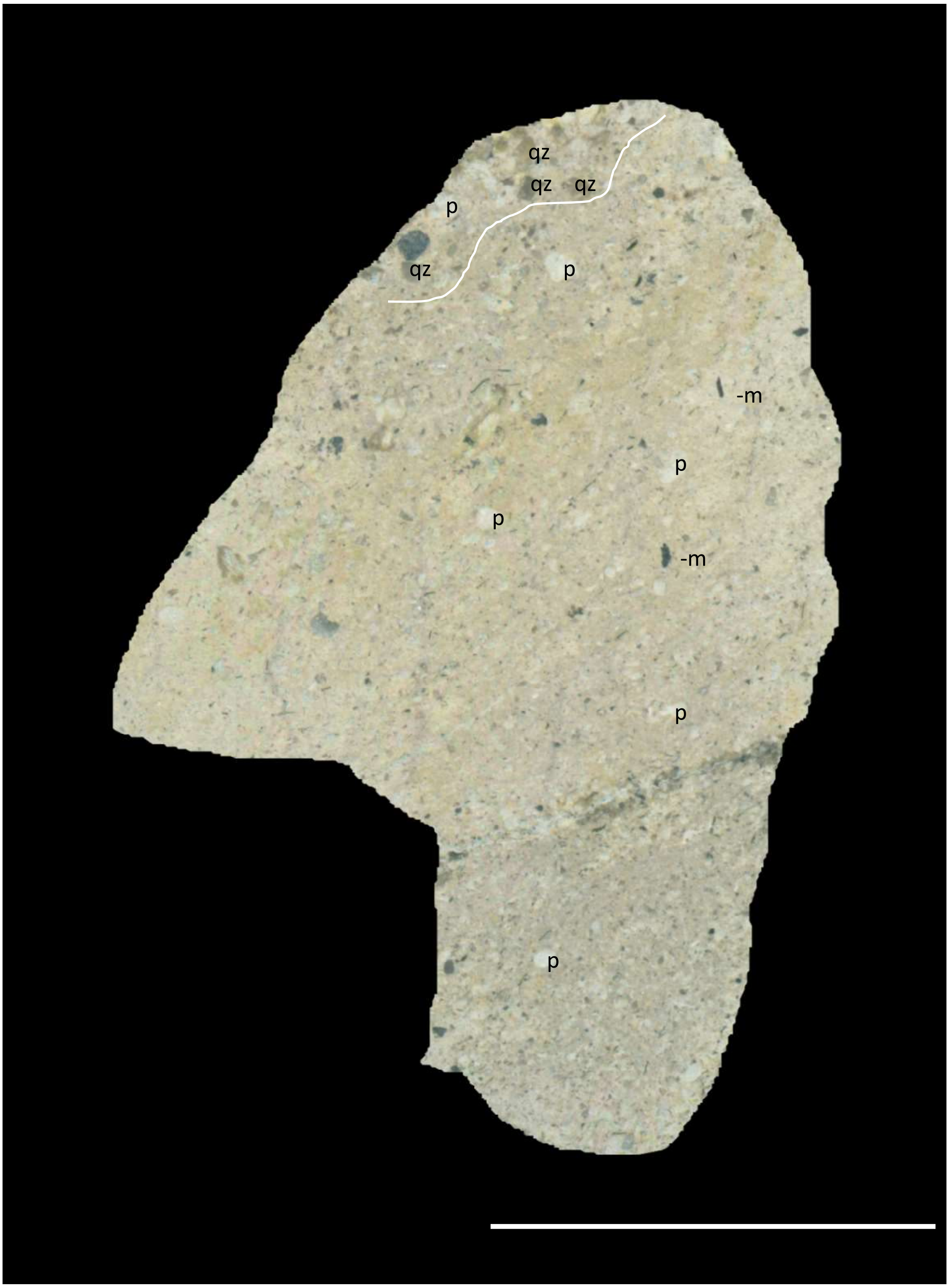

Figure 1A: Unit A - Polished cut surface of the fine tuff facies of the basal laminated unit of the Eger-Ipolytarnóc pyroclastic sequence. The tuff contains pumice clasts and a crystal accumulation zone at the top. P = pumice, qz = quartz, m = mica. Scale is 2 cm.

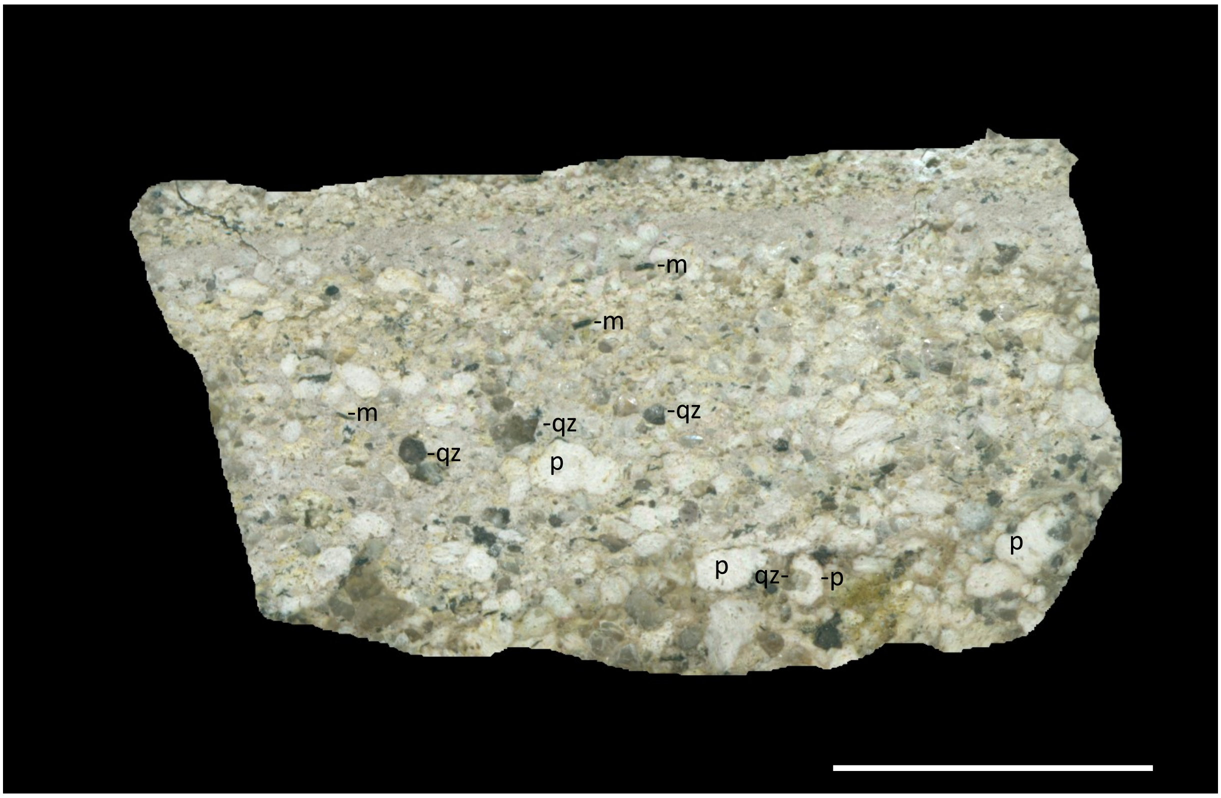

Figure 1B: Unit A - Polished cut surface of the coarse-grained laminated tuff facies of Unit A. This facies is characterized by the accumulation of coarse-grained pumice clasts and loose crystals in a fine grained matrix and shows normal grading. P = pumice, qz = quartz, m = mica. Scale is 2 cm.

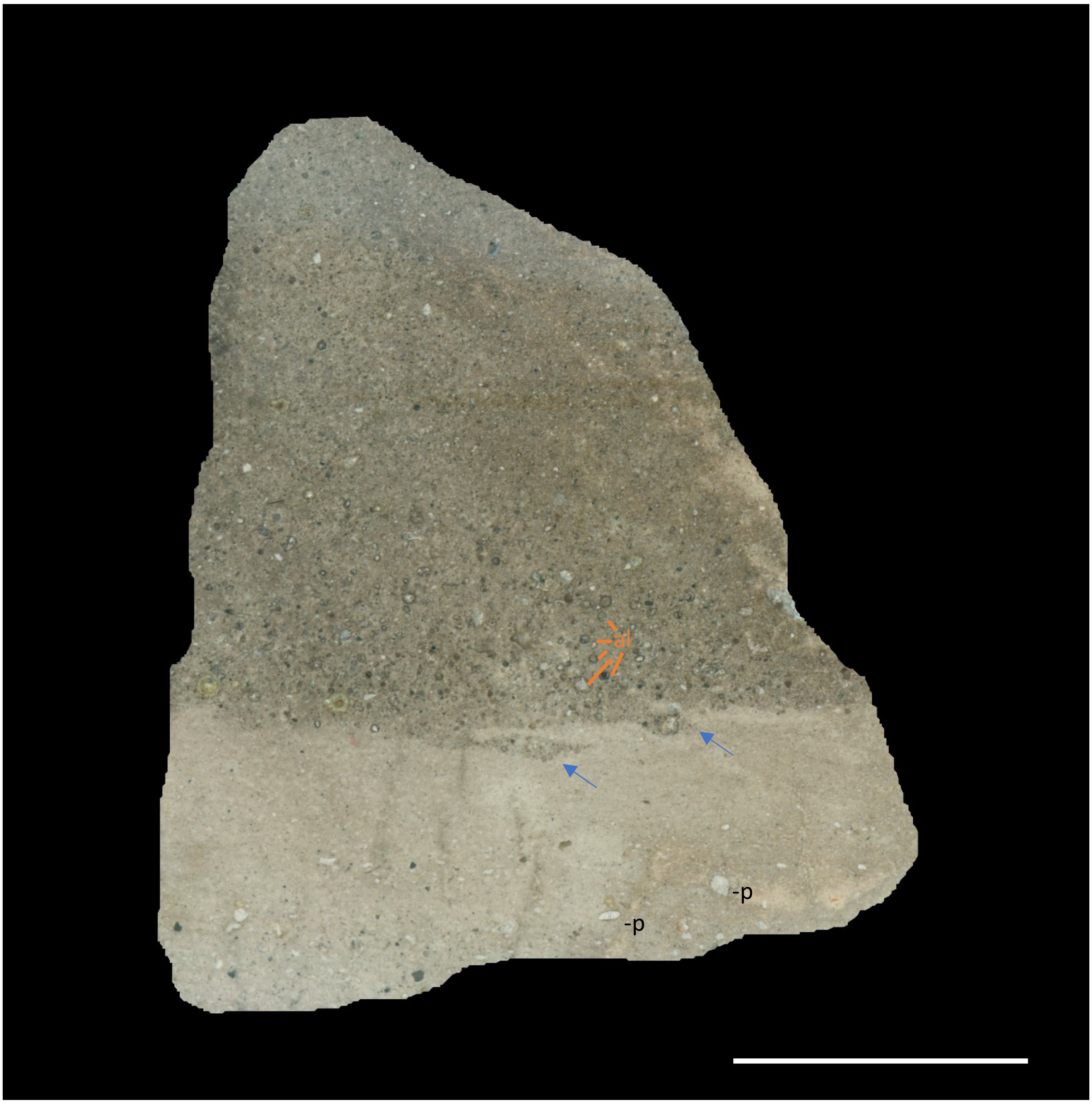

Figure 1C: Polished cut surface reveals sharp boundary between Unit A (brighter lower part, fine tuff) and Unit B (darker upper part, coarse tuff). Small-sized core type accretionary lapilli clasts of Unit B is indicated by orange lines. Unit B shows normal grading. Flame structure (indicated by blue arrows) suggest soft-sediment deformation. P = pumice, al = accretionary lapilli. Scale represents 2 cm.

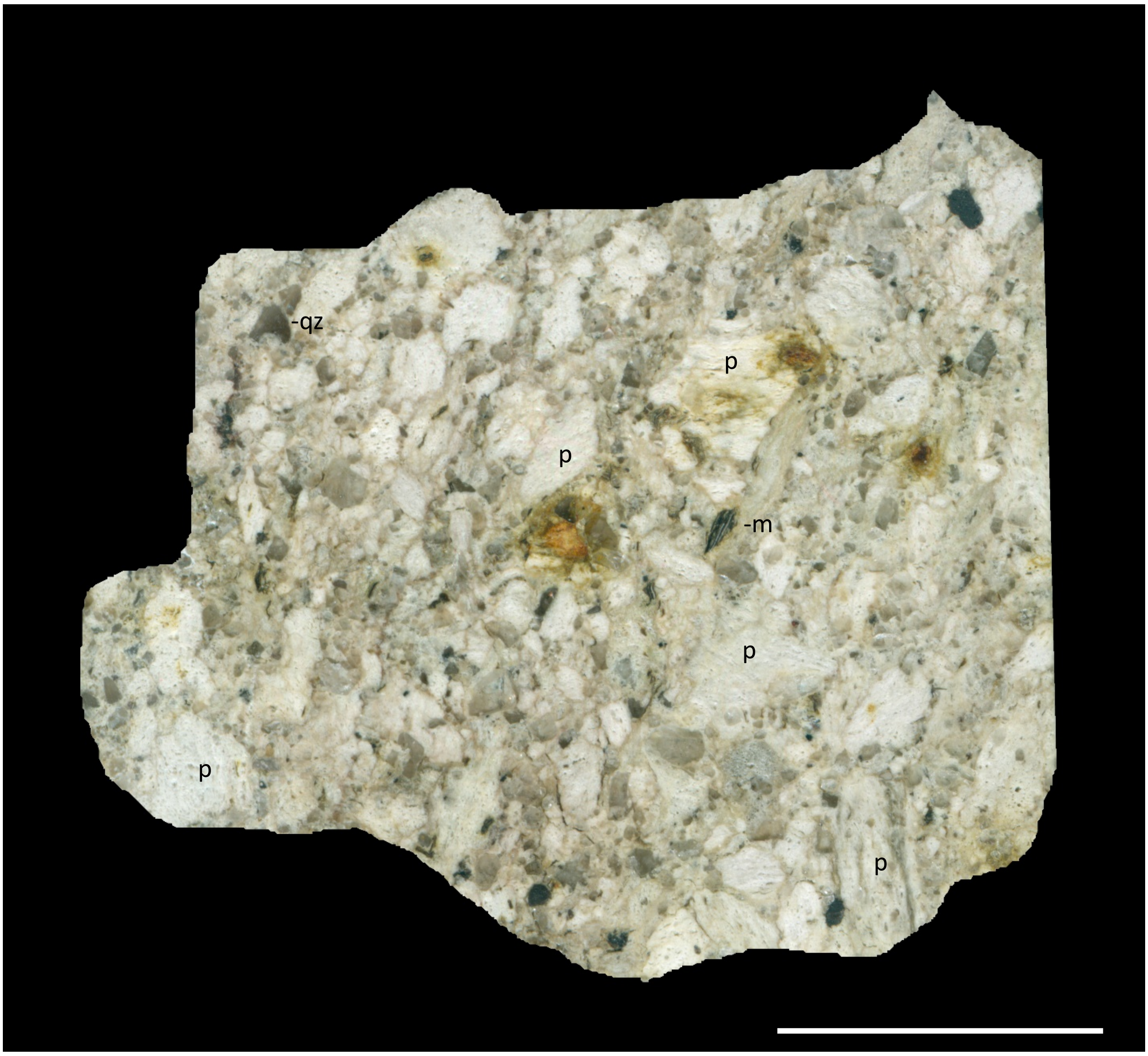

1 Figure 1D: Polished cut surface of Unit C_1 showing pumiceous lower part of the lapilli stone layer. The unit consists of pumice lapilli, quartz and dark mica as loose crystals. P = pumice, qz = quartz, m = mica. Scale is 2 cm.

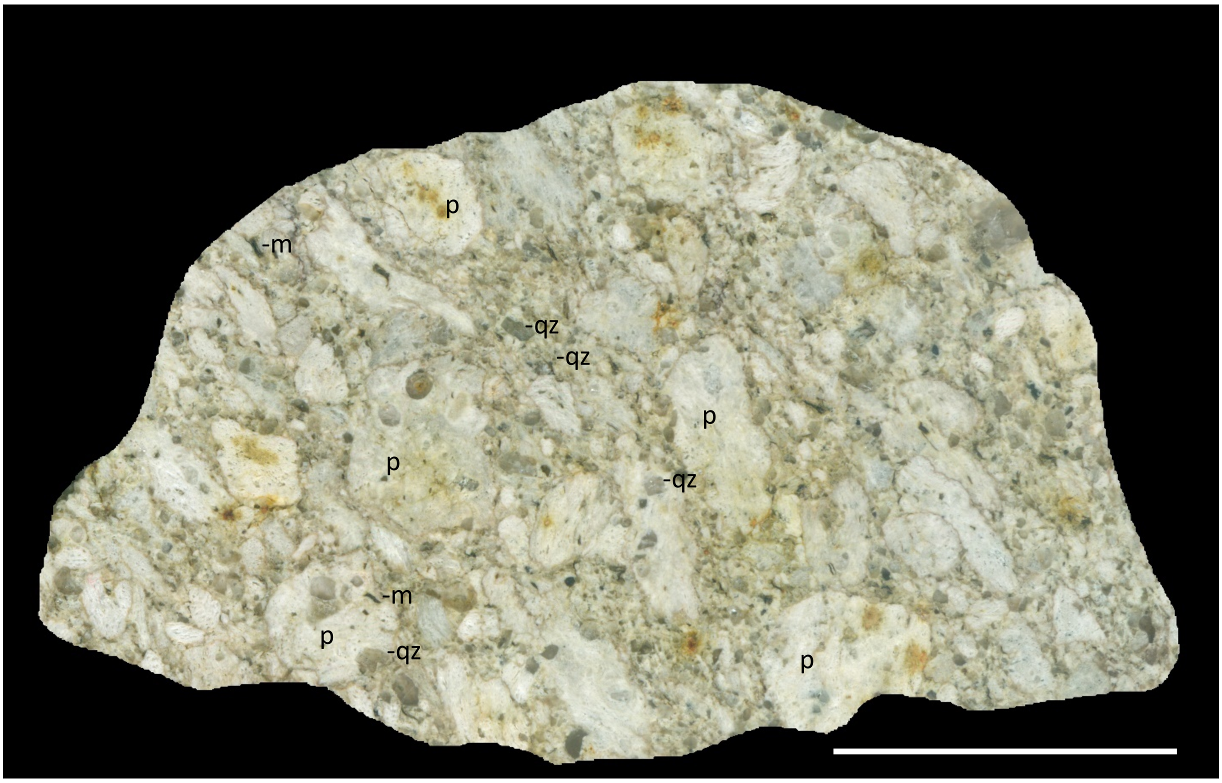

Figure 1E: Polished cut surface of the upper part of the pumiceous lapilli stone layer of Unit C. The sample shows some internal orientation possibly related to diagenetic compaction. The unit consists of pumice lapilli, quartz and dark mica as loose crystals. P = pumice, qz = quartz, m = mica. Scale is 2 cm.

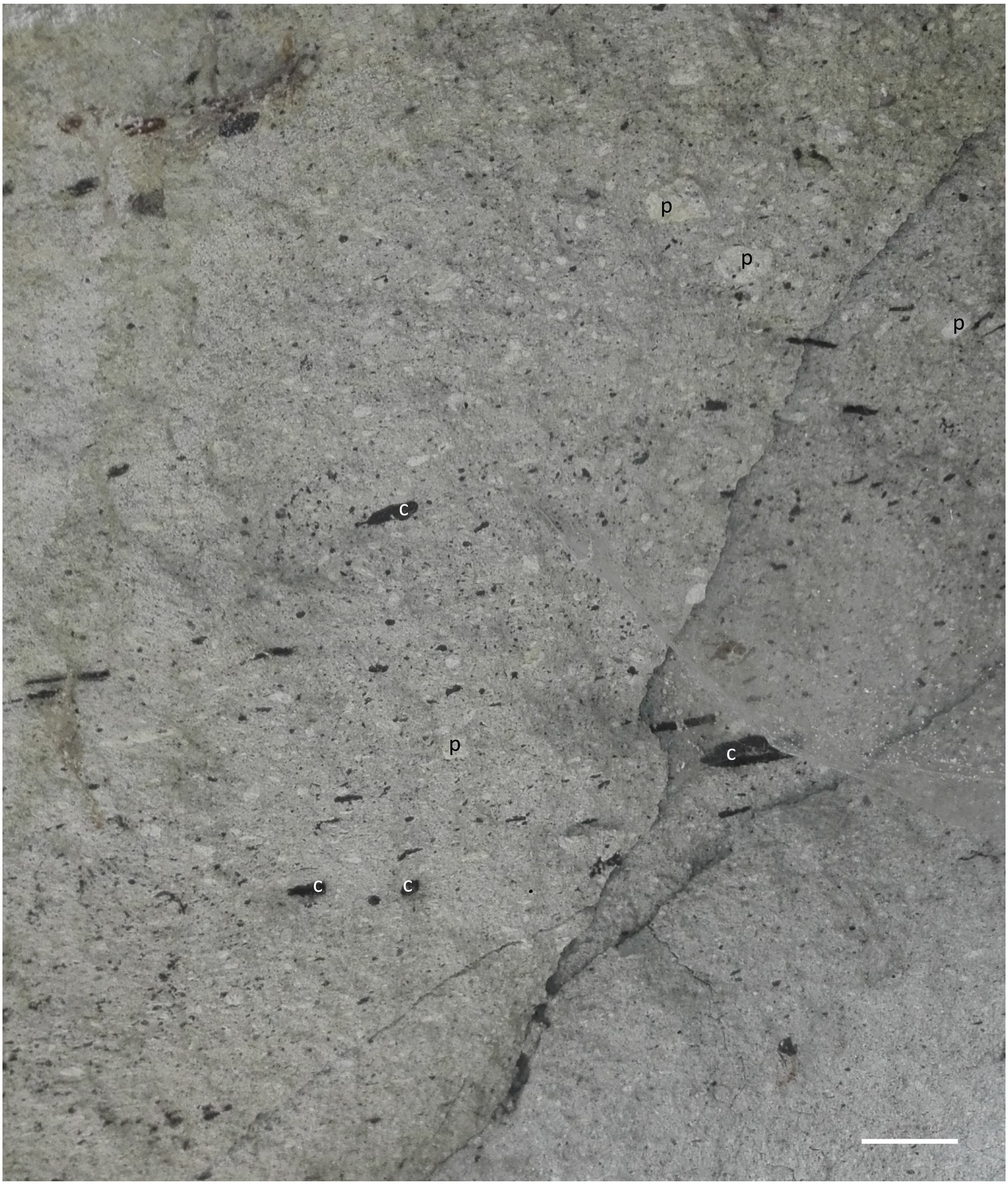

Figure 1F: Lapilli tuff of Unit D (ignimbrite). The unwelded ignimbrite shows oriented texture and consists of white pumice (p) and carbonified plant fragments (c) in a fine-grained, gray matrix. Scale is 2 cm.

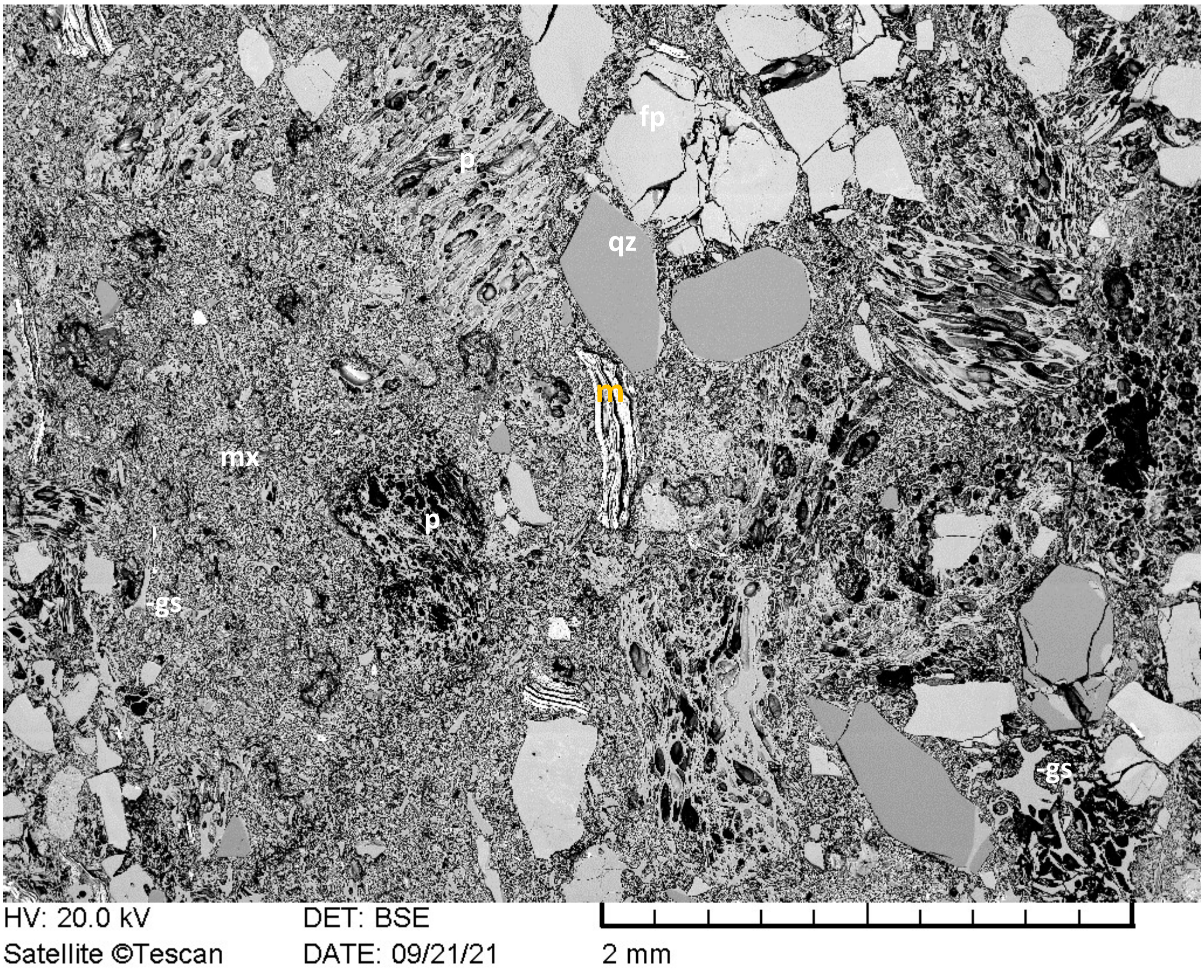

Figure 2A: BSE image of UnitA_2 tuff coarse grained facies showing the general character of this subunit. P = pumice, qz = quartz, fp = feldspar, m = mica, mx = fine grained matrix, gs = glass shard.

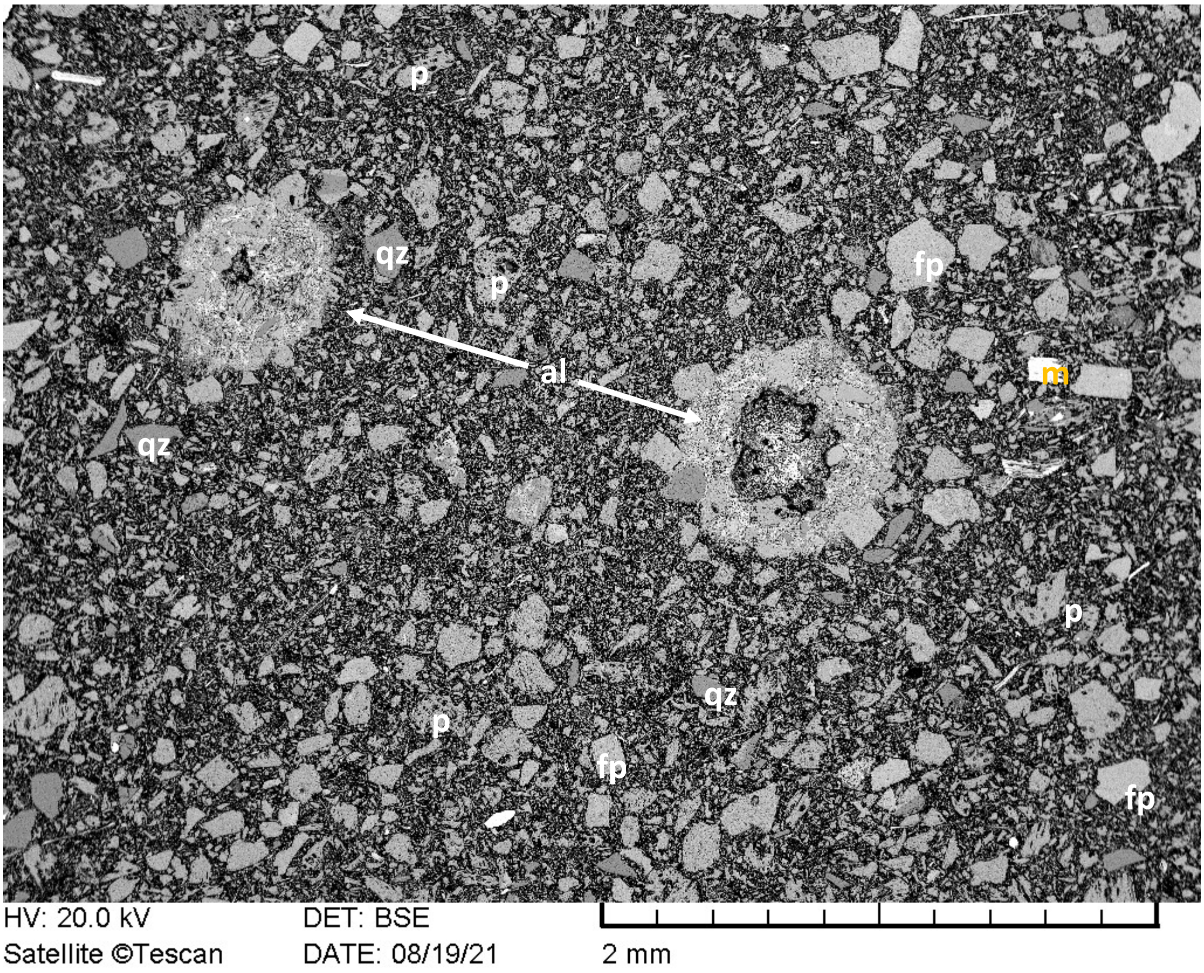

Figure 2B: BSE image of Unit B (accretionary lapilli bearing tuff) showing the general character of this unit. P = pumice, q = quartz, fp = feldspar, m = mica, mx = fine grained matrix, al = accretionary lapilli.

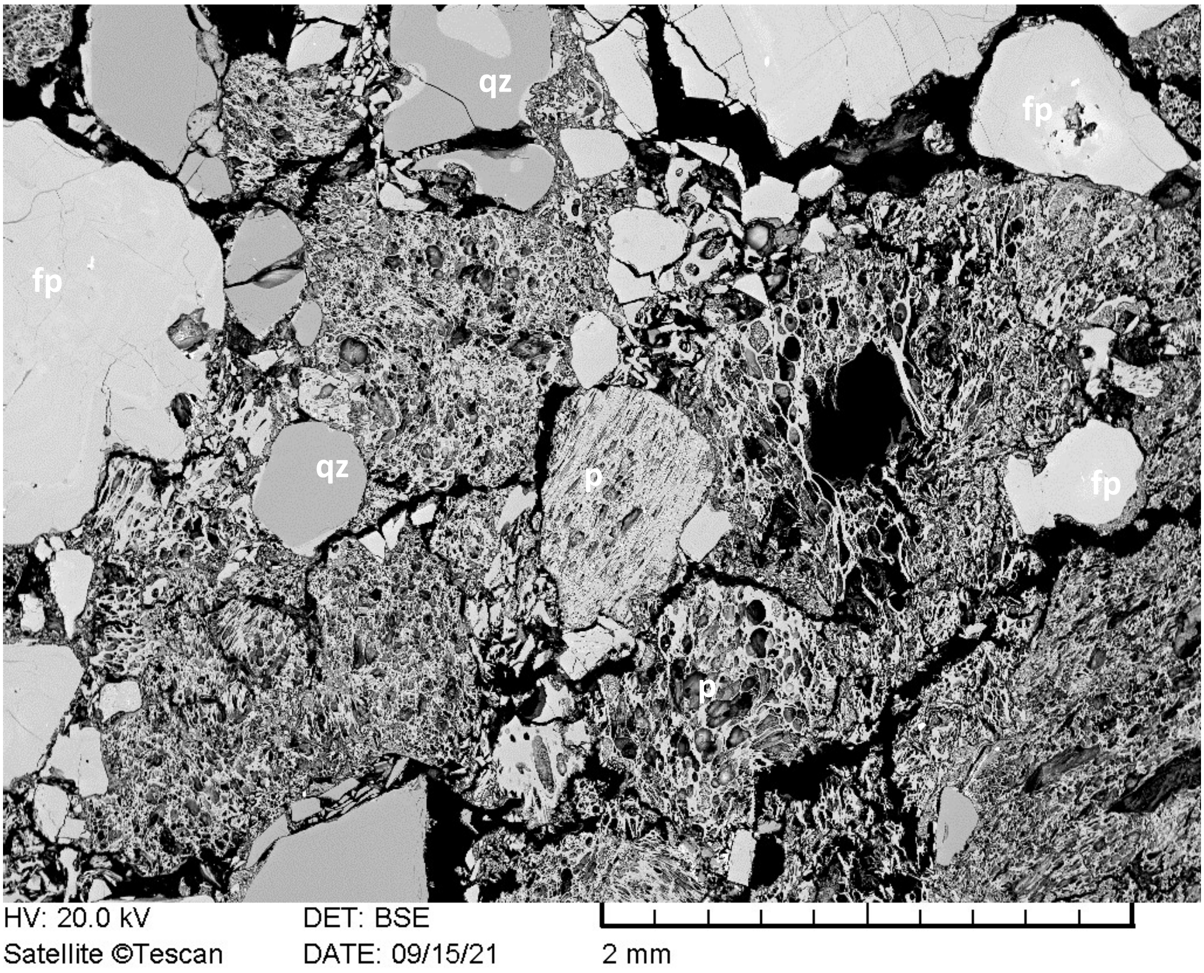

Figure 2C: BSE image of the matrix of Unit C lapilli stone showing the general character of this unit. P = pumice, q = quartz, fp = feldspar, m = mica.

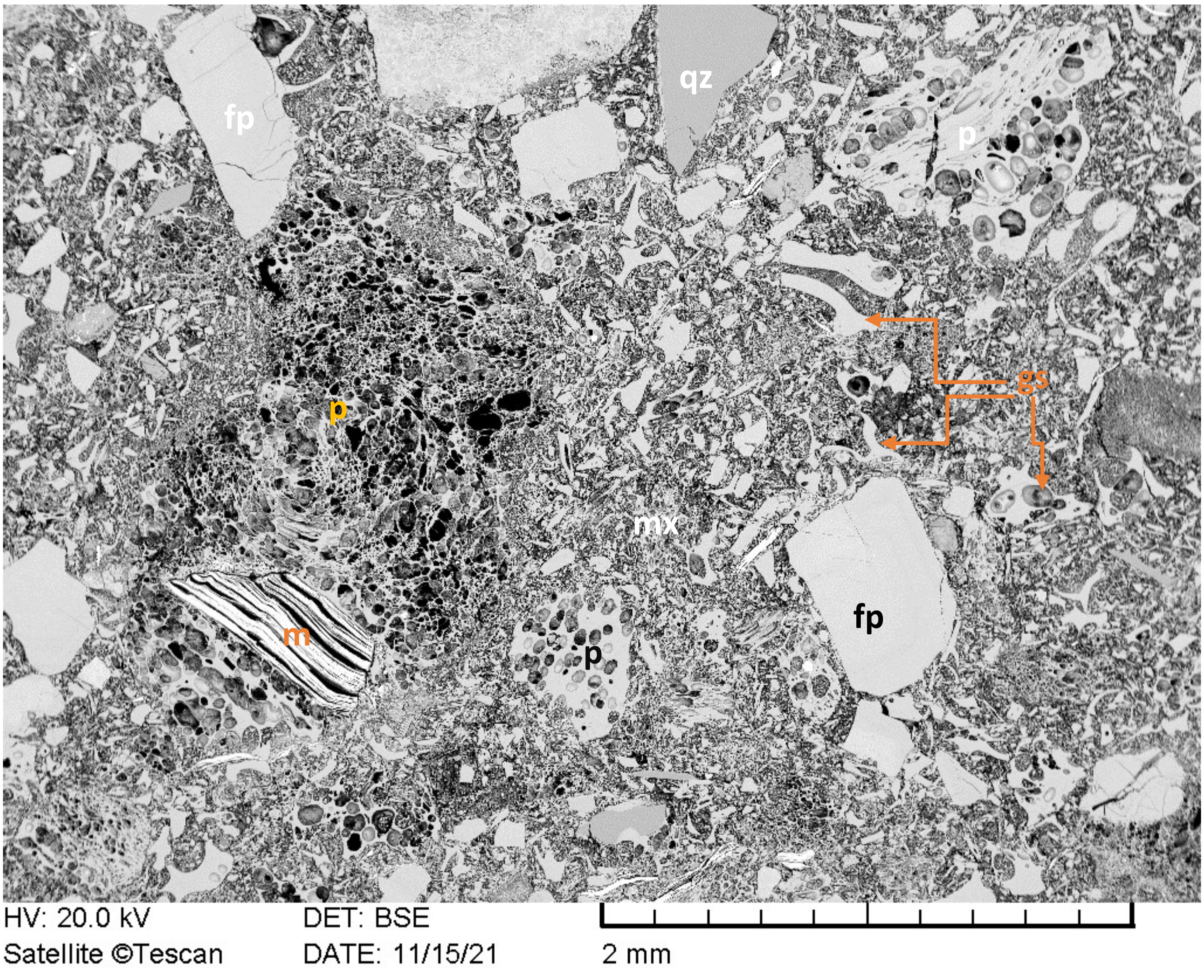

Figure 2D: BSE image of the base of Unit D ignimbrite showing the general character of this unit. It consists of pumice clasts and crystals in a fine-grained glass shard matrix. P = pumice, q = quartz, fp = feldspar, m = mica, mx = fine grained matrix, gs = glass shard.

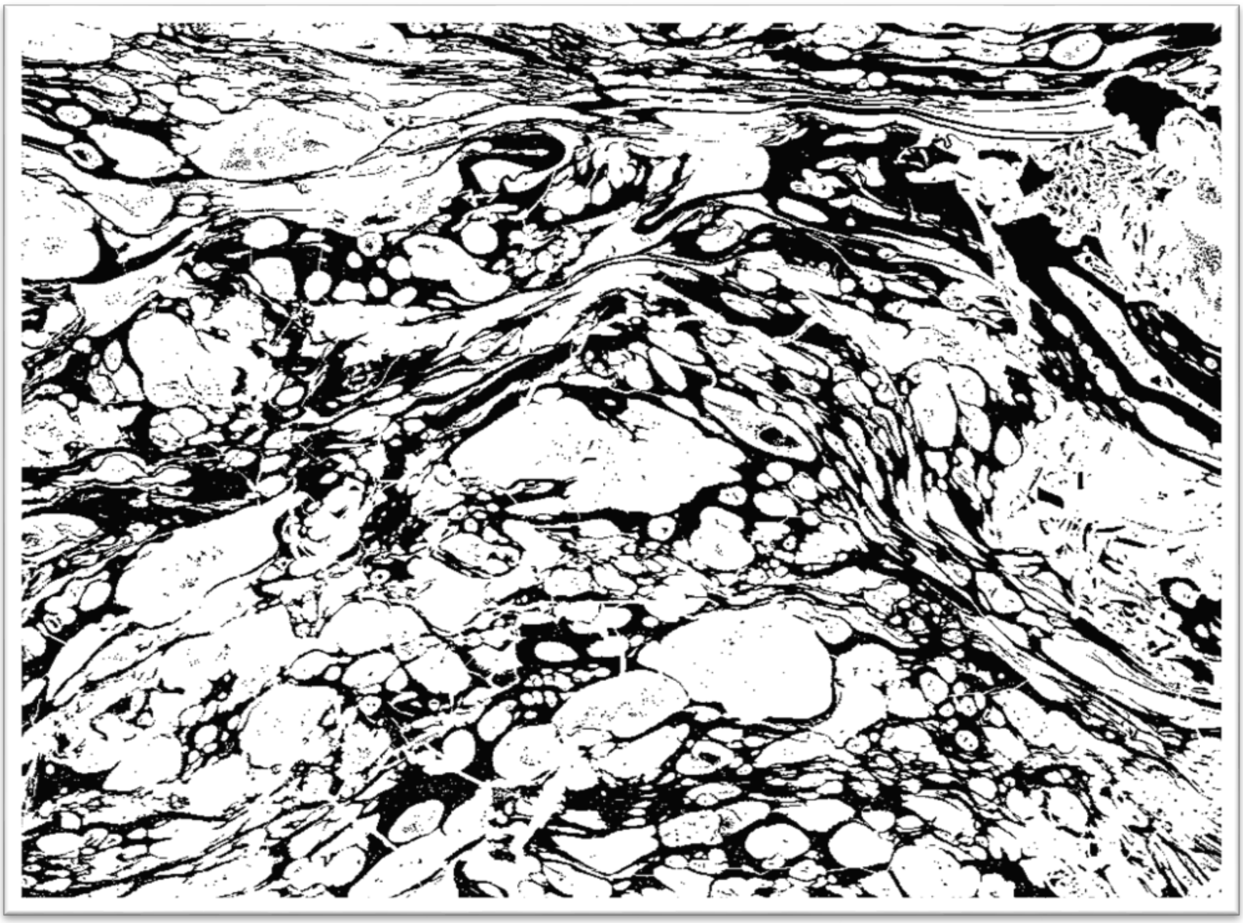

Figure 3A–E: details of Unit C.

Figure 3A: coarse vesicular pumice with fluidal texture and ellipsoidal bubbles. Threshold binary image. Glass is black, vesicles are white. Length of the bottom side is 1.2 mm.

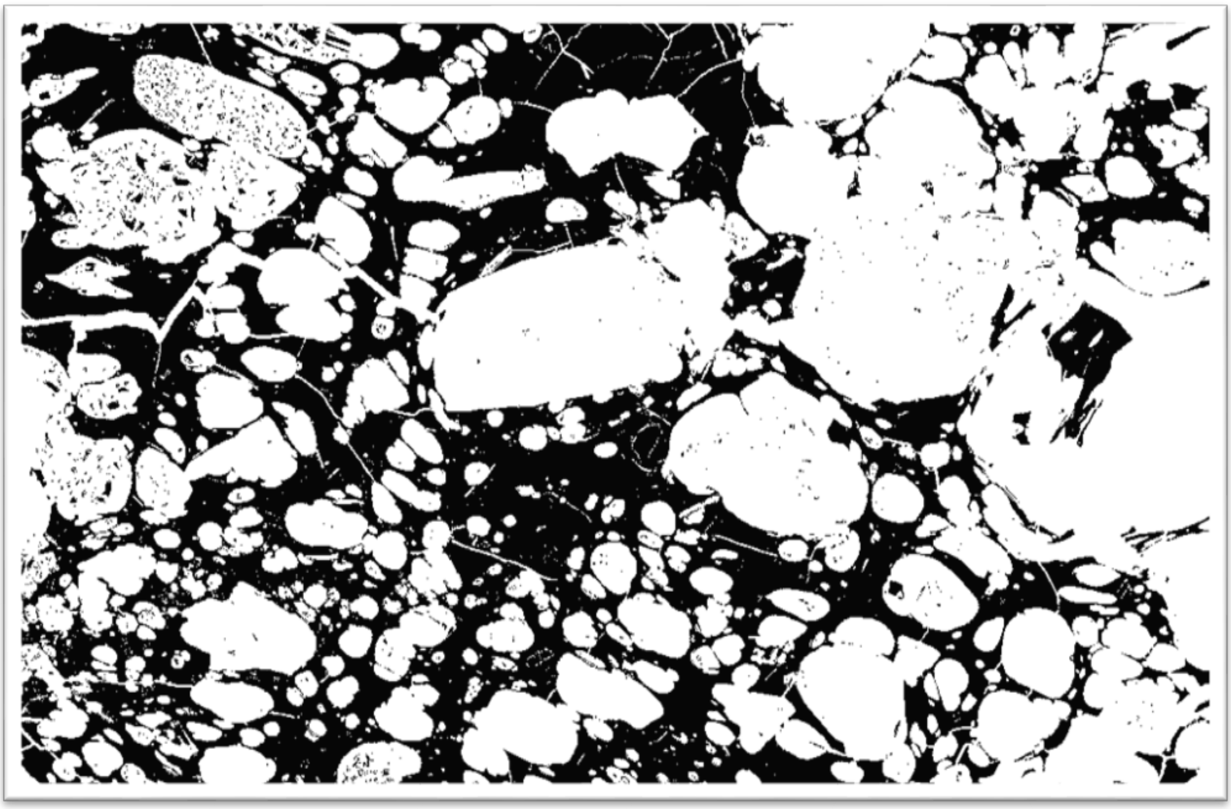

Figure 3B: Dense, coarsely vesicular texture with equant bubbles. Threshold binary image. Glass is black, vesicles are white. Length of the bottom side is 1.2 mm.

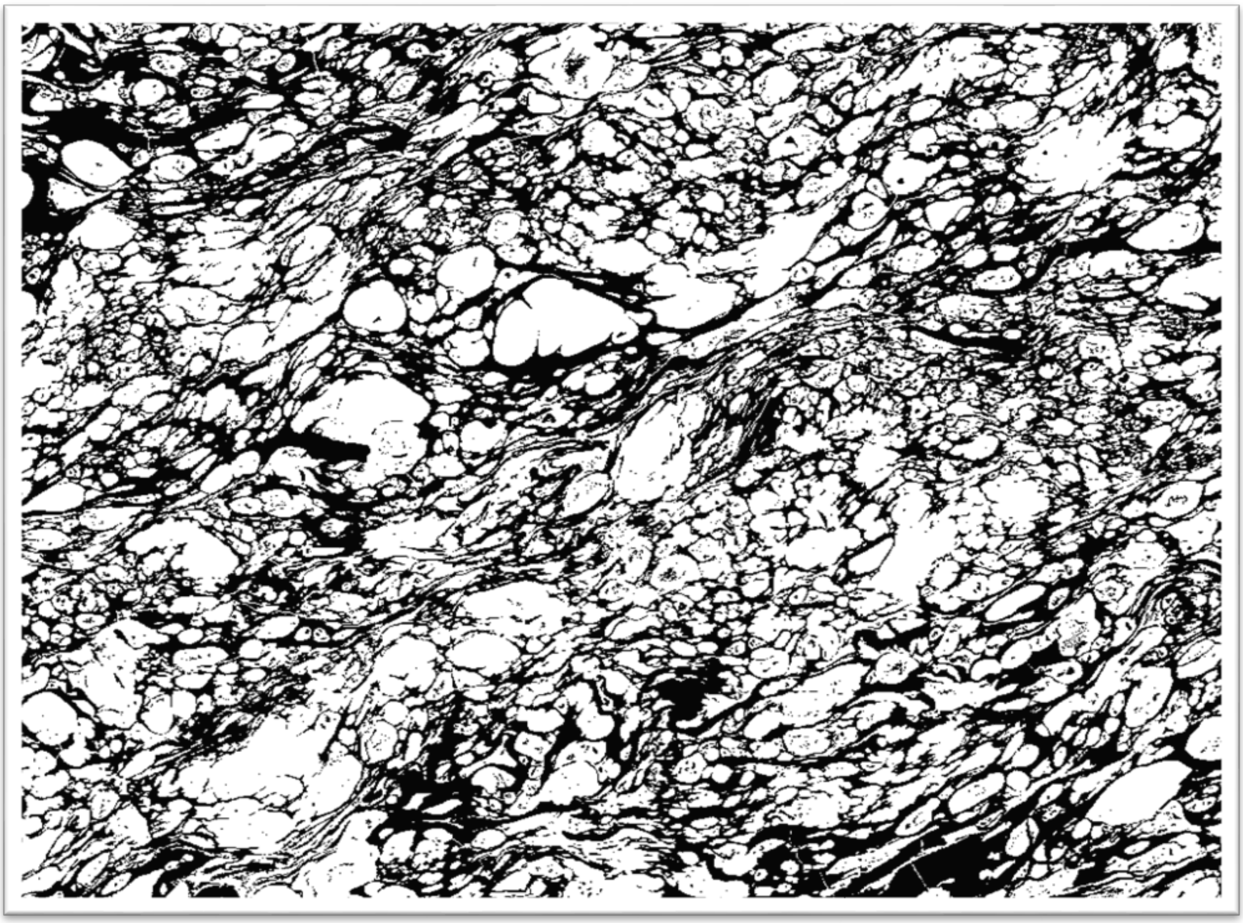

Figure 3C: Microvesicular texture with equant, slightly deformed vesicles. Threshold binary image. Glass is black, vesicles are white. Length of the bottom side is 1.2 mm.

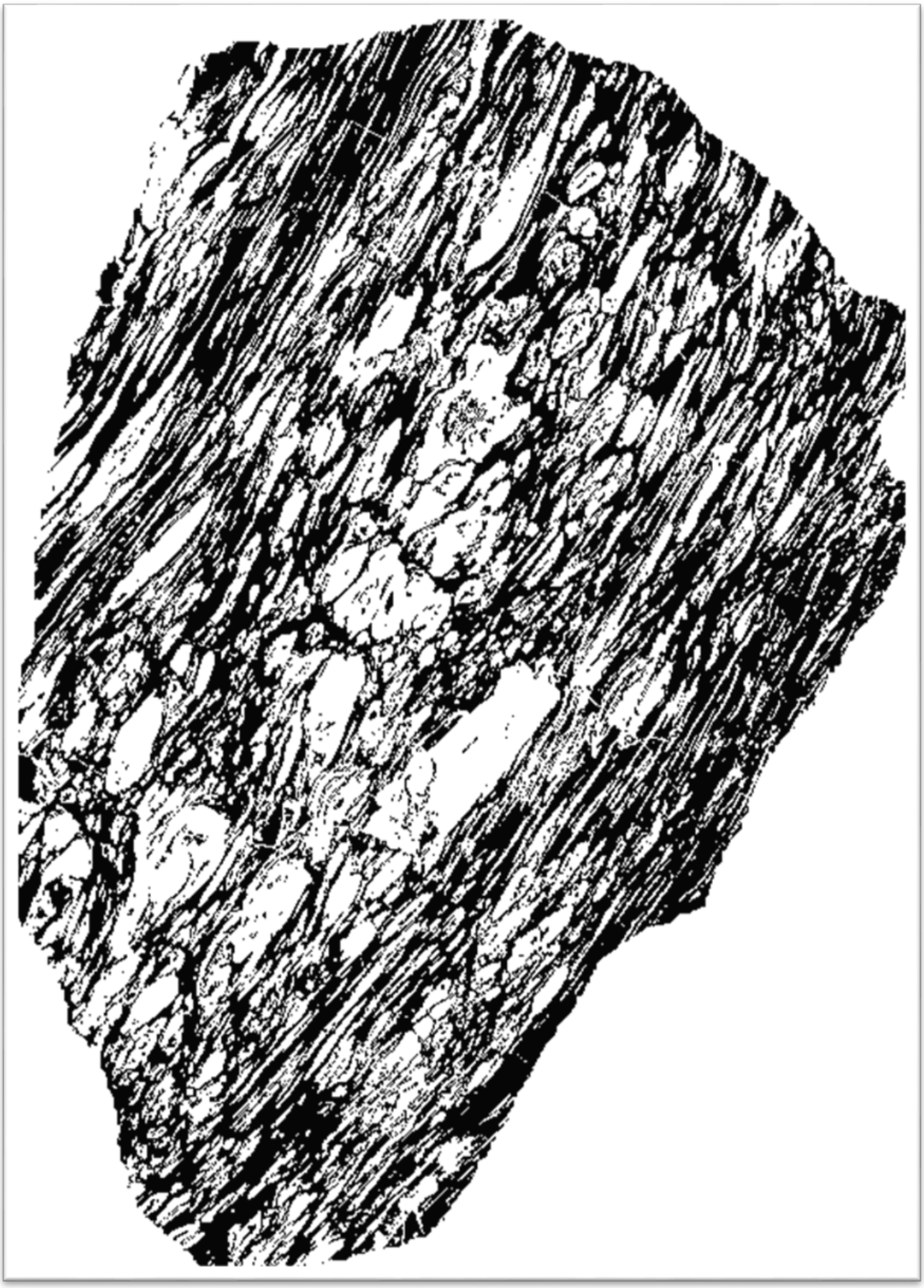

Figure 3D: Tube pumice, microvesicular texture with strongly elongeted bubbles. Threshold binary image. Glass is black vesicles are white. Length of the bottom side is 1.2 mm.

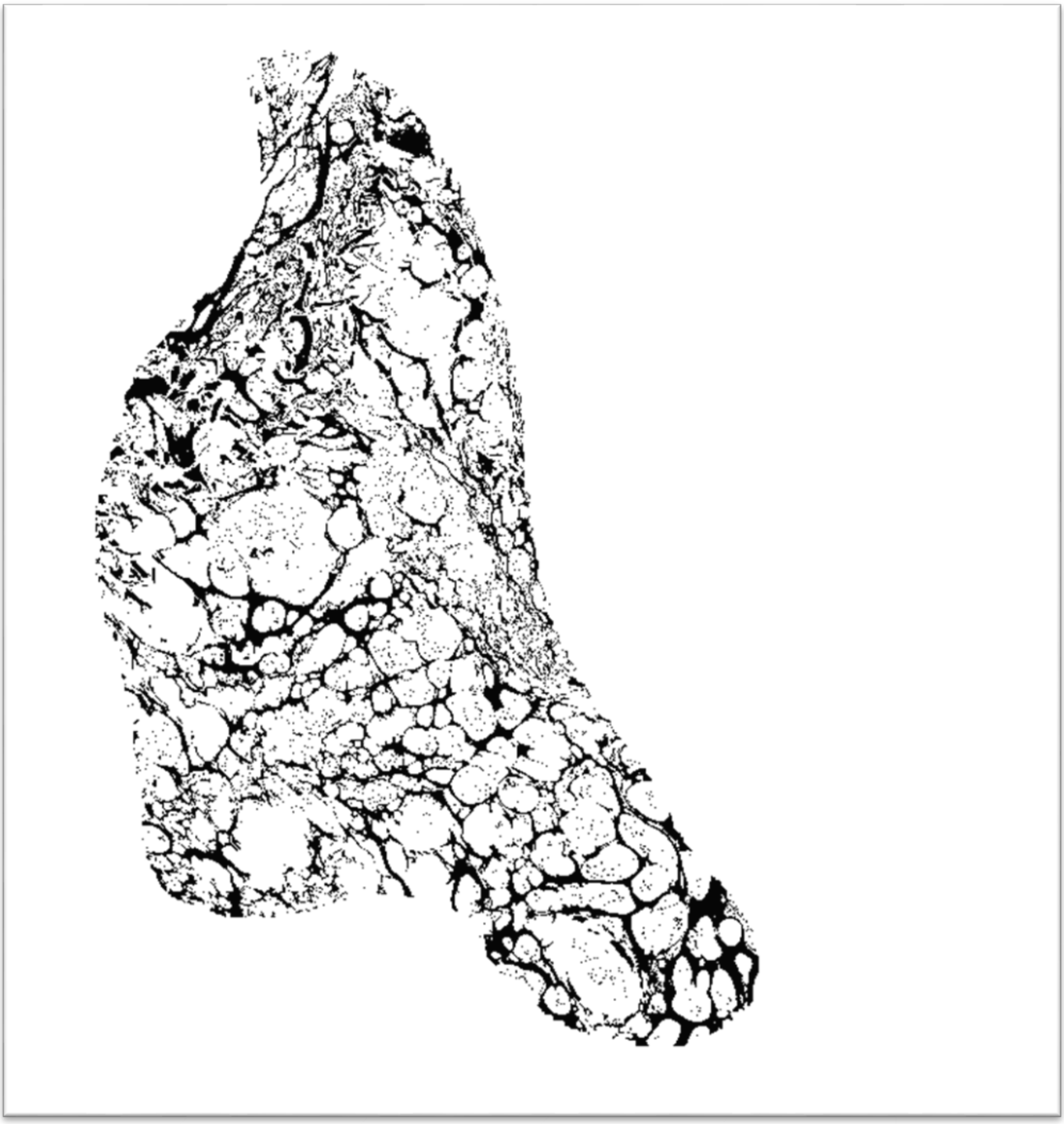

Figure 3E: Coarsely vesicular pumice with equant bubbles. Threshold binary image. Glass is black, vesicles are white. Length of the bottom side is 1.2 mm.

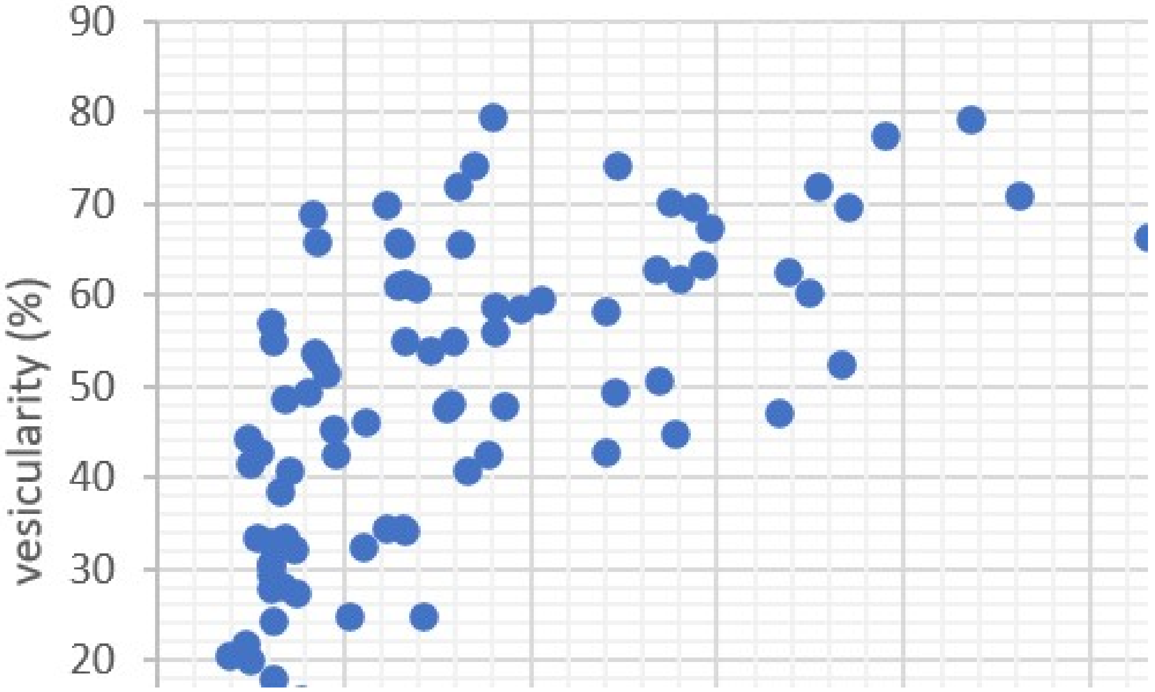

Supplement 1 Figure 4A Clast size vs vesicularity diagram of unit A.

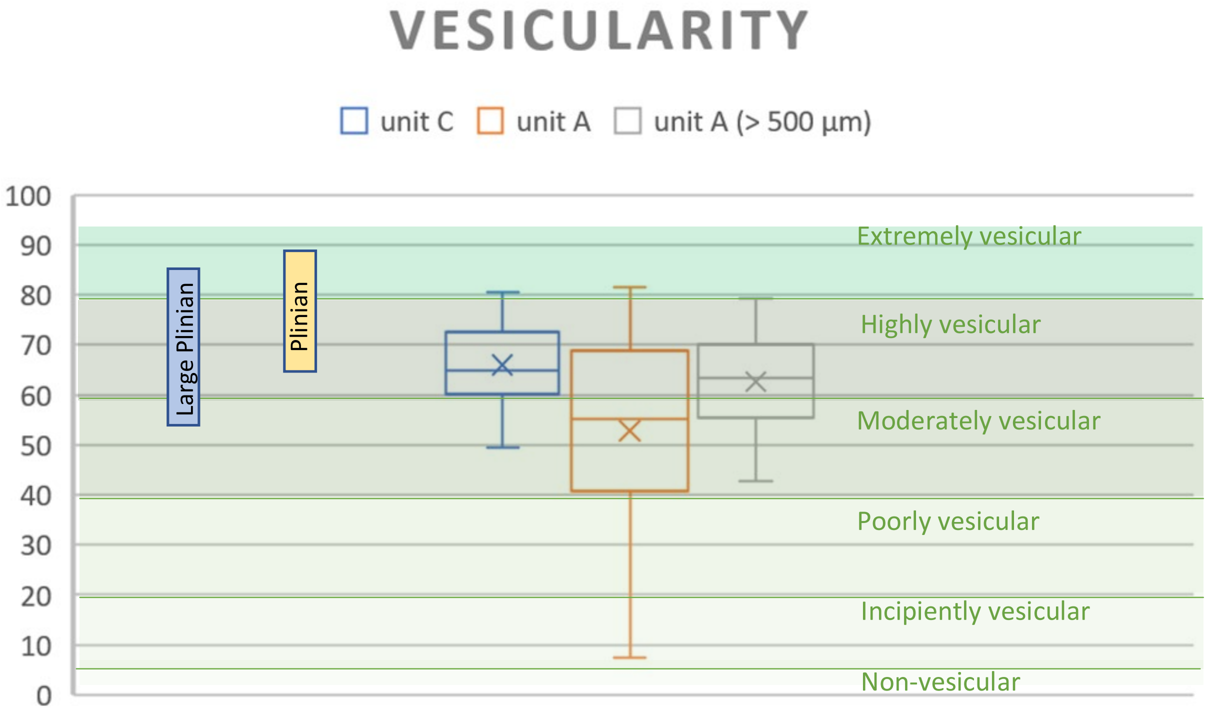

Figure 4B: Vesicularity of Unit A (all data (orange) and subset(grey) of clasts > 500 µm) and Unit C of the Eger-Ipolytarnóc pyroclastic succession (this study) compared to Plinian eruptions based on Cashman (2004); vesicularity ranges are after Houghton and Wilson (1989).
