# Supplementary material for: Large-magnitude (VEI ≥ 7) ‘wet’ explosive silicic eruption preserved a Lower Miocene habitat at the Ipolytarnóc Fossil Site, North Hungary
Source: Sci Rep. 2022 Jun 13;12:9743. doi: 10.1038/s41598-022-13586-3 (PMC9192734; doi:10.1038/s41598-022-13586-3)
Supplement: Supplementary file 5 — Supplementary Information 2B. [file 41598_2022_13586_MOESM5_ESM.docx]

Fresh glass fragments from the ignimbrites were analyzed at GEOMAR (Kiel, Germany) using JEOL JXA 8200 electron microprobe equipped with 5 wavelength dispersive spectrometers including 3 high-sensitivity ones (2 PETH and TAPH). The analytical conditions were 15 kV accelerating voltage, 6 nA current and 5 μm electron beam size for all analyses. The current/size conditions correspond to the current density of 0.076 nA µm^-2^, which is within the recommended range (<0.1 nA µm^-2^) to minimize the Na loss during analysis, especially in a combination with short counting time for Na (Morgan and London, 1996; 2005; Kuehn et al., 2011). Counting times in the latest version of the program are 5/10 s (peak/background) for Na, 20/10s for Si, Al, Mg, Ca, P, 30/15 s for Fe, K, Ti Cl, S, 40/20 s for F and 60/20 s for Mn. Basaltic glass (USNM 113498/1 VG-A99) for Ti, Fe, Mg, Ca, P, rhyolitic glass (USNM 72854 VG568) for Si, Al, Na, K, scapolite (USNM R6600-1) for S and Cl, all from the Smithsonian collection of natural reference materials (Jarosewich et al., 1980), comendite obsidian KN-18 (Nielsen and Sigurdsson, 1991; Mosbah et al., 1991) for F and synthetic rhodonite for Mn were used for calibration and monitoring of routine measurements. Two analyses of all standard glasses and scapolite were performed at the beginning of analytical session, after every 40 analyses of unknown samples and at the end. Typically, 20-22 glass shards were analyzed for every sample. The data reduction included on-line CITZAF correction and small drift correction for systematic deviations (if any) from the reference values obtained on standard materials. Data on reference glasses analyzed along with the samples in this study are provided in Supplementary table XX.

Trace element analyses were obtained at the Institute of Geosciences, CAU Kiel, Germany using using ICP-MS Agilent 8900 and a Coherent GeoLas ArF 193 nm Excimer LA system operated with a fluence of 5 J cm-2, at a repetition rate of 10 Hz and a 15-24 μm ablation craters. Analyses were performed using a large volume ablation (Fricker et al., 2011) modified for fast washout. Helium (0.7 L min^-1^) with addition of 14 mL min^-1^ H_2_ was used as carrier gas. The carrier gas was mixed with Ar (~1 L min^-1^) prior to introduction to the ICP-MS. Ten major elements (Si, Ti, Al, Fe, Mn, Mg, Ca, Na, K, P) and 31 trace elements were analyzed. Analyses included 20 s background (laser-off) and 30 s signal (laser-on) measurements. Dwell time for different elements varied from 5 to 20 ms depending on their abundance. One complete measurement cycle lasted 0.607 ms and initial data reduction was performed in Glitter software (Griffin et al., 2008), that included manual selection of integration windows and preliminary calibration. The typical integration intervals for tiny tephra shards were 6-10 s and included 10-17 cycles. The intensities corrected for background and averaged over the selected intervals were normalized to the intensity of ^43^Ca isotope and converted to concentrations by matching the sum of major element oxides to 100 wt% (Liu et al., 2008; Pettke et al., 2004). The calibration and correction of instrumental drift used data on ATHO-G reference glass (Jochum et al., 2006), which was measured in duplicate after every 18 points on unknown samples. Typically, 7-10 glass shards were analyzed for every sample. Data on reference glasses analyzed along with the samples in this study are provided in Supplementary table XX.

Further analytical details of the major and trace element analyses can be found in (Portnyagin et al., 2020).

Fricker, M. B., Kutscher, D., Aeschlimann, B., Frommer, J., Dietiker, R., Bettmer, J., and Gunther, D.: High spatial resolution trace element analysis by LA-ICP-MS using a novel ablation cell for multiple or large samples, Int. J. Mass. Spectrom., 307, 39-45, 2011.

Griffin, W., Powell, W., Pearson, N., and O’reilly, S.: GLITTER: data reduction software for laser ablation ICP-MS, Laser Ablation-ICP-MS in the earth sciences. Mineralogical association of Canada short course series, 40, 204-207, 2008.

Hunt, J. B. and Hill, P. G.: An inter-laboratory comparison of the electron probe microanalysis of glass geochemistry, Quat. Int., 34-36, 229-241, 1996.

Hunt, J. B. and Hill, P. G.: Tephrological implications of beam size—sample‐size effects in electron microprobe analysis of glass shards, Journal of Quaternary Science: Published for the Quaternary Research Association, 16, 105-117, 2001.

Jochum, K. P., et al.: MPI-DING reference glasses for in situ microanalysis: New reference values for element concentrations and isotope ratios, Geochem. Geophys. Geosyst., 7, doi:10.1029/2005GC001060, 2006.

Kuehn, S. C., Froese, D. G., and Shane, P. A. R.: The INTAV intercomparison of electron-beam microanalysis of glass by tephrochronology laboratories: Results and recommendations, Quat. Int., 246, 19-47, 2011.

Liu, Y., Hu, Z., Gao, S., Günther, D., Xu, J., Gao, C., and Chen, H.: In situ analysis of major and trace elements of anhydrous minerals by LA-ICP-MS without applying an internal standard, Chem. Geol., 257, 34-43, 2008.

Longerich, H. P., Jackson, S. E., and Gunther, D.: Laser ablation inductively coupled plasma mass spectrometric transient signal data acquisition and analyte concentration calculation, J. Analyt. Atom. Spec., 11, 899-904, 1996.

Mosbah, M., Metrich, N., and Massiot, P.: PIGME fluorine determination using a nuclear microprobe with application to glass inclusions, Nucl. Instrum. Methods Phys. Res., B58, 227-231, 1991.

Morgan, G. B. and London, D.: Optimizing the electron microprobe analysis of hydrous alkali aluminosilicate glasses, Am. Mineral., 81, 1176-1185, 1996.

Morgan, G. B. and London, D.: Effect of current density on the electron microprobe analysis of alkali aluminosilicate glasses, Am. Mineral., 90, 1131-1138, 2005.

Nielsen, C. H. and Sigurdsson, H.: Quantitative methods for electron microprobe analysis of sodium in natural and synthetic glasses, Am. Mineral., 66, 547-552, 1981.

Pettke, T., Halter, W. E., Webster, J. D., Aigner-Torres, M., and Heinrich, C. A.: Accurate quantification of melt inclusion chemistry by LA-ICPMS: a comparison with EMP and SIMS and advantages and possible limitations of these methods, Lithos, 78, 333-361, 2004.
