# Supplementary material for: Large-magnitude (VEI ≥ 7) ‘wet’ explosive silicic eruption preserved a Lower Miocene habitat at the Ipolytarnóc Fossil Site, North Hungary
Source: Sci Rep. 2022 Jun 13;12:9743. doi: 10.1038/s41598-022-13586-3 (PMC9192734; doi:10.1038/s41598-022-13586-3)
Supplement: Supplementary file 7 — Supplementary Information 3B. [file 41598_2022_13586_MOESM7_ESM.docx]

Zircon U-Pb methodology and results

Zircons were separated at the Laboratoire Magmas et Volcans (LMV), Clermont-Ferrand (France) using standard techniques of crushing and sieving, followed by Wilfley table, magnetic separation and heavy liquids before handpicking under binocular microscope. Zircons were then mounted in epoxy disks, ground, and polished with 0.25µm diamond grit to expose crystal interiors. U-Th-Pb isotopic data on zircons were obtained by laser ablation inductively coupled plasma mass spectrometry (LA-ICP-MS) at LMV (Laboratoire Magmas & Volcans, Clermont-Ferrand, France). The analyses involved ablation of minerals with a Resonetics M-50 laser system operating at a wavelength of 193 nm. Spot diameters of 33 µm, repetition rates of 3 Hz and fluence of 2.0 J/cm^2^ resulted in a spot depth of 10 µm. The ablated material was carried into helium and then mixed with nitrogen (Paquette et al. 2014) and argon before injection into the plasma source of a Thermo Element XR Sector Field high-resolution ICP-MS equipped with the jet interface pumping device. The alignment of the instrument and mass calibration were performed before every analytical session using the NIST SRM 612 reference glass, by inspecting the signals of ^238^U, ^232^Th and ^208^Pb and by minimising the ThO^+^/Th^+^ ratio. The analytical method for isotope dating with laser ablation ICP-MS is basically similar to that reported in Hurai et al. (2010) and Paquette et al. (2019). The ^235^U signal is calculated from ^238^U based on the ratio ^238^U/^235^U = 137.818 (Hiess et al. 2012). Single analyses consisted of 30 seconds of background integration with laser off followed by 60 seconds integration with the laser firing and a 30 seconds delay to wash out the previous sample and prepare the next analysis.

Data were corrected for U-Pb fractionation occurring during laser sampling and for instrumental mass bias by standard bracketing with repeated measurements of GJ-1 zircon primary standard (Jackson et al. 2004). Repeated analyses of 91500 zircon reference material (Wiedenbeck et al. 1995) during each analytical session and treated as unknown, independently control the reproducibility and accuracy of the corrections. Data reduction was carried out with the software package GLITTER® from Macquarie Research Ltd (van Achterbergh et al. 2001; Jackson et al. 2004).

Schärer (1984) demonstrated that most zircons are affected during their growth by deficits and excesses of both ^230^Th and ^231^Pa relative to the initial Th/U secular equilibrium. In order to obtain accurate crystallization ages, it is necessary to correct for the effect of initial disequilibria caused by intermediate nuclides in the ^238^U and ^235^U decay series. Sakata et al. (2017) and Sakata (2018) propose a simplified correction model and a related Microsoft Excel spreadsheet based on the mathematical framework of Wendt and Carl (1985) using the following equations:

^206^Pb^*^/^238^U = (*e*^λ238^*^t^* -1) + λ_238_/λ_230_ (*f*_Th/U_ – 1) (1 – *e*^-λ230^*^t^*) *e*^λ238^*^t^*

^207^Pb^*^/^235^U = (*e*^λ235^*^t^* -1) + λ_235_/λ_231_ (*f*_Pa/U_ – 1) (1 – *e*^-λ231^*^t^*) *e*^λ235^*^t^*

The concentrations in U-Th-Pb are calibrated relative to the certified contents of GJ-1 zircon (Jackson et al. 2004) reference material. The isotopic ratios and 2σ level uncertainties were corrected from elemental and isotopic fractionation, as well as Th/U disequilibria. A systematic external error is subsequently propagated by quadratic addition of uncertainties on U and Th decay constants as well as of the variability of the primary reference material used for corrections (GJ-1) and of the long-term variability of the secondary reference material (91500). According to Horstwood et al. (2016), this systematic external error is added to the 2σ error associated to the weighted mean ^206^Pb/^238^U ages calculation. The Tera and Wasserburg (1972) diagrams were generated using Isoplot/Ex v. 2.49 software package by Ludwig (2001).
