# Supplementary material for: Large-magnitude (VEI ≥ 7) ‘wet’ explosive silicic eruption preserved a Lower Miocene habitat at the Ipolytarnóc Fossil Site, North Hungary
Source: Sci Rep. 2022 Jun 13;12:9743. doi: 10.1038/s41598-022-13586-3 (PMC9192734; doi:10.1038/s41598-022-13586-3)
Supplement: Supplementary file 8 — Supplementary Information 4A. [file 41598_2022_13586_MOESM8_ESM.docx]

Argon Supplementary Data

**^40^Ar/^39^Ar methods**

The analytical data are organized to comply with FAIR data reporting norms (see for instance Schaen et al., 2020). Excel workbooks are provided with data formatted within a variety of worksheets to facilitate ease of data viewing. Data are presented in isotope ratio format along with raw intensity format.

Sanidine and plagioclase were separated with standard methods that involved coarse crushing and sieving to the 32-45 mesh fraction. The sample was treated in dilute HF for 5 minutes to remove adhering glass and matrix and the thoroughly washed in distilled water in the ultrasonic. Sanidine and plagioclase were hand-picked while grains were immersed in wintergreen oil while being viewed under a polarizing binocular microscope. Following hand-picking, the crystals were ultrasonically cleaned in acetone followed by final rinsing in distilled water.

The samples were irradiated in the central thimble at the USGS TRIGA reactor in Denver, Colorado for 16 hours along with Fish Canyon sanidine interlaboratory standard FC-2 located in 8 holes within a 24-hole irradiation disk. Six grains from each monitor hole are analyzed and the J-value of the unknown locations is determined with a planar fit to the appropriate flux monitor locations. FC-2 is assigned an age of 28.201 Ma (Kuiper et al., 2008) and all ages are calculated with a ^40^K decay constant of 5.463e-10 /a (Min et al., 2000) while isotope abundances are after Steiger and Jager (1977).

After irradiation, monitors and unknowns were loaded into stainless steel trays, evacuated and baked at 100°C for 4 hours. The samples were analyzed using a Thermo-Fisher Scientific ARGUS VI multi-collector mass spectrometer equipped with five Faraday cups, and one electron multiplier (CDD) operated in ion-counting mode. The configuration has ^40^Ar, ^39^Ar, ^38^Ar, ^37^Ar and ^36^Ar on the H1, Axial, L1, L2, and CDD detectors, respectively. Resistors were 10^13^ Ohms for ^40^Ar, ^39^Ar, ^37^Ar and 10^14^ Ohms for ^38^Ar. ^36^Ar is measured on the CDD that has dead time of 14 ns. Extracted gas was cleaned with a getter configuration that included two NP 10 getters one operated at 1.6 A and one at room temperature. Gas cleanup times were typically 30 seconds following a 30 second fusion or step-heating increment.

Calibration gases of air and a gas mixture enriched in radiogenic ^40^Ar along with ^39^Ar were analyzed interspersed with the unknowns to monitor instrument drift and determine detector intercalibration factors. All data collection was conducted with the in-house Pychron software and data reduction utilized MassSpec version 7.875. High intensity isotope concentrations from fusion of sanidine were analyzed with 120 seconds of counting followed by 45 seconds of baseline measurement were as the low abundances derived from the plagioclase grains utilized 400 seconds of beam counting followed by 60 seconds of baseline measurement.

Extraction line blank measurements were made throughout the experiments and blank intensities are included in the raw intensity worksheet for each measurement.

The reported age of the sanidine data is calculated based on a weighted mean with the weighting factor being the inverse variance (e.g., Taylor, 1982) and the error is the square root of the sum of 1/σ ^2^ values. The error is also multiplied by the square root of the MSWD for MSWD greater than 1 and errors are reported at 2σ. J-error and irradiation correction factor uncertainties are included for all weighted mean age errors. For the plagioclase two-step plateau ages are determined by the same weighted mean methods, however the preferred eruption age is given by the isochron analyses of all of the heating steps.

References cited

Kuiper, K. F., Deino A., Hilgen, F. J., Krijgsman, W., Renne, P. R., and Wijbrans, J. R., 2008, Synchronizing the rock clocks of Earth history. Science 320, 500–504.

Min, K., Mundil, R., Renne, P. R. and Ludwig, K. R., 2000, A test for systematic errors in ^40^Ar/^39^Ar geochronology through comparison with U–Pb analysis of a 1.1 Ga rhyolite. Geochim. Cosmochim. Acta 64, 73–98.

Schaen, A.J., Jicha, B.R., Hodges, K.V., Vermeesch, P., Stelten, M.E., Mercer, C.M., Phillips, D., Rivera, T.A., Jourdan, F., Matchan, E.L., Hemming, S.R., Morgan L.E., Kelley, S.P., Cassata, W.S., Heizler, M.T., Vasconcelos, P.M., Koppers, A.A.P., Mark, D.F., Niespolo, E.M., Sprain, C.J., Benowitz, J.A., Hames, W.E., Kuiper, K.F., Turrin, B.D., Renne, P.R., Ross, J., Nomade, S., Guillou, H., Laura E. Webb, L.E., Cohen, B.A., Calvert, A.T., Joyce, N., Morgan Ganderød, M., Wijbrans, J., Ishizuka, O., He, H., Ramirez, A., Pfänder, J.A., Lopez-Martínez, M., Huaning Qiu, H., Brad S. Singer, B.S., (in press), On the reporting and interpretation of ^40^Ar/^39^Ar geochronologic data, Geol. Soc. Am. Bull.

Steiger, R.H., and Jäger, E., 1977. Subcommission on geochronology: Convention on the use of decay constants in geo- and cosmochronology. Earth and Planet. Sci. Lett., v. 36, 359-362.

Taylor, J.R., 1982. An Introduction to Error Analysis: The Study of Uncertainties in Physical Measurements, Univ. Sci. Books, Mill Valley, Calif., 270 p.
