# Supplementary material for: Large-magnitude (VEI ≥ 7) ‘wet’ explosive silicic eruption preserved a Lower Miocene habitat at the Ipolytarnóc Fossil Site, North Hungary
Source: Sci Rep. 2022 Jun 13;12:9743. doi: 10.1038/s41598-022-13586-3 (PMC9192734; doi:10.1038/s41598-022-13586-3)
Supplement: Supplementary file 9 — Supplementary Information 4B. [file 41598_2022_13586_MOESM9_ESM.pdf]

$^{40}Ar/^{39}Ar$ Geochronology of Volcanic Rocks associated with the Ipolytarnoc Fossil Track Site

By

Matt Heizler

SEPT 3, 2021

Prepared for

David Karatson
Eötvös University
Hungary

NEW MEXICO
GEOCHRONOLOGICAL RESEARCH LABORATORY
(NMGRL)

DIRECTOR

DR. MATTHEW T. HEIZLER

LABORATORY TECHNICIAN

DR. JAKE ROSS

Internal report: IR#1304

Introduction

David Karatson from Eötvös University submitted two volcanic samples to the NMGRL for $^{40}Ar/^{39}Ar$ dating. The samples are from the fossil site of Ipolytarnóc and are part of a geochronological study to constrain the ages of fossils at this important location. Sanidine was dated from one sample and co-genetic plagioclase and biotite were also dated from each sample. The samples are expected to yield middle-Miocene eruption ages.

$^{40}Ar/^{39}Ar$ Analytical Methods and Results

Sanidine and plagioclase were separated by standard mineral separation methods. This included coarse crushing and sieving the sample and then treating in dilute HF in and ultrasonic bath. Crystals were handpicked while the cleaned material was immersed in wintergreen oil viewed with a polarizing binocular microscope. Biotite grains were handpicked from the crushed material (not treated in HF) under a standard binocular microscope. The samples were irradiated in the NM-316 package for 16 hours at the USGS TRIGA reactor (central thimble) in Denver, CO along with the standard Fish Canyon tuff sanidine as a neutron flux monitor. Sanidine was analyzed by single crystal laser fusion (Tables 1, 2) using a CO_2 laser to heat the crystals whereas plagioclase single crystals were step-heated (Table 3) to produce low-resolution age spectra (generally 2-steps). Bulk samples of biotite were step-heated using a defocused diode laser to heat the samples (Tables 1, 3).

Eighteen single crystals of sanidine were dated from sample EGE_HOM_1 (Fig. 1). The dates range between about 18.1 to 17.4 Ma with 14 of the 18 dates yielding a weighted mean age of 18.057±0.009 Ma. The majority of crystals have K/Ca values of ~80 which is typical of sanidine, however the younger dates generally have much lower K/Ca values and associated low radiogenic yields (Fig. 1). Twenty-three plagioclase single crystals from EGE_HOM_1 yield concordant age spectra with plateau ages falling between 18.94±0.48 and 18.08±0.13 Ma (Fig. 2). The weighted mean of the plateau ages is 18.29±0.04 Ma (Table 1). Isochron analyses, which includes all 47 heating steps, gives an age of 18.21±0.04 Ma and a trapped initial $^{40}Ar/^{36}Ar$ value of 300±6 (Fig. 3a). EGE_HOM_1 biotite yields a generally climbing age spectrum with initial ages near 14 Ma that rise to about 18.6 Ma (Fig. 4). No plateau age is assigned to this sample

and the total gas age of ~17.1 Ma is substantially younger than the sanidine and plagioclase from this sample. For IT_BEMUT2, both plagioclase and biotite were dated. Twenty-three single plagioclase crystals were step-heated with 2 steps each and the spectra either reveal plateau ages or a discordant pattern where the initial step A is relatively old and the second step B is younger (Fig. 5). An isochron age of 17.17±0.09 Ma is obtained from all 46 heating steps with an initial trapped $^{40}Ar/^{36}Ar$ component of 361±6 that is substantially higher than modern atmosphere argon (Fig. 3b). Biotite for this sample yields a very anomalously young apparent age with many steps near 2 Ma and the highest temperature steps climbing to ca. 12 Ma (Fig. 4). Based on sample weight and ^{39}Ar production the sample appears to have about 1.5 weight % K_20 which is very low for pristine biotite (Table 2).

Discussion

The biotite data from this study did not yield meaningful age data as it appears the samples have undergone argon loss due to alteration.

The preferred eruption age for EGE_HOM_1 is given by the sanidine data at 18.057±0.009 Ma. The younger ages appear to come mainly from compositionally distinct grains that yield lower K/Ca values and are substantially less radiogenic compared to the grouping defining the preferred age. These grains are likely altered which can cause radiogenic argon loss and thus anomalously young ages (cf. Deino et al. 1990). The MSWD for the chosen population is somewhat high at 12.2 and may indicate geological scatter in the data. However, most sanidines dated at ultrahigh precision show some scatter of which at least partially can be attributed to neutron flux gradients where no two grains in the sample irradiation pit can share exactly equal geometry. Another source of scatter can be related to variable amounts of melt inclusions between the crystals and if the melt contains excess argon the distribution can show some dispersion. The plagioclase crystals have individual age spectra that are all concordant. A weighed mean of all 47 steps yields an age of 18.22±0.03 Ma with slight scatter (MSWD=3.38). The isochron data show similar scatter and yield an analytically indistinguishable age at 18.21±0.04 Ma. At two-sigma analytical uncertainty the sanidine and plagioclase results are slightly discordant. Because of the high precision and radiogenic yield of the sanidine data relative to the plagioclase data the preferred age of EGE_HOM_1 is given by the sanidine.

The plagioclase isochron age of 17.17±0.09 Ma is interpreted as the eruption of IT_BEMUT2. The individual age spectra commonly show an initial step that is old compared to the second step and also has lower radiogenic yield and thus support excess argon being the cause of the discordance. The isochron supports this, as the trapped initial $^{40}Ar/^{36}Ar$ is 361±6. The isochron does have some scatter as shown by the MSWD at 5.3. This likely is due to some variation of the trapped initial argon which is supported by some of the crystals having concordant age spectra which would be consistent with a more atmospheric initial $^{40}Ar/^{36}Ar$ value. There are various other ways to treat the plagioclase data. For instance, one could use only the B steps that are more precise and apparently less contaminated with excess argon and doing so yields an isochron age of 17.25±0.11 Ma. However, any reasonable combination of the data yields a similar age to that obtained by using all steps on a single isochron. Considering that there is only a small discordance between the sanidine and plagioclase from EGE_HOM_1, it may be likely that the plagioclase from IT_BEMUT2 is accurate and records a robust eruption age.

Palfy et al. (2007) published a zircon U/Pb age of 17.42±0.04 Ma and a $^{40}Ar/^{39}Ar$ plagioclase age of 17.02±0.14 Ma (both errors are 2σ). This argon age is based on a Fish Canyon standard age of 28.02 Ma and if normalized to the standard age of 28.201 Ma used here their age becomes 17.13 Ma which is essentially identical to the 17.17 Ma result from NM Tech. Palfy et al. (2007) suggested the zircon and plagioclase age discordance was due to either the calibration of the U/Pb and K/Ar systems or pre-eruptive residence of zircon in the magma. Renne et al. (2011) published an age of Fish Canyon sanidine of 28.294 Ma and a total decay constant of 5.5305x10-11 /a. Using this calibration the Palfy et al. (2007) plagioclase age becomes 17.21 Ma which is still much younger than the zircon age thereby supporting magma residence for the age discordance between the zircon and plagioclase. Because of magma residence issues for zircon, it appears the best eruption age is given by the $^{40}Ar/^{39}Ar$ results.

References Cited

Deino, A., and Potts, R., 1990, Single-crystal $^{40}Ar/^{39}Ar$ dating of the Olorgesailie Formation Southern Kenya Rift, J. Geophy. Res., 95, B6, 8453-8470.

Kuiper, K. F., Deino A., Hilgen, F. J., Krijgsman, W., Renne, P. R., and Wijbrans, J. R., 2008,

Synchronizing the rock clocks of Earth history. Science 320, 500–504.

Min, K., Mundil, R., Renne, P. R. and Ludwig, K. R., 2000, A test for systematic errors in $^{40}Ar/^{39}Ar$ geochronology through comparison with U–Pb analysis of a 1.1 Ga rhyolite. Geochim. Cosmochim. Acta 64, 73–98.

Palfy, J., Mundil, R., Renne, P.R., Bernor, R., Kordos, L. and Gasparik, M., 2007, U-Pb and $^{40}Ar/^{39}Ar$ dating of the Miocene fossil track site at Ipolytarnoc (Hungary) and it implications, Earth and Plant. Sci. Lett., 258, 160-174.

Renne, P.R., Balco, G, Ludwig, K.R., Mundil, R., and Min, K., 2011, Response to the comment by W.H. Schwarz et al. on “Joint determination of ^{40}K decay constants and $^{40}Ar^{*}/^{40}K$ for the Fish Canyon sanidine standard, and improved accuracy for $^{40}Ar/^{39}Ar$ geochronology” by P.R. Renne et al. (2010), Geochim. Cosmochim. Acta, 75, 5097-5100.

Steiger, R.H., and Jäger, E., 1977. Subcommission on geochronology: Convention on the use of decay constants in geo- and cosmochronology. Earth and Planet. Sci. Lett., 36, 359-362.

Taylor, J.R., 1982. An Introduction to Error Analysis: The Study of Uncertainties in Physical Measurements,. Univ. Sci. Books, Mill Valley, Calif., 270 p.

York, D., 1969. Least squares fitting of a straight line with correlated errors, Earth and Planet. Sci. Lett., 5, 320-324.

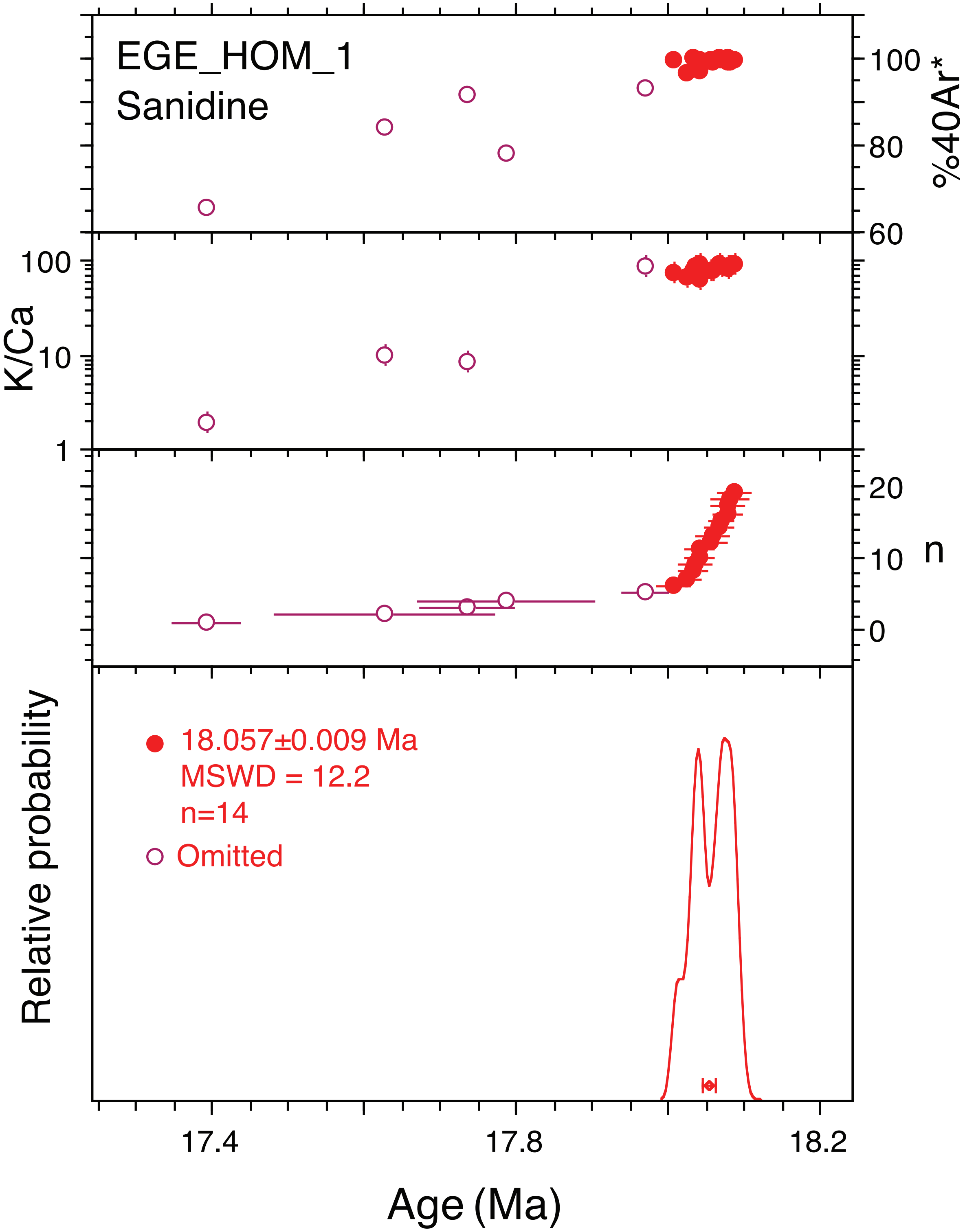

Figure 1. Age probability, K/Ca and radiogenic yield diagrams for single crystal sanidine total fusion results. The high radiogenic yield and high K/Ca results yield the preferred weighted mean age. The omitted data generally have low K/Ca and low radiogenic yield indicating a compositionally distinct population and apparent argon loss.

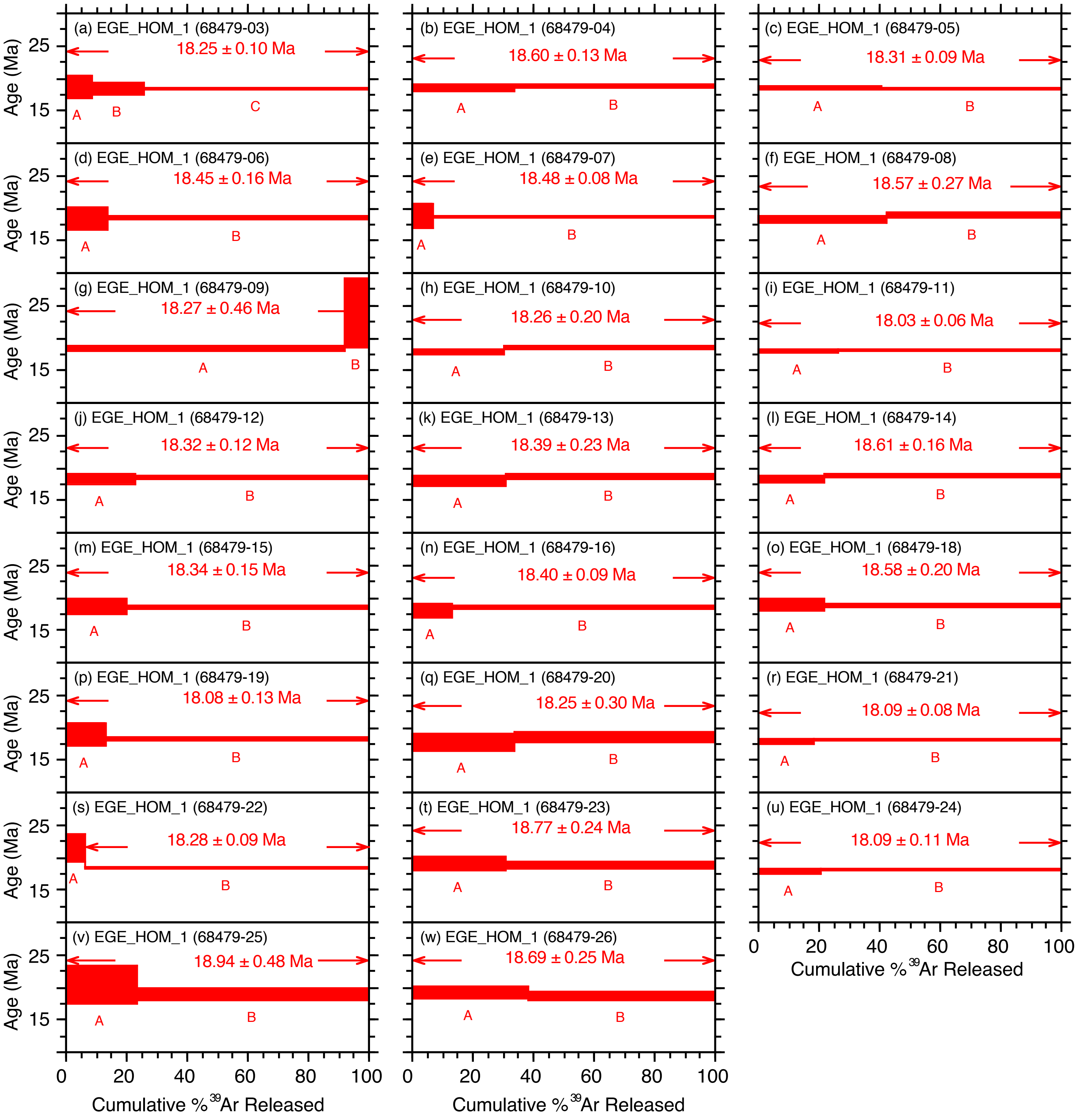

Figure 2. Age spectra for single crystal plagioclase step-heating results. All of the spectra yield concordant ages and the assigned ages represent the weight mean of the indicated steps.

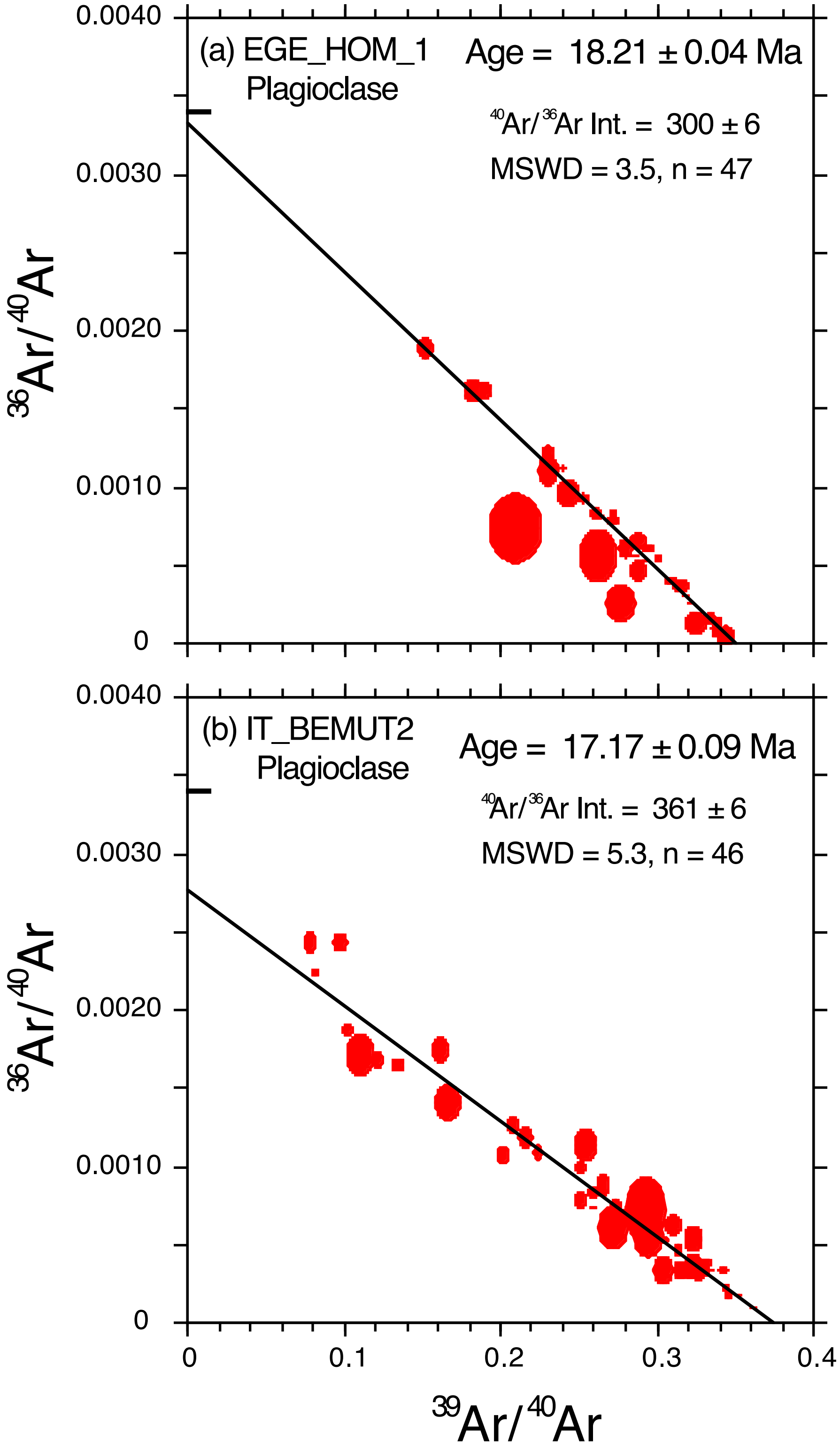

Figure 3. Isochron data derived for all heating steps of the plagioclase age spectrum analyses.

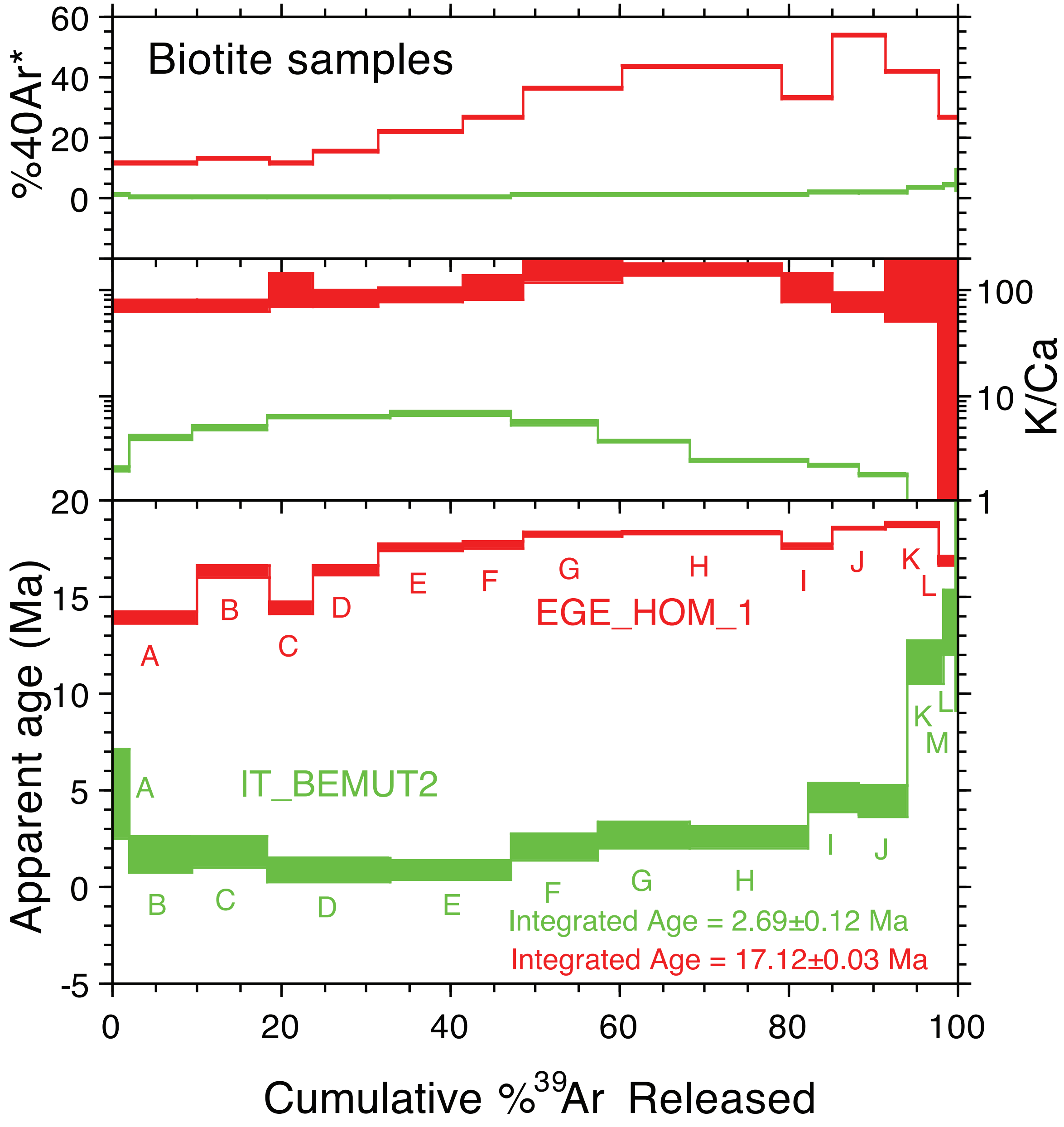

Figure 4. Age spectrum, K/Ca and radiogenic yield diagrams for step-heated bulk biotite samples. Both samples yield disturbed age spectra related to alteration that was not visibly apparent.

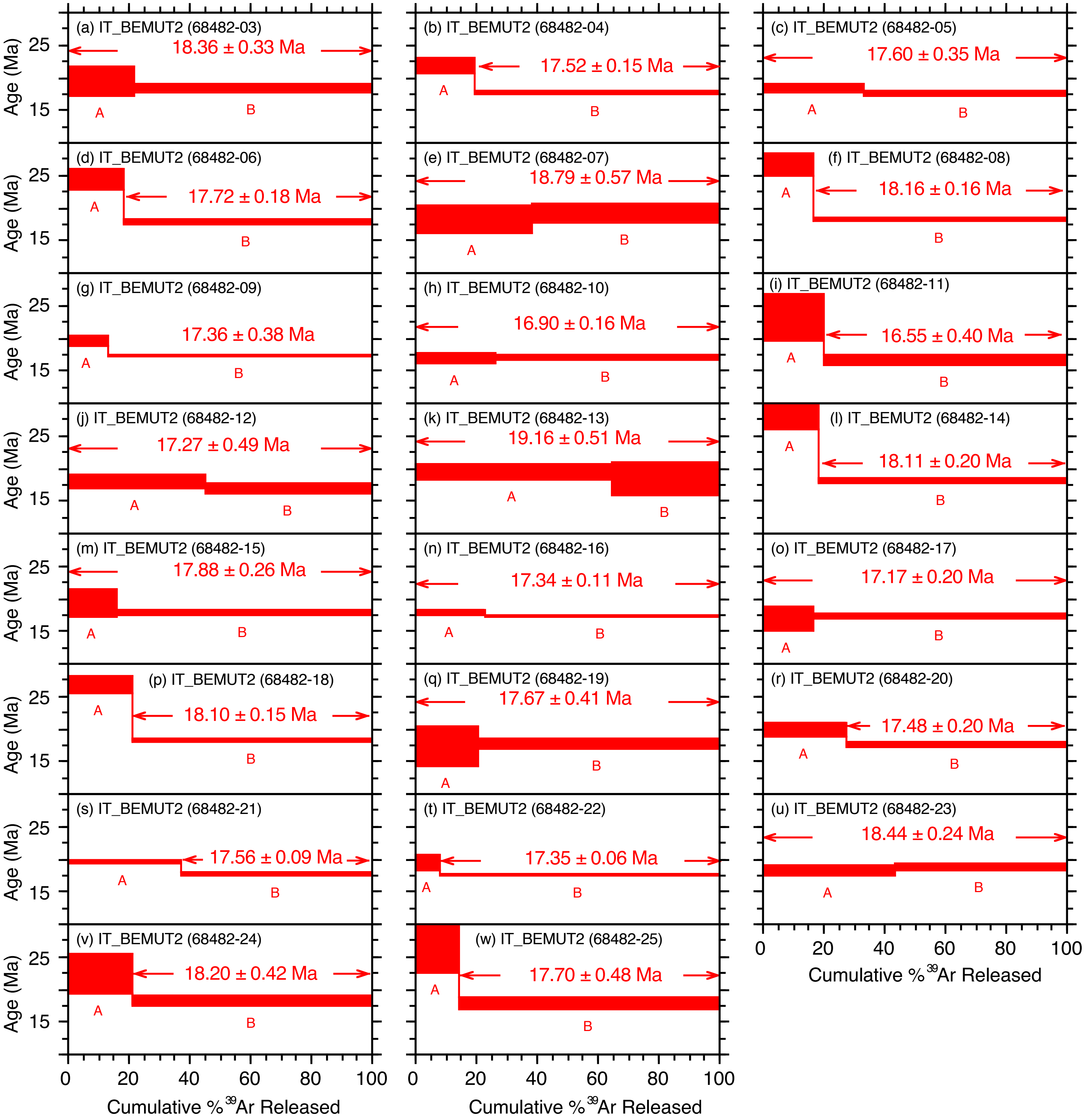

Figure 5. Age spectra for single crystal plagioclase step-heating results. Some of the spectra yield concordant ages whereas others have an older "A" step compared to the younger "B" step. For the concordant spectra the assigned ages represent the weight mean of the indicated steps. For the discordant spectra, the age shown is the measured age of the "B" step.

Table 1. 40Ar/39Ar analytical methods and age summary.

Summary

Sample	L#	Min	Weighted men				Isochron of all heating steps					
			Age	±	n	MSWD	Age	±	$^{40}Ar/^{36}Ar_o$	±	n	MSWD
EGE_HOM_1	68480	San	18.057	0.009	14	12.2						
EGE_HOM_1	68479	Plag					18.21	0.04	300	6	47	3.5
IT_BEMUT2	68482	Plag					17.17	0.09	361	6	46	5.3

L# = Lab number
n = number of heating steps or total fusions
Min = mineral. San = Sanidine, Plag = Plagioclase
All errors at 1σ

Methods

Sample preparation and irradiation:
Plagioclase and sanidine hand-picked under wintergreen oil using polarizing microscope.
Biotite handpicked from crushed rock.
Samples loaded into machined Al discs and irradiated for 16 hrs in the NM-316 irradiaiton package
in central thimble, USGS, Denver, CO.
Neutron flux monitor Fish Canyon Tuff sanidine (FC-2). Assigned age = 28.201 Ma Kuiper et al. (2008).

Instrumentation: Biotite
Biotite (bulk sample) step-heated using a 55 W Photon-Machines diode laser.
Helix MC plus mass spectrometer (system = Felix) on line with automated all-metal extraction system.
Multi-collector configuration: 40Ar-H2, 39Ar-H1, 38Ar-AX, 37Ar-L1, 36Ar-L2
Amplifier configuration: H2, L1 1E12; H1, 1E13; L1 1E14 Ohm Faradays, L2 - CDD ion counter, deadtime 20 ns.
Samples heated for 60 s.
Reactive gases removed by 1 minute exposure to one SAES GP-50 getter operated at ~450°C.
Gas also exposed to a cold finger operated at -140°C.

Instrumentation: Single crystal plagioclase and sanidine
Sanidine single crystals fused for 30 seconds using a 75 W Photon-Machines CO_2 laser.
Plagioclase single crystals step-heated (30 seconds per step) using a 75 W Photon-Machines CO_2 laser.
ARGUS VI mass spectrometer (system = Jan) on line with automated all-metal extraction system.
Multi-collector configuration: 40Ar-H1, 39Ar-Ax, 38Ar-L1, 37Ar-L2, 36Ar-L3
Amplifier configuation: H1, AX, 1E13; L1, L2, 1E14 Ohm Faradays, L3 - CDD ion counter, deadtime 14 ns.
Sanidine Samples fused for 30 seconds at 5 W using a CO2 laser.
Reactive gases removed by 30 second exposure to two NP-10 gettes, one operated at ~450°C and one at 20°C
and one GP-50 getter at 20°C.

Analytical parameters:
Mass spectrometer sensitivity $6x10^{-17}$ (Jan), $2.1x10^{-16}$ (Felix) moles/fA
Total system blank and background:
Felix = 10±4%, 0.5±12%, 0.064±70%, 0.1±85%, 0.05±3% x 10^{-17} moles for masses 40, 39, 38, 37, 36, respectively.
Jan = 8±1%, 0.17±20%, 0.07±10%, 0.14±7%, 0.04±3% x 10^{-17} moles for masses 40, 39, 38, 37, 36, respectively.
J-factors determined to a precision of ±0.03% by CO_2 laser-fusion of 6 single crystals
from 8 radial positions around the irradiation tray.

Table 2. Sanidine single crystal laser fusion argon data.

	ID	$^{40}Ar/^{39}Ar$	$^{37}Ar/^{39}Ar$	$^{36}Ar/^{39}Ar$ (x 10^{-3})	$^{39}Ar_K$ (x 10^{-15} mol)	K/Ca	$^{40}Ar^*$ (%)	Age (Ma)	±1σ (Ma)
	**EGE_HOM_1, Sanidine, J=0.0035174±0.03%, IC=0.990485±0.0008532, NM-316M, Lab#=68480**								
x	10	4.165	0.2808	4.964	6.989	1.8	65.3	17.394	0.033
x	06	3.287	0.0547	1.802	0.303	9.3	83.9	17.63	0.13
x	14	3.049	0.0638	0.9436	0.892	8.0	91.0	17.738	0.050
x	20	3.561	2.847	3.432	0.482	0.18	78.0	17.79	0.10
x	07	3.030	0.0061	0.7358	3.447	83.5	92.8	17.971	0.019
	04	2.838	0.0071	0.0672	8.403	71.4	99.3	18.011	0.007
	05	2.935	0.0081	0.3873	13.314	63.3	96.1	18.025	0.008
	13	2.833	0.0067	0.0353	9.585	75.7	99.6	18.035	0.006
	16	2.842	0.0062	0.0644	4.270	82.9	99.3	18.038	0.009
	03	2.847	0.0059	0.0784	11.349	87.1	99.2	18.043	0.006
	01	2.916	0.0083	0.3144	12.455	61.7	96.8	18.043	0.007
	11	2.842	0.0068	0.0538	6.014	74.7	99.5	18.059	0.007
	12	2.862	0.0067	0.1197	5.742	76.6	98.8	18.061	0.009
	02	2.838	0.0059	0.0348	6.057	86.9	99.7	18.068	0.007
	18	2.850	0.0061	0.0725	12.653	84.1	99.3	18.071	0.006
	08	2.862	0.0062	0.1081	9.928	81.8	98.9	18.081	0.006
	19	2.844	0.0066	0.0497	8.670	77.9	99.5	18.082	0.007
	17	2.872	0.0062	0.1434	3.871	83.0	98.5	18.084	0.011
	09	2.853	0.0059	0.0727	7.085	86.7	99.3	18.090	0.007
	**Mean age ± 1σ**	n=14		MSWD=12.2				18.057	0.009

Notes:

Isotopic ratios corrected for blank, radioactive decay, and mass discrimination, not corrected for interfering reactions.
Errors quoted for individual analyses include analytical error only, without interfering reaction or J uncertainties.
Mean age is weighted mean age of Taylor (1982). Mean age error is weighted error of the mean (Taylor, 1982), multiplied by the root of the MSWD where MSWD>1, and also incorporates uncertainty in J factors and irradiation correction uncertainties.
Isotopic abundances after Steiger and Jäger (1977).
x preceding sample ID denotes analyses excluded from mean age calculations.
Ages calculated relative to FC-2 Fish Canyon Tuff sanidine interlaboratory standard at 28.201 Ma
Decay Constant (LambdaK (total)) = 5.463e-10/a
Correction factors:

$(^{39}Ar/^{37}Ar)_{Ca}$ = 0.000682 ± 0.000004
$(^{36}Ar/^{37}Ar)_{Ca}$ = 0.0002761 ± 0.0000010
$(^{38}Ar/^{39}Ar)_{K}$ = 0.0124±0.0002
$(^{40}Ar/^{39}Ar)_{K}$ = 0.008 ± 0.0005

Table 3. Plagioclase and biotite step-heating data.

ID	Power (Watts)	$^{40}Ar/^{39}Ar$	$^{37}Ar/^{39}Ar$	$^{36}Ar/^{39}Ar$ (x 10^{-3})	$^{39}Ar_K$ (x 10^{-15} mol)	K/Ca	$^{40}Ar^*$ (%)	^{39}Ar (%)	Age (Ma)	±1σ (Ma)
**EGE_HOM_1, Plagioclase, J=0.0035076±0.03%, IC=0.9953±0.0008003, NM-316M, Lab#=68479-03**										
A	0.2	4.320	3.572	5.733	0.030	0.14	67.5	8.9	18.63	0.86
B	0.2	2.896	5.697	1.683	0.056	0.090	98.9	25.8	18.32	0.43
C	3.5	2.893	5.949	1.783	0.247	0.086	98.6	100.0	18.24	0.10
**Integrated age ± 1σ**		n=3			0.333	0.090			18.29	0.15
**Plateau ± 1σ** steps A-C		n=3	MSWD=0.11		0.333			100.0	18.25	0.10
**EGE_HOM_1, Plagioclase, J=0.0035076±0.03%, IC=0.9953±0.0008003, NM-316M, Lab#=68479-04**										
A	0.2	3.818	2.864	3.953	0.094	0.18	75.5	34.1	18.41	0.27
B	3.5	3.482	3.679	2.911	0.181	0.14	83.9	100.0	18.66	0.15
**Integrated age ± 1σ**		n=2			0.275	0.15			18.57	0.16
**Plateau ± 1σ** steps A-B		n=2	MSWD=0.67		0.275			100.0	18.60	0.13
**EGE_HOM_1, Plagioclase, J=0.0035076±0.03%, IC=0.9953±0.0008003, NM-316M, Lab#=68479-05**										
A	0.2	2.946	2.092	0.8217	0.169	0.24	97.6	40.7	18.34	0.15
B	3.5	2.957	3.680	1.326	0.246	0.14	96.9	100.0	18.30	0.11
**Integrated age ± 1σ**		n=2			0.415	0.17			18.315	0.110
**Plateau ± 1σ** steps A-B		n=2	MSWD=0.05		0.415			100.0	18.313	0.088
**EGE_HOM_1, Plagioclase, J=0.0035076±0.03%, IC=0.9953±0.0008003, NM-316M, Lab#=68479-06**										
A	0.2	5.443	2.736	9.478	0.030	0.19	52.6	14.1	18.29	0.90
B	3.5	3.787	3.774	4.082	0.181	0.14	76.2	100.0	18.45	0.16
**Integrated age ± 1σ**		n=2			0.211	0.14			18.43	0.20
**Plateau ± 1σ** steps A-B		n=2	MSWD=0.03		0.211			100.0	18.45	0.16
**EGE_HOM_1, Plagioclase, J=0.0035076±0.03%, IC=0.9953±0.0008003, NM-316M, Lab#=68479-07**										
A	0.2	6.525	2.652	12.97	0.027	0.19	44.5	7.1	18.56	0.93
B	3.5	3.409	3.502	2.708	0.358	0.15	84.9	100.0	18.482	0.080
**Integrated age ± 1σ**		n=2			0.385	0.15			18.488	0.120
**Plateau ± 1σ** steps A-B		n=2	MSWD=0.01		0.385			100.0	18.483	0.080
**EGE_HOM_1, Plagioclase, J=0.0035076±0.03%, IC=0.9953±0.0008003, NM-316M, Lab#=68479-08**										
A	0.2	2.988	4.540	1.719	0.091	0.11	95.4	42.7	18.22	0.25
B	3.5	3.088	5.243	1.963	0.122	0.097	95.1	100.0	18.77	0.20
**Integrated age ± 1σ**		n=2			0.213	0.10			18.53	0.19
**Plateau ± 1σ** steps A-B		n=2	MSWD=2.98		0.213			100.0	18.57	0.27
**EGE_HOM_1, Plagioclase, J=0.0035076±0.03%, IC=0.9953±0.0008003, NM-316M, Lab#=68479-09**										
A	0.2	3.229	2.960	2.076	0.104	0.17	88.5	92.3	18.24	0.22
B	3.5	4.735	2.730	4.170	0.009	0.19	78.6	100.0	23.8	2.7
**Integrated age ± 1σ**		n=2			0.113	0.17			18.66	0.30
**Plateau ± 1σ** steps A-B		n=2	MSWD=4.26		0.113			100.0	18.27	0.46

Table 3. Plagioclase and biotite step-heating data.

ID	Power (Watts)	$^{40}Ar/^{39}Ar$	$^{37}Ar/^{39}Ar$	$^{36}Ar/^{39}Ar$ (x 10^{-3})	$^{39}Ar_K$ (x 10^{-15} mol)	K/Ca	$^{40}Ar^*$ (%)	^{39}Ar (%)	Age (Ma)	±1σ (Ma)
**EGE_HOM_1, Plagioclase, J=0.0035076±0.03%, IC=0.9953±0.0008003, NM-316M, Lab#=68479-10**										
A	0.2	3.322	2.108	2.370	0.117	0.24	84.1	30.6	17.83	0.19
B	3.5	3.099	2.789	1.524	0.266	0.18	92.8	100.0	18.356	0.090
**Integrated age ± 1σ**		n=2			0.383	0.20			18.194	0.110
**Plateau ± 1σ** steps A-B		n=2	MSWD=6.09		0.383			100.0	18.26	0.20
**EGE_HOM_1, Plagioclase, J=0.0035076±0.03%, IC=0.9953±0.0008003, NM-316M, Lab#=68479-11**										
A	0.2	4.161	1.381	4.998	0.172	0.37	67.2	26.6	17.84	0.15
B	3.5	2.875	2.400	0.8167	0.473	0.21	98.4	100.0	18.054	0.048
**Integrated age ± 1σ**		n=2			0.645	0.24			17.997	0.070
**Plateau ± 1σ** steps A-B		n=2	MSWD=1.82		0.645			100.0	18.034	0.062
**EGE_HOM_1, Plagioclase, J=0.0035076±0.03%, IC=0.9953±0.0008003, NM-316M, Lab#=68479-12**										
A	0.2	3.160	5.712	2.691	0.055	0.089	89.6	23.0	18.11	0.39
B	3.5	2.948	5.467	1.781	0.184	0.093	97.3	100.0	18.34	0.12
**Integrated age ± 1σ**		n=2			0.239	0.092			18.29	0.16
**Plateau ± 1σ** steps A-B		n=2	MSWD=0.32		0.239			100.0	18.32	0.12
**EGE_HOM_1, Plagioclase, J=0.0035076±0.03%, IC=0.9953±0.0008003, NM-316M, Lab#=68479-13**										
A	0.2	3.460	2.796	2.994	0.049	0.18	81.0	30.9	17.90	0.42
B	3.5	3.008	7.700	2.532	0.110	0.066	96.0	100.0	18.50	0.20
**Integrated age ± 1σ**		n=2			0.159	0.082			18.31	0.20
**Plateau ± 1σ** steps A-B		n=2	MSWD=1.62		0.159			100.0	18.39	0.23
**EGE_HOM_1, Plagioclase, J=0.0035076±0.03%, IC=0.9953±0.0008003, NM-316M, Lab#=68479-14**										
A	0.2	3.670	3.143	3.693	0.078	0.16	77.2	22.2	18.10	0.29
B	3.5	3.076	5.183	1.963	0.272	0.098	94.9	100.0	18.663	0.090
**Integrated age ± 1σ**		n=2			0.350	0.11			18.538	0.120
**Plateau ± 1σ** steps A-B		n=2	MSWD=3.45		0.350			100.0	18.61	0.16
**EGE_HOM_1, Plagioclase, J=0.0035076±0.03%, IC=0.9953±0.0008003, NM-316M, Lab#=68479-15**										
A	0.2	4.311	1.365	5.170	0.036	0.37	67.1	20.2	18.46	0.63
B	3.5	3.001	3.247	1.339	0.142	0.16	95.6	100.0	18.33	0.16
**Integrated age ± 1σ**		n=2			0.177	0.18			18.36	0.18
**Plateau ± 1σ** steps A-B		n=2	MSWD=0.04		0.177			100.0	18.34	0.15
**EGE_HOM_1, Plagioclase, J=0.0035076±0.03%, IC=0.9953±0.0008003, NM-316M, Lab#=68479-16**										
A	0.2	4.337	2.807	5.988	0.045	0.18	64.4	13.3	17.85	0.51
B	3.5	2.944	5.781	1.818	0.295	0.088	97.8	100.0	18.413	0.086
**Integrated age ± 1σ**		n=2			0.341	0.095			18.34	0.12
**Plateau ± 1σ** steps A-B		n=2	MSWD=1.18		0.341			100.0	18.397	0.092
**EGE_HOM_1, Plagioclase, J=0.0035076±0.03%, IC=0.9953±0.0008003, NM-316M, Lab#=68479-18**										
A	0.2	3.566	3.783	3.184	0.048	0.13	82.2	21.8	18.74	0.50
B	3.5	2.955	5.115	1.584	0.170	0.100	98.3	100.0	18.56	0.15
**Integrated age ± 1σ**		n=2			0.218	0.11			18.60	0.19
**Plateau ± 1σ** steps A-B		n=2	MSWD=0.12		0.218			100.0	18.58	0.20

Table 3. Plagioclase and biotite step-heating data.

	ID	Power (Watts)	$^{40}Ar/^{39}Ar$	$^{37}Ar/^{39}Ar$	$^{36}Ar/^{39}Ar$ (x 10^{-3})	$^{39}Ar_K$ (x 10^{-15} mol)	K/Ca	$^{40}Ar^*$ (%)	^{39}Ar (%)	Age (Ma)	±1σ (Ma)
	**EGE_HOM_1, Plagioclase, J=0.0035076±0.03%, IC=0.9953±0.0008003, NM-316M, Lab#=68479-19**										
	A	0.2	4.094	2.837	4.652	0.028	0.18	72.0	13.5	18.83	0.86
	B	3.5	2.947	3.760	1.440	0.179	0.14	96.0	100.0	18.06	0.13
	**Integrated age ± 1σ**		n=2			0.207	0.14			18.17	0.20
	**Plateau ± 1σ** steps A-B		n=2	MSWD=0.79		0.207			100.0	18.08	0.13
	**EGE_HOM_1, Plagioclase, J=0.0035076±0.03%, IC=0.9953±0.0008003, NM-316M, Lab#=68479-20**										
	A	0.2	5.230	3.989	9.458	0.034	0.13	52.7	33.9	17.64	0.69
	B	3.5	3.944	3.945	4.686	0.067	0.13	73.0	100.0	18.40	0.34
	**Integrated age ± 1σ**		n=2			0.101	0.13			18.14	0.40
	**Plateau ± 1σ** steps A-B		n=2	MSWD=0.99		0.101			100.0	18.25	0.30
	**EGE_HOM_1, Plagioclase, J=0.0035076±0.03%, IC=0.9953±0.0008003, NM-316M, Lab#=68479-21**										
	A	0.2	3.681	1.003	3.308	0.134	0.51	75.6	18.5	17.76	0.17
	B	3.5	2.952	2.385	1.047	0.589	0.21	96.1	100.0	18.109	0.042
	**Integrated age ± 1σ**		n=2			0.724	0.24			18.045	0.060
	**Plateau ± 1σ** steps A-B		n=2	MSWD=3.73		0.724			100.0	18.089	0.080
	**EGE_HOM_1, Plagioclase, J=0.0035076±0.03%, IC=0.9953±0.0008003, NM-316M, Lab#=68479-22**										
x	A	0.2	3.605	4.809	2.218	0.020	0.11	92.7	6.5	21.4	1.1
	B	3.5	3.136	4.948	2.300	0.296	0.10	91.2	100.0	18.281	0.087
	**Integrated age ± 1σ**		n=2			0.317	0.10			18.48	0.12
	**Step B**									18.281	0.087
	**EGE_HOM_1, Plagioclase, J=0.0035076±0.03%, IC=0.9953±0.0008003, NM-316M, Lab#=68479-23**										
	A	0.2	3.062	5.632	1.900	0.038	0.091	96.7	31.3	18.93	0.55
	B	3.5	3.072	4.762	1.792	0.083	0.11	95.4	100.0	18.73	0.26
	**Integrated age ± 1σ**		n=2			0.121	0.10			18.79	0.30
	**Plateau ± 1σ** steps A-B		n=2	MSWD=0.11		0.121			100.0	18.77	0.24
	**EGE_HOM_1, Plagioclase, J=0.0035076±0.03%, IC=0.9953±0.0008003, NM-316M, Lab#=68479-24**										
	A	0.2	3.384	1.258	2.400	0.149	0.41	82.0	21.1	17.72	0.16
	B	3.5	2.876	2.857	0.9180	0.557	0.18	98.7	100.0	18.118	0.045
	**Integrated age ± 1σ**		n=2			0.706	0.20			18.033	0.060
	**Plateau ± 1σ** steps A-B		n=2	MSWD=5.93		0.706			100.0	18.09	0.11
	**EGE_HOM_1, Plagioclase, J=0.0035076±0.03%, IC=0.9953±0.0008003, NM-316M, Lab#=68479-25**										
	A	0.2	3.804	1.777	2.607	0.015	0.29	83.5	23.8	20.3	1.5
	B	3.5	4.343	5.202	6.194	0.048	0.098	67.6	100.0	18.78	0.51
	**Integrated age ± 1σ**		n=2			0.063	0.12			19.13	0.60
	**Plateau ± 1σ** steps A-B		n=2	MSWD=0.93		0.063			100.0	18.94	0.48
	**EGE_HOM_1, Plagioclase, J=0.0035076±0.03%, IC=0.9953±0.0008003, NM-316M, Lab#=68479-26**										
	A	0.2	3.464	0.9870	1.874	0.052	0.52	86.3	38.7	19.07	0.47
	B	3.5	2.904	5.432	1.515	0.083	0.094	99.9	100.0	18.54	0.30
	**Integrated age ± 1σ**		n=2			0.135	0.14			18.74	0.03
	**Plateau ± 1σ** steps A-B		n=2	MSWD=0.91		0.135			100.0	18.69	0.25

Table 3. Plagioclase and biotite step-heating data.

	ID	Power (Watts)	$^{40}Ar/^{39}Ar$	$^{37}Ar/^{39}Ar$	$^{36}Ar/^{39}Ar$ (x 10^{-3})	$^{39}Ar_K$ (x 10^{-15} mol)	K/Ca	$^{40}Ar^*$ (%)	^{39}Ar (%)	Age (Ma)	±1σ (Ma)
	**IT_BEMUT2, Plagioclase, J=0.0035237±0.03%, IC=0.9953±0.0008003, NM-316M, Lab#=68482-03**										
	A	0.2	3.655	6.348	3.930	0.021	0.080	82.3	22.0	19.4	1.1
	B	3.5	3.628	6.506	4.458	0.075	0.078	78.3	100.0	18.27	0.34
	**Integrated age ± 1σ**			n=2		0.096	0.079			18.50	0.37
	**Plateau ± 1σ** steps A-B			n=2	MSWD=0.82	0.096			100.0	18.36	0.33
	**IT_BEMUT2, Plagioclase, J=0.0035237±0.03%, IC=0.9953±0.0008003, NM-316M, Lab#=68482-04**										
x	A	0.2	4.938	2.620	5.991	0.042	0.19	68.4	19.9	21.67	0.61
	B	3.5	3.009	5.740	2.541	0.169	0.089	90.6	100.0	17.52	0.15
	**Integrated age ± 1σ**			n=2		0.211	0.100			18.35	0.17
	**Step B**									17.52	0.15
	**IT_BEMUT2, Plagioclase, J=0.0035237±0.03%, IC=0.9953±0.0008003, NM-316M, Lab#=68482-05**										
	A	0.2	3.162	4.771	2.375	0.063	0.11	90.1	33.3	18.30	0.38
	B	3.5	3.000	10.50	3.902	0.127	0.049	90.1	100.0	17.43	0.19
	**Integrated age ± 1σ**			n=2		0.190	0.059			17.72	0.18
	**Plateau ± 1σ** steps A-B			n=2	MSWD=4.30	0.190			100.0	17.60	0.35
	**IT_BEMUT2, Plagioclase, J=0.0035237±0.03%, IC=0.9953±0.0008003, NM-316M, Lab#=68482-06**										
x	A	0.2	7.351	3.962	13.15	0.033	0.13	51.5	18.5	24.29	0.76
	B	3.5	3.379	9.835	4.846	0.146	0.052	81.3	100.0	17.72	0.18
	**Integrated age ± 1σ**			n=2		0.179	0.058			18.94	0.20
	**Step B**									17.72	0.18
	**IT_BEMUT2, Plagioclase, J=0.0035237±0.03%, IC=0.9953±0.0008003, NM-316M, Lab#=68482-07**										
	A	0.2	3.376	10.32	4.726	0.021	0.049	83.5	38.5	18.2	1.0
	B	3.5	3.272	11.25	4.180	0.033	0.045	90.3	100.0	19.05	0.68
	**Integrated age ± 1σ**			n=2		0.054	0.047			18.72	0.68
	**Plateau ± 1σ** steps A-B			n=2	MSWD=0.48	0.054			100.0	18.79	0.57
	**IT_BEMUT2, Plagioclase, J=0.0035237±0.03%, IC=0.9953±0.0008003, NM-316M, Lab#=68482-08**										
x	A	0.2	8.169	5.101	15.11	0.032	0.10	50.4	16.9	26.42	0.87
	B	3.5	3.089	8.804	3.341	0.159	0.058	91.3	100.0	18.16	0.16
	**Integrated age ± 1σ**			n=2		0.191	0.062			19.56	0.19
	**Step B**									18.16	0.16
	**IT_BEMUT2, Plagioclase, J=0.0035237±0.03%, IC=0.9953±0.0008003, NM-316M, Lab#=68482-09**										
	A	0.2	4.464	2.398	5.493	0.056	0.21	68.0	13.3	19.46	0.45
	B	3.5	2.974	5.252	2.404	0.362	0.097	90.5	100.0	17.296	0.081
	**Integrated age ± 1σ**			n=2		0.418	0.10			17.585	0.110
	**Plateau ± 1σ** steps A-B			n=2	MSWD=22.2(	0.418			100.0	17.36	0.38
	**IT_BEMUT2, Plagioclase, J=0.0035237±0.03%, IC=0.9953±0.0008003, NM-316M, Lab#=68482-10**										
x	A	0.2	3.204	5.179	3.418	0.062	0.099	81.6	26.6	16.81	0.41
	B	3.5	2.904	6.214	2.649	0.170	0.082	90.5	100.0	16.90	0.16
	**Integrated age ± 1σ**			n=2		0.232	0.086			16.87	0.19
	**Step B**									16.90	0.16

Table 3. Plagioclase and biotite step-heating data.

	ID	Power	$^{40}Ar/^{39}Ar$	$^{37}Ar/^{39}Ar$	$^{36}Ar/^{39}Ar$	$^{39}Ar_K$	K/Ca	$^{40}Ar^*$	^{39}Ar	Age	±1σ
		(Watts)			(x 10^{-3})	(x 10^{-15} mol)		(%)	(%)	(Ma)	(Ma)
	**IT_BEMUT2, Plagioclase, J=0.0035237±0.03%, IC=0.9953±0.0008003, NM-316M, Lab#=68482-11**										
x	A	0.2	12.70	3.177	31.72	0.016	0.16	28.2	20.3	23.0	1.8
	B	3.5	3.068	9.024	4.180	0.061	0.057	83.7	100.0	16.55	0.40
	**Integrated age ± 1σ**			n=2		0.077	0.065			17.86	0.58
	**Step B**									16.55	0.40
	**IT_BEMUT2, Plagioclase, J=0.0035237±0.03%, IC=0.9953±0.0008003, NM-316M, Lab#=68482-12**										
	A	0.2	3.759	2.605	4.002	0.046	0.20	74.2	45.2	17.89	0.51
	B	3.5	3.080	5.868	3.155	0.055	0.087	85.2	100.0	16.88	0.41
	**Integrated age ± 1σ**			n=2		0.101	0.12			17.33	0.38
	**Plateau ± 1σ** steps A-B			n=2	MSWD=2.36	0.101			100.0	17.27	0.49
	**IT_BEMUT2, Plagioclase, J=0.0035237±0.03%, IC=0.9953±0.0008003, NM-316M, Lab#=68482-13**										
	A	0.2	4.776	8.621	8.369	0.044	0.059	62.9	65.0	19.35	0.56
	B	3.5	10.10	2.077	25.12	0.024	0.25	28.1	100.0	18.3	1.2
	**Integrated age ± 1σ**			n=2		0.068	0.081			18.97	0.68
	**Plateau ± 1σ** steps A-B			n=2	MSWD=0.66	0.068			100.0	19.16	0.51
	**IT_BEMUT2, Plagioclase, J=0.0035237±0.03%, IC=0.9953±0.0008003, NM-316M, Lab#=68482-14**										
x	A	0.2	9.705	2.949	18.92	0.028	0.17	44.8	18.6	27.9	1.0
	B	3.5	3.087	11.06	3.996	0.122	0.046	90.9	100.0	18.11	0.20
	**Integrated age ± 1σ**			n=2		0.149	0.053			19.93	2.26
	**Step B**									18.11	0.20
	**IT_BEMUT2, Plagioclase, J=0.0035237±0.03%, IC=0.9953±0.0008003, NM-316M, Lab#=68482-15**										
	A	0.2	6.121	10.18	13.46	0.024	0.050	48.5	16.3	19.2	1.0
	B	3.5	3.256	13.17	5.314	0.122	0.039	84.7	100.0	17.83	0.21
	**Integrated age ± 1σ**			n=2		0.146	0.040			18.05	0.27
	**Plateau ± 1σ** steps A-B			n=2	MSWD=1.67	0.146			100.0	17.88	0.26
	**IT_BEMUT2, Plagioclase, J=0.0035237±0.03%, IC=0.9953±0.0008003, NM-316M, Lab#=68482-16**										
	A	0.2	3.190	2.510	2.155	0.099	0.20	86.4	22.9	17.69	0.24
	B	3.5	2.761	5.760	1.821	0.334	0.089	97.5	100.0	17.305	0.079
	**Integrated age ± 1σ**			n=2		0.433	0.10			17.392	0.100
	**Plateau ± 1σ** steps A-B			n=2	MSWD=2.29	0.433			100.0	17.34	0.11
	**IT_BEMUT2, Plagioclase, J=0.0035237±0.03%, IC=0.9953±0.0008003, NM-316M, Lab#=68482-17**										
	A	0.2	3.910	8.053	6.609	0.025	0.063	66.8	16.6	16.82	0.94
	B	3.5	3.000	8.603	3.493	0.125	0.059	89.0	100.0	17.19	0.21
	**Integrated age ± 1σ**			n=2		0.149	0.060			17.13	2.27
	**Plateau ± 1σ** steps A-B			n=2	MSWD=0.14	0.149			100.0	17.17	0.20
	**IT_BEMUT2, Plagioclase, J=0.0035237±0.03%, IC=0.9953±0.0008003, NM-316M, Lab#=68482-18**										
x	A	0.2	12.12	4.308	28.20	0.053	0.12	34.1	21.7	26.57	0.66
	B	3.5	3.552	9.123	5.027	0.190	0.056	79.1	100.0	18.10	0.15
	**Integrated age ± 1σ**			n=2		0.243	0.063			19.94	0.18
	**Step B**									18.10	0.15

Table 3. Plagioclase and biotite step-heating data.

	ID	Power (Watts)	$^{40}Ar/^{39}Ar$	$^{37}Ar/^{39}Ar$	$^{36}Ar/^{39}Ar$ (x 10^{-3})	$^{39}Ar_K$ (x 10^{-15} mol)	K/Ca	$^{40}Ar^*$ (%)	^{39}Ar (%)	Age (Ma)	±1σ (Ma)
	**IT_BEMUT2, Plagioclase, J=0.0035237±0.03%, IC=0.9953±0.0008003, NM-316M, Lab#=68482-19**										
x	A	0.2	3.393	9.883	5.148	0.016	0.052	78.9	20.7	17.3	1.5
	B	3.5	3.041	10.54	3.928	0.062	0.048	90.1	100.0	17.67	0.41
	**Integrated age ± 1σ**			n=2		0.078	0.049			17.58	0.59
	**Step B**									17.67	0.41
	**IT_BEMUT2, Plagioclase, J=0.0035237±0.03%, IC=0.9953±0.0008003, NM-316M, Lab#=68482-20**										
x	A	0.2	3.974	3.560	4.055	0.049	0.14	77.1	27.9	19.67	0.51
	B	3.5	2.897	7.405	2.655	0.126	0.069	93.8	100.0	17.48	0.20
	**Integrated age ± 1σ**			n=2		0.174	0.081			18.09	0.20
	**Step B**									17.48	0.20
	**IT_BEMUT2, Plagioclase, J=0.0035237±0.03%, IC=0.9953±0.0008003, NM-316M, Lab#=68482-21**										
x	A	0.2	3.846	1.180	3.103	0.168	0.43	78.7	37.5	19.38	0.15
	B	3.5	2.878	5.616	2.043	0.280	0.091	94.9	100.0	17.557	0.091
	**Integrated age ± 1σ**			n=2		0.448	0.13			18.24	0.10
	**Step B**									17.557	0.091
	**IT_BEMUT2, Plagioclase, J=0.0035237±0.03%, IC=0.9953±0.0008003, NM-316M, Lab#=68482-22**										
x	A	0.2	4.614	3.452	6.400	0.037	0.15	65.1	8.0	19.27	0.63
	B	3.5	2.834	6.717	2.311	0.422	0.076	95.2	100.0	17.353	0.064
	**Integrated age ± 1σ**			n=2		0.459	0.079			17.508	0.087
	**Step B**									17.353	0.064
	**IT_BEMUT2, Plagioclase, J=0.0035237±0.03%, IC=0.9953±0.0008003, NM-316M, Lab#=68482-23**										
	A	0.2	3.963	8.976	6.343	0.064	0.057	71.1	43.9	18.17	0.38
	B	3.5	3.824	9.859	5.883	0.081	0.052	75.5	100.0	18.62	0.31
	**Integrated age ± 1σ**			n=2		0.145	0.054			18.42	0.26
	**Plateau ± 1σ** steps A-B			n=2	MSWD=0.88	0.145			100.0	18.44	0.24
	**IT_BEMUT2, Plagioclase, J=0.0035237±0.03%, IC=0.9953±0.0008003, NM-316M, Lab#=68482-24**										
x	A	0.2	5.939	7.082	10.28	0.016	0.072	58.5	21.5	22.4	1.5
	B	3.5	3.424	10.81	5.019	0.057	0.047	82.4	100.0	18.20	0.42
	**Integrated age ± 1σ**			n=2		0.073	0.051			19.09	0.56
	**Step B**									18.20	0.42
	**IT_BEMUT2, Plagioclase, J=0.0035237±0.03%, IC=0.9953±0.0008003, NM-316M, Lab#=68482-25**										
x	A	0.2	8.983	4.555	16.60	0.009	0.11	49.5	14.6	28.5	3.1
	B	3.5	3.067	12.27	4.490	0.052	0.042	89.4	100.0	17.70	0.48
	**Integrated age ± 1σ**			n=2		0.061	0.046			19.28	0.66
	**Step B**									17.70	0.48

Table 3. Plagioclase and biotite step-heating data.

ID	Power (Watts)	$^{40}Ar/^{39}Ar$	$^{37}Ar/^{39}Ar$	$^{36}Ar/^{39}Ar$ (x 10^{-3})	$^{39}Ar_K$ (x 10^{-15} mol)	K/Ca	$^{40}Ar^*$ (%)	^{39}Ar (%)	Age (Ma)	±1σ (Ma)
**EGE_HOM_1, Biotite, 3.02 mg, J=0.0035209±0.03%, IC=1.001698±0.0009091, NM-316M, Lab#=68481-01Helix MC**										
A	0.5	18.64	0.0073	55.74	18.7	70.1	11.6	10.2	13.87	0.13
B	0.8	19.89	0.0073	58.73	15.8	70.1	12.7	18.8	16.24	0.13
C	1.0	19.32	0.0050	57.81	9.2	102.9	11.6	23.8	14.33	0.15
D	1.5	16.35	0.0061	46.72	14.5	84.0	15.5	31.7	16.27	0.12
E	1.9	12.46	0.0057	32.95	18.4	89.3	21.8	41.7	17.458	0.088
F	2.1	10.35	0.0049	25.71	12.9	104.9	26.6	48.7	17.620	0.090
G	2.5	7.897	0.0034	17.11	21.3	151.2	35.9	60.3	18.167	0.055
H	3.5	6.627	0.0033	12.76	34.6	154.6	43.1	79.2	18.279	0.037
I	4.0	8.396	0.0046	19.12	11.3	110.8	32.6	85.4	17.562	0.076
J	5.0	5.410	0.0066	8.528	11.6	77.3	53.4	91.7	18.487	0.048
K	7.0	6.991	0.0017	13.78	11.6	299.3	41.7	98.0	18.668	0.062
L	12.0	10.02	0.0024	24.98	3.7	210.9	26.2	100.0	16.85	0.13
**Integrated age ± 1σ**			n=12		183.6	104.5	K2O=6.63%		16.872	0.027
**No Plateau**										
**IT_BEMUT2, Biotite, 3.12 mg, J=0.0035302±0.03%, IC=0.9960785±0.000708, NM-316M, Lab#=68483-01Helix MC**										
A	0.5	98.37	0.2609	330.4	0.9	2.0	0.8	2.1	4.8	1.2
B	0.8	57.16	0.1308	192.6	3.2	3.9	0.5	9.6	1.67	0.46
C	1.0	52.56	0.1059	177.0	3.8	4.8	0.5	18.6	1.75	0.43
D	1.5	45.52	0.0858	153.6	6.1	5.9	0.3	32.9	0.79	0.32
E	1.9	40.91	0.0779	138.0	6.2	6.5	0.3	47.4	0.81	0.26
F	2.1	40.22	0.0955	135.1	4.4	5.3	0.8	57.6	2.01	0.33
G	2.5	41.29	0.1404	138.4	4.6	3.6	1.0	68.4	2.61	0.33
H	3.5	40.90	0.2135	137.1	5.9	2.4	1.0	82.3	2.52	0.30
I	4.0	39.64	0.2404	131.8	2.6	2.1	1.8	88.3	4.55	0.39
J	5.0	39.43	0.2918	131.2	2.5	1.7	1.7	94.2	4.34	0.41
K	7.0	55.52	1.182	182.1	1.8	0.43	3.2	98.5	11.56	0.53
L	12.0	56.59	2.121	184.9	0.6	0.24	3.7	100.0	13.62	0.85
**Integrated age ± 1σ**			n=12		42.8	2.4	K2O=1.49%		2.63	0.11
**No Plateau**										

Notes:

Isotopic ratios corrected for blank, radioactive decay, and mass discrimination, not corrected for interfering reactions.
Errors quoted for individual analyses include analytical error only, without interfering reaction or J uncertainties.
Integrated age calculated by summing isotopic measurements of all steps.
Integrated age error calculated by quadratically combining errors of isotopic measurements of all steps.
Plateau age is inverse-variance-weighted mean of selected steps.
Plateau age error is inverse-variance-weighted mean error (Taylor, 1982) times root MSWD where MSWD>1.
Plateau error is weighted error of Taylor (1982).
Decay constants and isotopic abundances after Steiger and Jäger (1977).
symbol preceding sample ID denotes analyses excluded from plateau age calculations.
Ages calculated relative to FC-2 Fish Canyon Tuff sanidine interlaboratory standard at 28.201 Ma
Decay Constant (LambdaK (total)) = 5.463e-10/a
Correction factors:

$(^{39}Ar/^{37}Ar)_{Ca}$ = 0.000682 ± 0.000004
$(^{36}Ar/^{37}Ar)_{Ca}$ = 0.0002761 ± 0.0000010
$(^{38}Ar/^{39}Ar)_K$ = 0.0124±0.00002
$(^{40}Ar/^{39}Ar)_K$ = 0.008 ± 0.0005
