# Supplementary material for: Large-magnitude (VEI ≥ 7) ‘wet’ explosive silicic eruption preserved a Lower Miocene habitat at the Ipolytarnóc Fossil Site, North Hungary
Source: Sci Rep. 2022 Jun 13;12:9743. doi: 10.1038/s41598-022-13586-3 (PMC9192734; doi:10.1038/s41598-022-13586-3)
Supplement: Supplementary file 12 — Supplementary Information 5. [file 41598_2022_13586_MOESM12_ESM.docx]

**Volume estimation of the Eger-Ipolytarnóc Ignimbrite**

On the basis of the verified surface occurrences of the Eger-Ipolytarnóc Ignimbrite, the present-day spatial distribution and the volume of the pyroclastic deposits can be estimated. Notably, the ignimbrite, which is the deposit of the final phase (Unit D) of the eruption, is by far the largest in volume.

In the following, we give a GIS-based calculation, which is considered as a minimum/conservative estimate due to a number of factors. However, at first, we address the issues of (1) source vent, (2) possible distribution of the ignimbrite, (3) interpretation of preserved thickness, (4) role of postvolcanic basin subsidence.

(1) The inferred source vent of the North Hungarian ignimbrites has long been assumed to be southeast of the Bükk Foreland, within the Vatta-Maklár trench, as site of a caldera cluster^1^ (Fig. 1B in main text). Since the vent area has been subsided since Miocene times, we do not have more data on its character, and a fissure-type vent system^2^ is also plausible. Defining the area in the Vatta-Maklár trench seems to be confirmed by our finds spatially correlating the identified medial occurrences (around Eger town or Báj Stream) and the distal (Ipolytarnóc) site, in agreement with the systematic decrease of thickness data of pumice-fall deposit (Unit C) from three sites (see text and Fig. 1B).

(2) On the basis of the assumed source vent and the decrease of pumice-fall isopachs, a northwestward spread of the ignimbrite can be taken as the simplest geometric scenario. However, due to the poorly constrained vent area, the unknown options of symmetric (i.e. radial) vs asymmetric flow direction, as well as the unknown original distribution of the surficial pyroclastic deposits (that were significantly removed by long-term erosion), we cannot infer more detailed transport directions. The geometry depicted in Fig. 4. refers to the reality, but is probably only part of the whole original distribution.

(3) Getting to know the pyroclastic sequence of the Eger-Ipolytarnóc Ignimbrite, we can say that the majority of the known thicknesses observable in the field are incomplete. At medial sites closest to the vent (e.g. Eger Tuff Quarry, Láz-tető Quarry: Fig. 1), even the largest exposures (quarries) that reveal up to 30 m-thick ignimbrites show only a part of the total thickness. On the other hand, field relationships do not allow to distinguish easily between the two largest, successive Lower Tuff units: the Eger-Ipolytarnóc (17.25 Ma) and the somewhat younger Mangó Ignimbrites (17.05 Ma, see main text), which occur together in the Bükk Foreland Volcanic Area. Where we have clues for disctinction, the obtained values for the Eger-Ipolytarnóc Ignimbrite – using data of the Mining and Geological Survey of Hungary (MBFSZ) 1:100,000 geological map and considering relief geometry – show up to ca. 60 m thicknesses (see Fig. 4 in main text). However, even these surface points include sections where either the bottom is not exposed or the top has been truncated by erosion, thus the obtained thickness values should be considered as minimum estimates. Fortunately, two distinguished sites, the medial Báj Stream^3^ (ca. 34 km from vent) and the distal Ipolytarnóc^4^ (ca. 80 km from vent: Fig. 1), reveal total thicknesses of ca. 60 and 35/45 m, respectively (Fig. 4). At both sites, under- and overlying rocks crop out, making these figures likely (i.e. implying minor or nil erosion of the ignimbrite after emplacement).

To infer proximal thickness values, it may be pertinent to take the example of the 39.8 ka Campanian Ignimbrite^5^ with high-end VEI=7. For its distal sites (≥40 km from source), thickness values can reach 60 m (i.e. similar to our findings), and maximum extracaldera thickness, well-preserved at proximal sites, 80 m. On this basis, as our obtained surface data points are at least 12-15 km from the assumed vent, maximum thickness values of the Eger-Ipolytarnóc Ignimbrite at proximal sites may have reached 80 m (Fig. 4). Notably, the very high volume of the Campanian Ignimbrite (>450 km^3^ bulk, corresponding to >180 km^3^ DRE) is due to the radial pattern of the pyroclastic-flow deposits around a point-like caldera source, which may not hold true for the Eger-Ipolytarnóc Ignimbrite.

(4) Neotectonic (i.e. postvolcanic) movements of the North Pannonian Basin^6^ after ignimbrite deposition – in particular, (a) uplift of the Bükk Mountains, (b) uplift of the post-ignimbrite andesite volcanoes of the Mátra Mountains, (c) normal- and strike-slip faulting over large areas, and (d) intense subsidence of the Great Hungarian Plain in the south, hosting undivided Miocene tuff infill in boreholes – make it even more complicated to infer the original areal distribution of the ignimbrites.

Summarizing the above issues (2), (3), (4), the quantification of single eruptive units like the Eger-Ipolytarnóc Ignimbrite can be done only with great uncertainties. Previous studies suggested thousands of km^3^ erupted material and up to 50,000 km^2^ areal coverage, without specific calculations^7,8^. Here, starting from constraints obtained for the Eger-Ipolytarnóc Ignimbrite, we give a minimum estimate for the area and erupted volume.

The extrapolated distribution of surface coverage of the Eger-Ipolytarnóc Ignimbrite using QGIS 3.2 Software defines an area of ~1650 km^2^ (Fig. 4). The aproximate volume of the bulk pyroclastic deposit has been calculated based on the following formula: *A x h_avg_=V*, where A is the polygon of the covered area, and h_avg_ is an average thickness of the ignimbrite, i.e. (80-40)/2 = 60 m. The result, i.e. the volume of the bulk ignimbrite, is 99 km^3^.

There are a number of worldwide examples of large-volume (VEI=7 and 8) silicic explosive eruptions (see tabulated data below), where bulk rock (total erupted tephra) volume and DRE (Dry Rock Equivalent, i.e. magma volume) is known, and their ratio can be calculated. We selected examples showing end members, which define an average ratio of 1.7. Ratios close to 1 commonly include (a big amount of) welded ignimbrites, the density of which is high. As we show in the main text, welded facies is missing for the Eger-Ipolytarnóc Ignimbrite. Thus, the average ratio of 1.7 seems to be likely; if applied to the Eger-Ipolytarnóc Ignimbrite, it results in 58 km^3^ DRE. However, for reasons discussed above, both the bulk and the DRE figures are minimum estimates, and real values may have been several times larger.

Tabulated data showing calculations of bulk rock / DRE volumes of selected large-volume ignimbrites worldwide. Data mainly from Crosweller et al.^9^

| **Supervolcanic (VEI 8) or VEI 7 eruption events** | **Location** | **VEI** | **Eruption Age** | **Composition of magma** | **Approximate volume of bulk rock (km3)** | **Approximate volume in DRE (km3)** | **Ratio of bulk rock / DRE** | |
| --- | --- | --- | --- | --- | --- | --- | --- | --- |
| Youngest Toba eruption | Sumatra, Indonesia | 8 | 75 Ka | Dacite-Rhyolite | 13200 | 5300 | 2.5 | |
| Huclkeberry Ridge eruption | Yellowstone, USA | 8 | 2.1 Ma | Rhyolite | 2450 | 940 | 2.6 | |
| Lava Creek eruption | Yellowstone, USA | 8 | 640 Ka | Ryolite | 1000 | 900 | 1.1 | |
| Aso-4 eruption | Kyushu, Japan | 8 | 87 Ka | Dacite-Rhyolite | 640 | 411 | 1.6 | |
| Oruanui eruption | Taupo, New Zealand | 8 | 25 Ka | Rhyolite | 1170 | 530 | 2.2 | |
| Mesa Falls eruption | Yellowstone, USA | 7 | 1.3 Ma | Rhyolite | 300 | 280 | 1.1 | |
| Bishop Tuff eruption | Long Valley, USA | 8 | 760 ka | Rhyolite | 790 | 625 | 1.3 | |
| Minoan eruption | Santorini, Greece | 7 | 3.6 Ka | Dacite-Rhyolite | 123 | 82 | 1.5 | |
| Campanian Ignimbrite | Campi Flegrei, Italy | 7 | 39.8 Ka | Trachyte-Phonolite | 528 | 211 | 2.5 | |
| **Average ratio of bulk rock / DRE:** | | | | | | | | **1.7** |

**References**

^1^Szakács, A., Márton, E., Póka, T., Zelenka, T., Pécskay, Z., Seghedi, I. Miocene acidic explosive volcanism in the Bükk Foreland, Hungary: identifying eruptive sequences and searching for source locations. *Acta Geol. Hung.* **41,** 413–435 (1998).

^2^Aguirre-Diaz, G.J., Labarthe-Hernández, G. Fissure ignimbrites: Fissure-source origin for voluminous ignimbrites of the Sierra Madre Occidental and its relationship with Basin and Range faulting. *Geology*, **31(9),** pp.773-776 (2003).

^3^Varga, Gy. (ed). A Mátra hegység földtana (Geology of the Mátra Mts: in Hungarian with English abstract). *Hung. Geol. Inst.,* **LVII, 1,** 575 p. (1975).

^4^Bartkó, L. Geology of Ipolytarnóc. *Geologica Hungarica series, Palaeontologica*, **44,** 16-71 (1985).

^5^Silleni, A., Giordano, G., Isaia, R. and Ort, M. H.: The Magnitude of the 39.8 ka Campanian Ignimbrite Eruption, Italy: Method, Uncertainties and Errors. *Front. Earth Sci.* **8,** 543399 (2020).

^6^Cloetingh, S. A. P., Horváth, F., Bada, G., Lankreijer, A. C. Neotectonics and surface processes: the Pannonian Basin and Alpine/Carpathian System. *EGU Stephan Mueller Publication Series,* **3,** 1-295 (2002).

^7^Szakács, A, Karátson, D. The Inner Carpathian calc-alkaline volcanism. In: Karátson. D. (ed) The land that is Hungary – geology, geography and cartography of Hungary and its surroundings. *CD-ROM, Arcanum Publishing House* (2002).

^8^Lukács, R., Harangi, S., Guillong, M., Bachmann, O., Fodor, L., Buret, Y., Dunkl, I., Sliwinski, J., von Quadt, A., Peytcheva, I., Zimmerer, M. Early to Mid-Miocene syn-extensional massive silicic volcanism in the Pannonian Basin (East-Central Europe): Eruption chronology, correlation potential and geodynamic implications. *Earth Sci. Rev.,* **179,** 1-19. (2018).

^9^Crosweller, H.S., Arora, B., Brown, S.K. et al. Global database on large magnitude explosive volcanic eruptions (LaMEVE). *J Appl. Volcanol.* **1,** 4 (2012).
