# Supplementary material for: Large-magnitude (VEI ≥ 7) ‘wet’ explosive silicic eruption preserved a Lower Miocene habitat at the Ipolytarnóc Fossil Site, North Hungary
Source: Sci Rep. 2022 Jun 13;12:9743. doi: 10.1038/s41598-022-13586-3 (PMC9192734; doi:10.1038/s41598-022-13586-3)
Supplement: Supplementary file 13 — Supplementary Information Summary. [file 41598_2022_13586_MOESM13_ESM.doc]

Supplementary material summary information (files uploaded separately):

**Supplement 1:** BSE images and vesicularity assessment (responsible author: Balázs Kiss)

***Supplementary table I:*** Main elements chemical composition and TAS diagram of pumice glass shards from Unit A and C

***Supplementary table II:*** 2D vesicularity statistics of pumices for Unit A and Unit C

**Supplement 2:** Major (EPMA) and trace (LA-ICP-MS) elements geochemistry of pumice glass shards – methodology and tabulated data sheets (responsible author: Maxim Portnyagin)

**Supplement 3:** U-Pb methodology, plots and data sheets (responsible author: Jean-Louis Paquette)

**Supplement 4:** Ar-Ar methodology, plots, data sheets and full report of results and interpretation (author: Matt Heizler, collaborative chief researcher)

**Supplement 5:** Volume estimation of the Eger-Ipolytarnóc Ignimbrite (responsible author: Dávid Karátson, Zoltán Cseri)
